# Early human occupation of Australia’s eastern seaboard

**DOI:** 10.1038/s41598-024-52000-y

**Published:** 2024-01-31

**Authors:** Shaun Adams, Kasih Norman, Justine Kemp, Zenobia Jacobs, Michael Costelloe, Andrew Fairbairn, Richard Robins, Errol Stock, Patrick Moss, Tam Smith, Serena Love, Tiina Manne, Kelsey M. Lowe, India Logan, Michael Manoel, Karen McFadden, Darren Burns, Thomas Dooley, Zac Falkiner, Chris Clarkson

**Affiliations:** 1 Australian Research Centre of Human Evolution, Griffith University, Nathan, QLD 4111 Australia; 2 Everick Foundation, 9/110 Mary St, Brisbane, QLD 4000 Australia; 3 Centre for Archaeological Science, School of Earth, Atmospheric and Life Sciences, University of Wollongong, Wollongong, NSW 2522 Australia; 4 Australian Research Council Centre of Excellence for Australian Biodiversity and Heritage, Canberra, Australia; 5 Quandamooka Yoolooburrabee Aboriginal Corporation, 100 East Coast Rd, Dunwich, QLD 4183 Australia; 6 School of Social Science, The University of Queensland, St Lucia, QLD 4072 Australia; 7 Triple-E Consultants, Tarragindi, QLD 4121 Australia; 8 School of the Environment, The University of Queensland, St Lucia, QLD 4072 Australia; 9 Max Planck Institute of Geoanthropology, Kahlaische Strasse 10, 07745 Jena, Germany; 10 Department of Archaeology, Max Planck Institute for the Science of Human History, Kahlaische Strasse 10, 07745 Jena, Germany

**Keywords:** Archaeology, Cultural evolution

## Abstract

Secure archaeological evidence for human occupation on the eastern seaboard of Australia before ~ 25,000 years ago has proven elusive. This has prompted some researchers to argue that the coastal margins remained uninhabited prior to 25 ka. Here we show evidence for human occupation beginning between 30 ± 6 and 49 ± 8 ka at Wallen Wallen Creek (WWC), and at Middle Canalpin Creek (MCA20) between 38 ± 8 and 41 ± 8 ka. Both sites are located on the western side of Minjerribah (North Stradbroke Island), the second largest sand island in the world, isolated by rising sea levels in the early Holocene. The earliest occupation phase at both sites consists of charcoal and heavily retouched stone artefacts made from exotic raw materials. Heat-treatment of imported silcrete artefacts first appeared in sediment dated to ~ 30,000 years ago, making these amongst Australia’s oldest dated heat-treated artefacts. An early human presence on Minjerribah is further suggested by palaeoenvironmental records of anthropogenic burning beginning by 45,000 years ago. These new chronologies from sites on a remnant portion of the continental margin confirm early human occupation along Sahul’s now-drowned eastern continental shelf.

## Introduction

**Figure 1:**
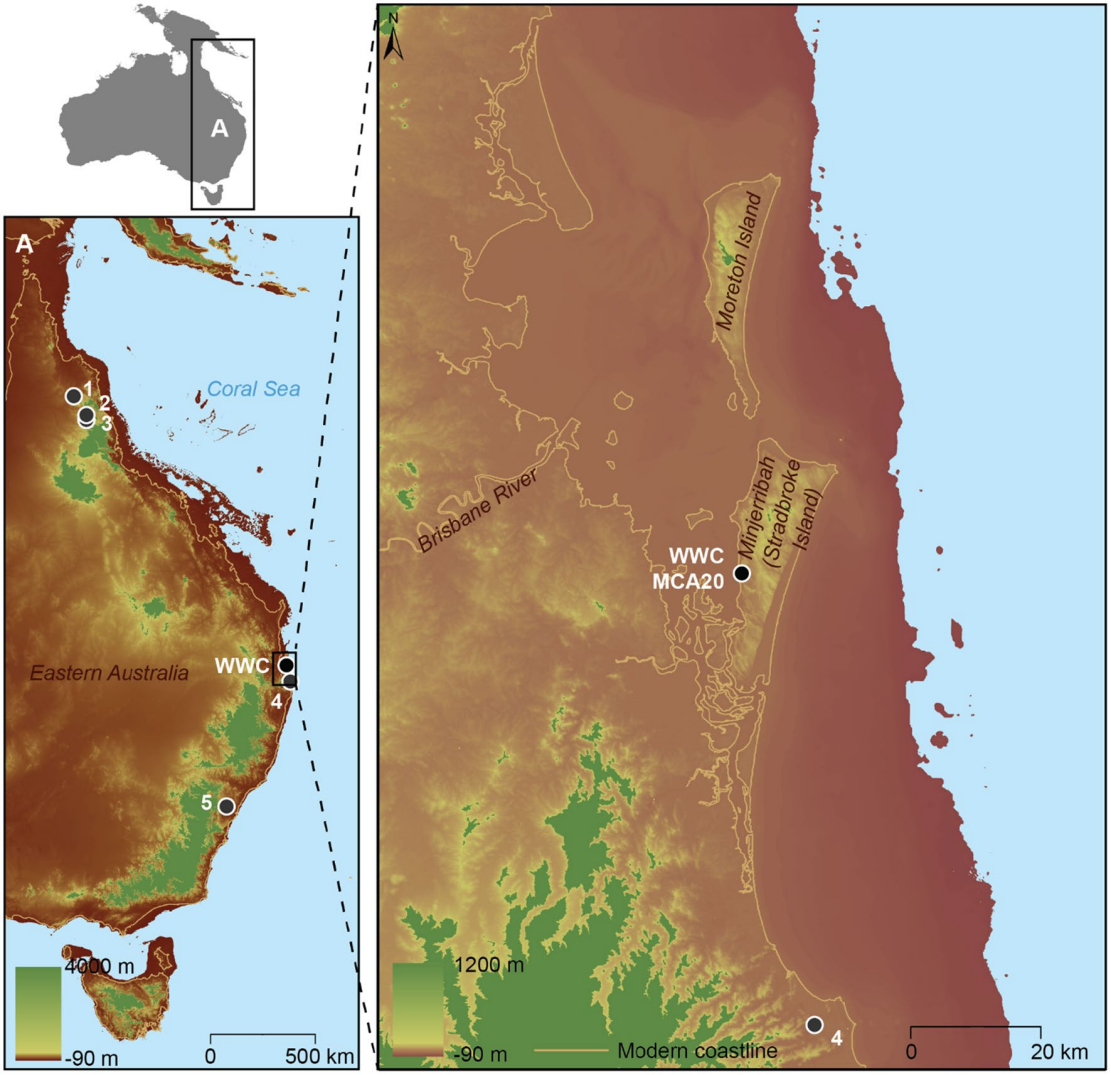
Eastern seaboard of Australia showing the c. 45 ka coastline^11^, with the position of Wallen Wallen Creek (WWC) and Middle Canalpin Creek (MCA20) indicated. Sites with secure archaeological evidence for human occupation greater than 30 ka that are located within 150 km of the eastern seaboard are numbered: 1. Hay Cave, 2. Ngarrabullgan Cave, 3. Nonda Rock, 4. Cobaki sand ridge, 5. PT 12 (Sydney Basin). Figure 1 was generated using Esri ArcMap v. 10.4.1. Bathymetric and topographic data: Australian Bathymetry and Topography Grid^12^ High-resolution depth model for the Great Barrier Reef—30 m^13^.

The eastern seaboard of Australia is the most densely populated region of the continent today. However, evidence of an early human presence has proven elusive despite half a century of archaeological research^1^. For almost 40 years, the oldest site on the eastern seaboard has been Wallen Wallen Creek (WWC), dated to 21,800 ± 400 BP^2^ (27,025–25,270 cal BP—all ages hereafter reported at 95.4% CI) on Minjerribah (North Stradbroke Island) in Quandamooka Country. Until now, only five other archaeological sites located within ~ 150 km of the eastern seaboard have initial occupation ages greater than 30,000 years before present (30 ka) (Fig. 1). Three of these cluster in tropical Northern Queensland: Ngarrabullgan Cave (41.7–39.1 cal ka BP), Nonda Rock (< 40 ka), and Hay Cave (34.2–29.7 cal ka BP)^3–5^. However, all of these sites are located west of Australia’s Great Dividing Range and far from the Pleistocene coast. The only two sites found east of the dividing range are the Cobaki sand ridge (39.3 ± 13 ka)^6^ located ~ 75 km south of WWC, and the PT 12 sand body (36.0 ± 12.0 ka) located in the Sydney Basin^7^. Other pre-Last Glacial Maximum sites from south-eastern Australia include Burril Lake, New South Wales (20.8 ± 0.8 ka)^8^, and Bend Road, Victoria (35.3 ± 2.6 ka)^9^. The rarity of sites on the coastal margin with occupation ages greater than 30 ka has prevented the development of an understanding of early settlement and resource use along the eastern continental margins where late Pleistocene population density is predicted to have been high^10^.

Here we report the results of new Indigenous community-led excavations at two sites on Minjerribah focusing on the age of initial occupation and subsequent cultural responses to major climate changes and sea level fluctuation.

### Excavations and stratigraphy

**Figure 2:**
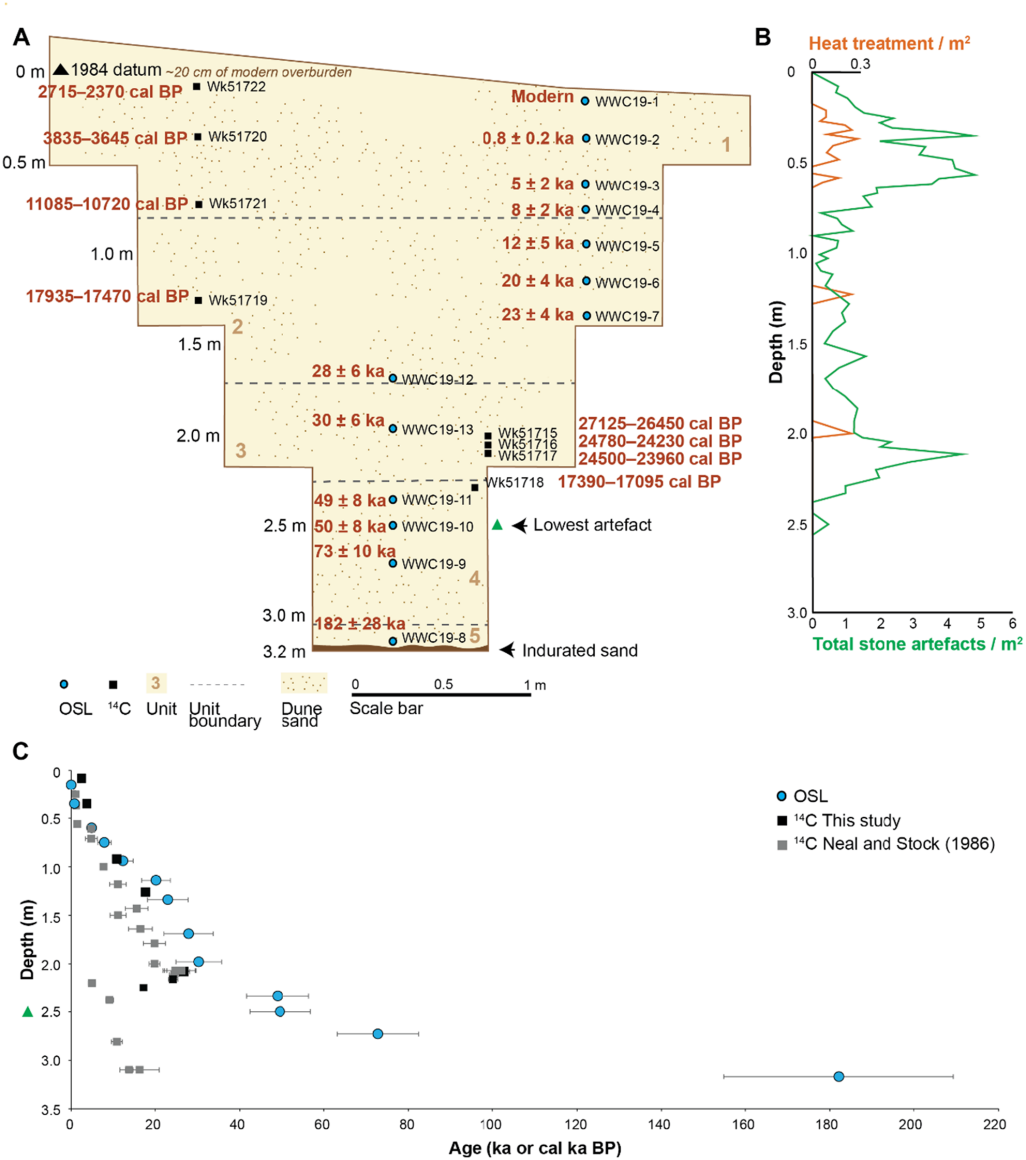
(**A)** East wall of the Wallen Wallen Creek excavation pit. The locations of the OSL samples are shown as blue circles and ^14^C samples collected in this study as black squares. All age estimates are shown at the 95.4% CI. The stratigraphic unit boundaries of Neal and Stock^2^ are indicated with grey stippling. (**B)** Total stone artefact and heat treatment counts for the site are plotted as artefacts per m^2^ as original excavation volumes were not available. (**C)** Age-depth plot showing OSL and radiocarbon age estimates for Wallen Wallen Creek (95.4% probability); depths are the mid-point depth of the range.

Excavations were first carried out at the site of WWC (− 27.573524 S, 153.41573 E) in 1985 by Rob Neal^2^ and revealed an Indigenous Australian occupation site situated on the western foot slope of a well-vegetated relict parabolic dune around 400 m from the current western shoreline of Minjerribah (Figure S1A, B). At the time of its first occupation, Minjerribah was one of a series of low hills beside now-drowned plains and river valleys. The excavation was stepped every metre and continued to a total depth of 320 cm, with only two 100 × 100 cm squares excavated at the base (Fig. 2A). Shoring was put in place for each step (Figure S1A, B). The original excavators interpreted the stratigraphy as a continuous occupation sequence containing five stratigraphic units. Unit 5 (305–320 cm) was a culturally sterile white sand overlying indurated sand. Unit 4 extended from 225 to 305 cm and consisted of white sand containing artefacts of non-local origin. Unit 3 extended from 170 to 225 cm and consisted of white sand rich in stone artefacts and charcoal including in situ flaking and hearths. Unit 2 extended from 80 to 170 cm and was comprised of light grey sand containing charcoal, stone artefacts and bone fragments. Unit 1 comprised of 80 cm of dark humic sand and a sparse midden containing charcoal, stone artefacts, shell and bone. Radiocarbon ages from the original excavation increased in age with depth down to ~ 210 cm depth, with an age of 21,800 ± 400 BP (OxA-806) (27,025–25,270 cal BP) at 207–201 cm depth (Table S1). Below 210 cm depth, radiocarbon ages are inverted. Stone artefacts were found at WWC to a depth of 250 cm (Fig. 2C), approximately 70 cm above indurated sand.

Uncertainty over the age of the lowest artefacts at the site prompted re-excavation in 2019, led by Quandamooka Yoolooburrabee Aboriginal Corporation (QYAC). This involved relocating the original 400 × 400 cm excavation area using ground penetrating radar (Figure S1C, Supplementary Information Sect. 11), emptying the original stepped pit (Figure S1D), cleaning the upper walls, re-bracing the original shoring in lower steps where the walls were fragile, and collecting new samples. Samples were collected for radiocarbon and optical dating, flotation, magnetic susceptibility and pollen analysis. The original stone artefact, faunal and archaeobotanical assemblages were also reanalysed.

**Figure 3:**
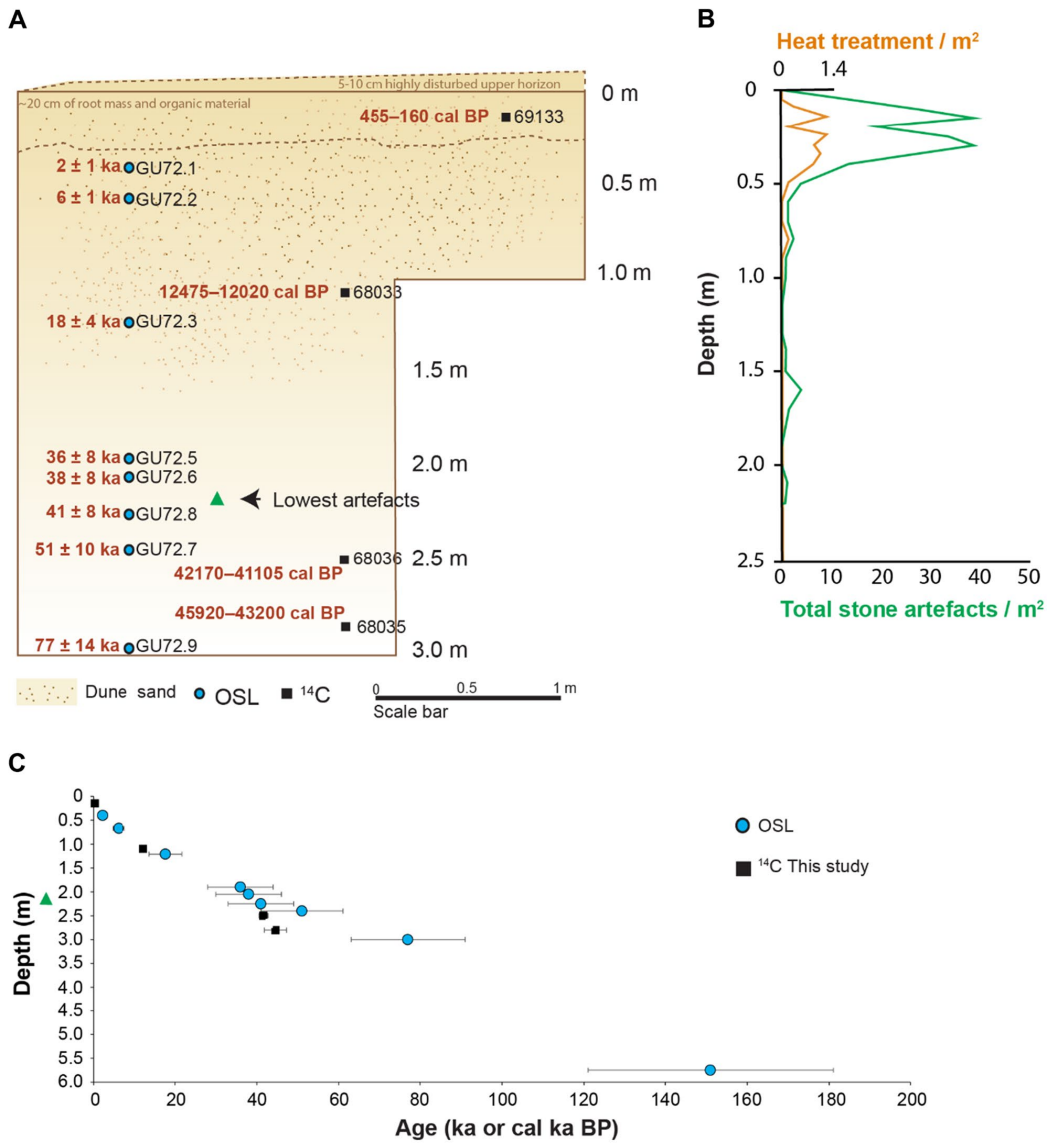
(**A)** Stratigraphic and geoarchaeological framework of MCA20 excavation with the location of the OSL and radiocarbon age estimates (95.4% CI) shown. (**B)** Total stone artefact counts and heat treatment counts for the site are plotted for MCA20 as artefacts per m^2^ to be comparable with WWC. (**C)** Age-depth plot showing OSL and radiocarbon age estimates for Middle Canalpin Creek (95.4% probability); depths are at the mid-point depth of the range.

To better understand Pleistocene occupation on Minjerribah, areas of a similar geomorphological context to WWC were modelled^6^, leading to the excavation of a 300 × 200 cm pit at Middle Canalpin Creek A (MCA20) (− 27.607971 S, 153.415986 E) in 2020 (Figure S2A, B). MCA20 is within a large flat sandy swale at the foot of a transgressive sand sheet of parabolic dunes ascribed to the mid-Pleistocene^6^. The site is bounded on the west by a large freshwater wetland extending 720 m to coastal mangroves. The excavation exposed 300 cm of weakly cemented fine, aeolian and colluvial sand below 14 cm of loose, very weakly cemented organic fine sand (Fig. 3A, Figure S2C). Under this surface layer, a weakly organic soil horizon has developed with an irregular lower boundary at 70 cm suggesting occasional tree throw or other minor disturbances. Artefacts continued to a depth of 215 cm (Fig. 3B). Informed consent has been obtained to publish identifiable images of subjects in an online open-access publication.

### Chronology

We constructed chronologies for WWC and MCA20 using radiocarbon (^14^C) dating of shell and charcoal and single-grain optically stimulated luminescence (OSL) dating of quartz grains. Most charcoal samples are small fragments which become less common with depth. We obtained OSL age estimates for 13 sediment samples from WWC (Fig. 2). Sediments at a depth of 317 cm (WWC19-8, Unit 5) yielded an age of 182 ± 27 ka. Above Unit 5, sediments accumulated, with a possible hiatus at 220 cm, continuously from 73 + /10 ka to the present day (WWC19-1, top of Unit 1) (Fig. 2A,C). The deepest sample for OSL dating (WWC19-8) was collected from the base of the excavation in Unit 5 (305–320 cm) immediately above the underlying indurated sand. Three samples were collected from Unit 4 (225–305 cm); one (WWC19-9) from the middle of the unit with an OSL age of 73 ± 10 ka, and two samples (WWC19-10 and − 11) from the upper half of the unit, with statistically consistent OSL ages of 50 ± 7 ka and 49 ± 7 ka (Table S5). The single sample collected from the centre of Unit 3 (170–225 cm) gave an age of 30 ± 5 ka (WWC19-13). Four samples were collected from Unit 2 (80–170 cm), with the sample dating the base of the unit giving an age of 28 ± 6 ka (WWC19-12). The middle of the unit gave ages of 23 ± 5 (WWC19-7) and 20 ± 4 ka (WWC19-6). The upper part of the unit is dated to 12 ± 2 (WWC19-5), close to the onset of the Holocene. Finally, deposition of Unit 1 (0–80 cm), also comprising the shell midden, ranges from 8 ± 2 ka (WWC19-4) to recent (WWC19-1). Radiocarbon ages obtained in 2019 (Supplementary Table S2) are different from those obtained during the 1985 excavation (Supplementary Table S1). Below 210 cm, all radiocarbon age estimates are too young (Fig. 2C).

At MCA20, OSL ages obtained for eight samples collected from the colluvial sand mantle display good internal consistency (Fig. 3). Ages increase with depth from 2.2 ± 0.6 ka at 40 cm, increasing to 6.2 ± 1.4 ka at 67 cm and 17.7 ± 4 ka at 121 cm. Below 121 cm, the sedimentation rate is lower with ages increasing to 36 ± 8 ka at 190 cm, 38 ± 8 ka at 205 cm, 41 ± 8 at 225 cm, 51 ± 10 ka at 240 cm, and 77 ± 14 ka at 300 cm depth. The OSL sample at 575 cm in the adjacent auger hole yielded an age of 151 ± 30 ka, which is consistent with ages reported for the Yankee Jack morphostratigraphic dune unit on Minjerribah and nearby sand islands^14–17^. Similar to WWC, radiocarbon diverges from OSL ages below ~ 200 cm depth (Fig. 3C).

### Cultural materials

**Figure 4:**
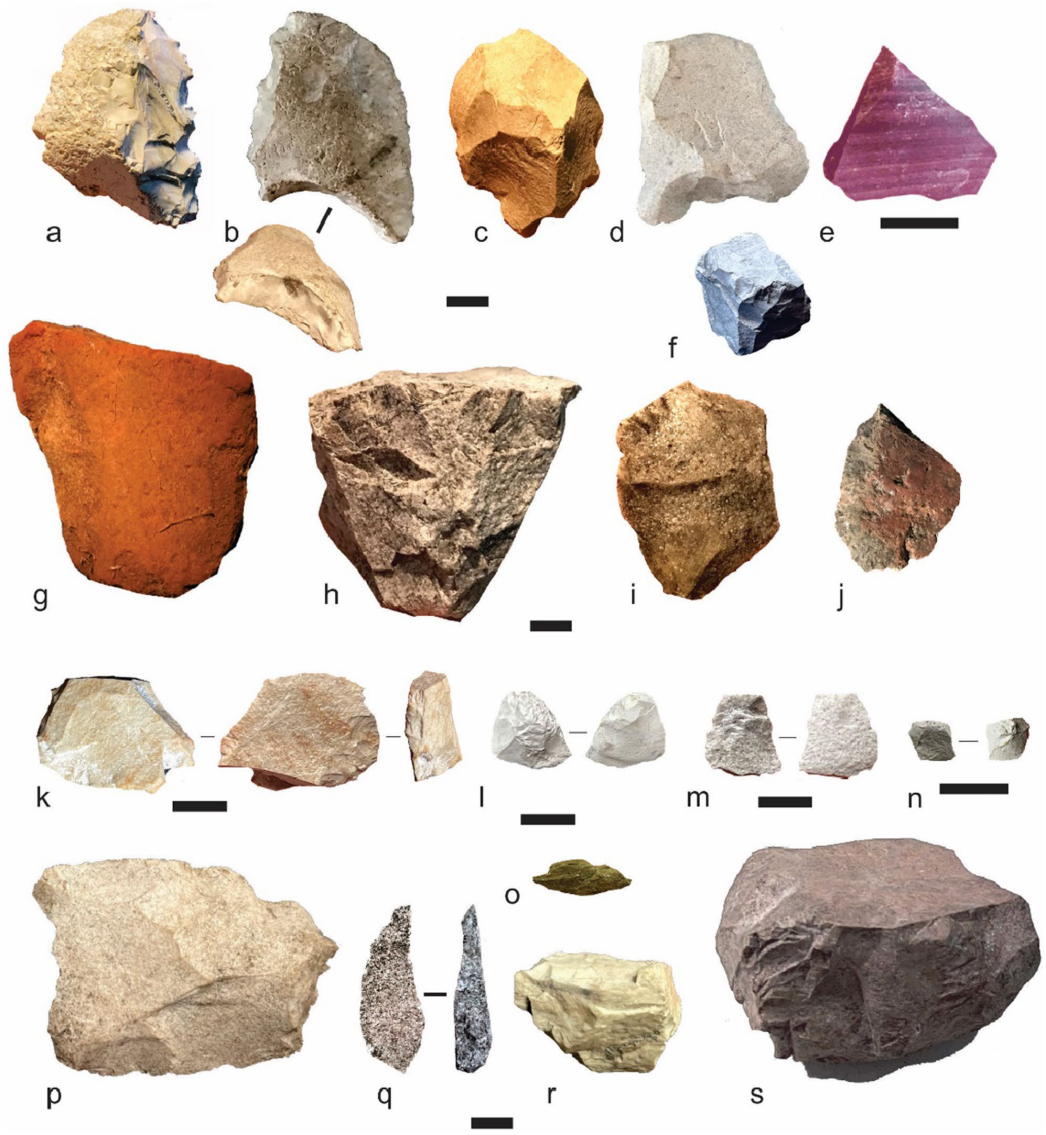
Examples of artefacts from the upper and lower assemblages from WWC and MCA20. **WWC:** (**a)** chert scraper, 220 cm bs; (**b)** chert notched scraper, 207 cm bs; (**c)** chert notched scraper, 207 cm bs; (**d)** silcrete notched scraper, 200 cm bs; (**e)** exotic banded hydrothermal chert flake, 220 cm bs; (**f)** chert multiplatform core, 220 cm bs; (**g)** ground ochre, 133 cm bs; (**h)** silcrete single platform core, 52 cm bs; (**i)** silcrete bipolar flake, 48 cm bs; (**j)** basalt ground edge axe flake, 52 cm bs; (**k)** bifacially retouched possibly heat treated silcrete scraper, 229 cm bs; (**l)** partially desilicified retouched flake with edge damage, 220 cm bs; (**m)** silcrete flake, 229 cm bs; (**n)** desilicified chert heat spall, 250 cm bs; **MCA20: o** green stone edge ground axe flake, 15 cm bs; (**p)** silcrete bifacial notched scraper, 15 cm bs; (**q)** silcrete asymmetric backed artefact, 25 cm bs; (**r)** chert end scraper, 220 cm bs; (**s)** silcrete multiplatform core, 35 cm bs.

The 1665 stone artefacts recovered from WWC occur to a depth of 250 cm (Fig. 2, Supplementary Table S9). Artefact densities are expressed as metres squared throughout as the excavated area is recorded but the original excavated volume per spit is unknown. Two main peaks in artefact deposition are apparent, defined as artefact densities ≥ 2/m^2^ (background density excluding peaks ≥ 2 artefacts/m^2^ is 0.84 artefacts/m^2^). The upper peak occurs between 25 and 61 cm depth (*n* = 1030, density = 3.46 artefacts/m^2^), corresponding with the shell midden (Unit 1). The upper peak is bracketed by radiocarbon and OSL age estimates, which date the increase in artefact numbers to the mid-Holocene, with artefact numbers declining by the late Holocene. This assemblage contains retouched artefacts (scrapers and notches) (Fig. 4A–D), ground edge axe flakes (Fig. 4J), and several cores and bipolar artefacts (Fig. 4I) with local quartz and exotic silcrete, the dominant raw materials with some chert and other materials (Fig. 4l–r, Figure S7 and S8, Figure S1).

**Figure 5:**
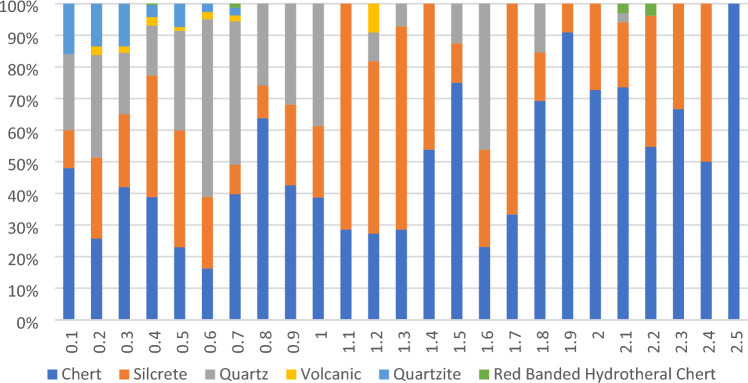
WWC raw material changes with depth.

The lower peak (*n* = 114, density = 2.63 artefacts/m^2^) at WWC occurs between 205 and 225 cm depth, bracketed by ages of 30 ± 5 ka (top) and 49 ± 7 ka (base). A pronounced trough in artefact deposition occurs between these upper and lower peaks corresponding to Marine Isotope Stage 2 (MIS2: 29–11.7 ka). Raw materials change from predominantly silcrete (Fig. 4h), quartz and quartzite in the upper peak to chert with some silcrete in the lower peak, with exotic high-quality banded hydrothermal chert from the Gold Coast hinterland area occurring only at 205–216 cm depth in the lower artefact peak (Fig. 4e) (Fig. 5). A single chert core occurs at 207 cm depth (Fig. 4f). Heat treated silcrete, as determined by surface colour, roughness from 3D scans, lustre and heat-induced fractures as well as replica tape and 3D scanning^18,19^, occurs in both lower and upper peaks in artefact deposition down to a depth of 212 cm, below an OSL age of 30 ± 5 ka. A single bifacially retouched silcrete flake at 229 cm depth also shows signs of heating with orange mottling, glossy surfaces and a heat break with rough surface. Imported red ochre occurs down to 211 cm depth but the lowest ground piece is found at 133 cm depth (Fig. 4g).

Differences are evident between the stone artefacts found in the lower peak in artefacts in Unit 3 versus artefacts in Unit 4, although sample size remains too small to perform statistical significance testing in most cases. The most pronounced difference is found between the proportion of heat spalling between units, with 33% of artefacts in Unit 4 exhibiting pot lidding, crenated fractures and heat breaks, versus only 6% in Unit 3. Desilicification through weathering causing friability of artefacts is slightly more common in Unit 4 (44% vs 36%), while silcrete is also slightly more common in Unit 4 (44% vs 36%). Technological differences are also evident, including higher proportions of retouch in Unit 4 (11% vs 4%), while cortex is absent on artefacts in Unit 4 (11% in Unit 3). There is no evidence of size sorting between the lower peak in Unit 3 and artefacts found in Unit 4. No statistically significant differences occur for either mass (Mann–Whitney *U* = 583.5, df = 141, *p* = 0.929) or maximum dimension between units (Mann–Whitney *U* = 570.5, df = 141, *p* = 0.878). The lowest three artefacts found in Unit 4 are bracketed by optical ages of 49 ± 7 ka and 50 ± 7 ka (Fig. 4a–d).

At MCA20, artefacts occur to a depth of 215 cm, bracketed by OSL ages of 38 ± 8 and 41 ± 8 (Fig. 3, Figure S2d, e). A distinct upper peak in stone artefact discard is observed above 60 cm depth (*N* = 1241), bracketed by OSL ages of 2.2 ± 0.6 ka and 6.2 ± 1.4 ka. Artefact deposition occurs down to 110 cm, then again at 140–180 cm and finally between 200 and 215 cm depth with no artefacts found between these depths. Retouched flakes and cores appear throughout the deposit (Fig. 4p–s), whereas flakes from ground edge axes (Fig. 4o), bipolar artefacts, ground ochre and a single backed artefact occur only in the upper midden assemblage (Fig. 4q, Figure S9, Table S10. Raw material frequencies change through time (Table S11, Figure S11), with local quartz dominant throughout, silcrete most common between 100 and 150 cm depth, chert found only above 60 cm depth, and a rare white or white/pink chert found only in the lowest assemblage from 160–215 cm depth. This mirrors the pattern seen at WWC, with exotic high-quality chert imported in the earliest assemblage. Heat treated silcrete appears at MCA20 above 80 cm depth between 17.7 ± 4 ka and 6.2 ± 1.4 ka.

## Discussion and interpretation

Our reinvestigation yields two Pleistocene cultural assemblages composed of heavily reduced imported and exotic raw materials. The later industry at WWC and MCA20 shows typical mid-to-late Holocene assemblage elements such as bipolar artefacts, axe flakes and backed artefacts in association with midden formation as sea levels stabilised. WWC and MCA20 may represent segments of a semi-continuous site on remnant late-Pleistocene land surfaces that ran along the western edge of Minjerribah, dissected by spring-fed creeks and adjacent wetlands. The evidence for heating silcrete at WWC is dated to > 30 ± 5 ka (with one potential heated artefact in Unit 4, Fig. 4K), and was most likely undertaken to improve the flaking quality of the stone. We find this to be one of the oldest instances of heat treatment in Australia, alongside Burrill Lake rockshelter (25 ka) and the open sites in the Willandra Lakes in NSW (42–30 ka)^20^.

The age of first occupation at WWC remains uncertain. An apparent stratigraphic break, first identified in Neal and Stock^2^, occurs between Units 3 and 4 at a depth of 220 cm. The middle of Unit 3 has an OSL age estimate of 30 ± 5 ka (198 cm depth) which overlaps at two sigma with three radiocarbon ages (207–216 cm depth) which derive from 4 to 13 cm above the base of Unit 3. An OSL age estimate of 49 ± 7 ka occurs at 234 cm depth (14 cm below the Unit 3/4 boundary). The unit boundary roughly corresponds to both the base of the lower peak in artefact deposition at 225 cm depth, and a significant reduction in excavated area from 8m^2^ to 2m^2^ (225 cm depth). Only nine artefacts occur in Unit 4 below the Unit 3 boundary down to a depth of 250 cm; however, this drop in artefact numbers is not simply due to a reduction in excavated area as a pronounced decrease in artefact density also occurs in Unit 4 from 1.85 artefacts/m^2^ (Unit 3) to 0.75 artefacts/m^2^. We speculate that either the Unit 3/4 boundary represents an old land surface with artefacts below this surface transported downwards by trampling or other processes, or, that the artefacts represent in situ discard at an earlier time. The Unit 4 assemblage, albeit small, differs in some characteristics from that of Unit 3 and shows no evidence of size sorting, hence in situ deposition cannot be entirely ruled out.

Similar low artefact densities (0.86/m^2^) occur at MCA20 below 50 cm depth, with small pulses of artefacts occurring to a depth of 215 cm. The lowest six artefacts at MCA20 are bracketed by OSL age estimates of 38 ± 8 and 41 ± 8 ka, with an artefact density of 0.75/m^2^. The lowest six artefacts at 200–215 cm depth occur 60 cm below the next artefact pulse located at 160 cm depth, which is bracketed by ages of 17.7 ± 4.0 at 121 cm and 36 ± 8 ka at 190 cm depth. It is unlikely that the lowest six artefacts were displaced downwards by 60 cm. This would seem to indicate low level occupation likely began at MCA20 between 38 ± 8 and 41 ± 8 ka. We note artefacts of a similar depositional age have been found to the south of Minjerribah at the Cobaki sand ridge. Here, a 40 cm thick cultural deposit containing 131 artefacts was overlayed by a peat unit. A TL age of 39 ± 13 ka was collected at the base of the 40 cm thick cultural deposit, in association with the lowest four artefacts^6^.

Possible evidence of early human occupation in the region also comes from Native Companion Lagoon, 14 km southwest of WWC and 7 km south of MCA20 with burning dated to 45 ka (Figure S23). This is followed by a marked decline in rainforest abundance on the island between 35 and 21 ka^21^ that may result from anthropogenic fire regimes coupled with cooler and drier conditions. Examination of charcoal records from two other sites across the island, Tortoise Lagoon (high dune central site) and Welsby Lagoon (northwest coastal site), indicate spatial variability in burning suggestive of Indigenous mosaic landscape management of the kind still practiced today^20^. These palaeoecological records suggest that sclerophyll forests and woodlands were the main ecosystems present^21–23^, and combined with the positive moisture balance, suggest an environment suitable for human occupation over at least the last 45 ka.

Our results show occupation of the eastern seaboard of Australia by at least 30 ka and perhaps as early as 45–49 ka. WWC, MCA20 and Cobaki are the closest sites to the late Pleistocene coastline on the eastern seaboard, while WWC and MCA20 are the only known examples of occupation of the now-drowned remnant Pleistocene coastal plain. Early ephemeral occupation of Minjerribah is characterised by long-distance transport of raw materials, high retouch intensity and sparse occupation likely indicative of high group mobility with anthropogenic firing of the landscape. Occupation appears to have been continuous and of low intensity throughout MIS 2, intensifying after the Holocene marine transgression with the establishment of a local marine-based economy.

### Supplementary Information

Supplementary Information.

## Data Availability

Data generated and/or analysed during the current study are included in this published article. Any further information is available through the corresponding author on reasonable request.
