# Author Correction: Early human occupation of Australia’s eastern seaboard

**DOI:** 10.1038/s41598-024-65370-0

**Published:** 2024-06-21

**Authors:** Shaun Adams, Kasih Norman, Justine Kemp, Zenobia Jacobs, Michael Costelloe, Andrew Fairbairn, Richard Robins, Errol Stock, Patrick Moss, Tam Smith, Serena Love, Tiina Manne, Kelsey M. Lowe, India Logan, Michael Manoel, Karen McFadden, Darren Burns, Thomas Dooley, Zac Falkiner, Chris Clarkson

**Affiliations:** 1 Australian Research Centre of Human Evolution, Griffith University, Nathan, QLD 4111 Australia; 2 Everick Foundation, 9/110 Mary St, Brisbane, QLD 4000 Australia; 3 Centre for Archaeological Science, School of Earth, Atmospheric and Life Sciences, University of Wollongong, Wollongong, NSW 2522 Australia; 4 Australian Research Council Centre of Excellence for Australian Biodiversity and Heritage, Canberra, Australia; 5 Quandamooka Yoolooburrabee Aboriginal Corporation, 100 East Coast Rd, Dunwich, QLD 4183 Australia; 6 School of Social Science, The University of Queensland, St Lucia, QLD 4072 Australia; 7 Triple-E Consultants, Tarragindi, QLD 4121 Australia; 8 School of the Environment, The University of Queensland, St Lucia, QLD 4072 Australia; 9 Max Planck Institute of Geoanthropology, Kahlaische Strasse 10, 07745 Jena, Germany; 10 Department of Archaeology, Max Planck Institute for the Science of Human History, Kahlaische Strasse 10, 07745 Jena, Germany

Correction to: *Scientific Reports* 10.1038/s41598-024-52000-y, published online 31 January 2024.

Thomas Dooley was omitted from the author list in the original version of this Article.

The Acknowledgements now reads:

“This research has been made possible by the extraordinary dedication of the Quandamooka People to ensuring their cultural heritage is appropriately recognised and protected. The Quandamooka Yoolooburrabee Aboriginal Corporation (QYAC) team who oversaw this research was led by Chairperson Prof. Valerie Cooms, Joint Management Coordinator Darren Burns and Senior Cultural Heritage Coordinator Michael Costelloe. The authors of this paper are both honoured and privileged to assist the Quandamooka People in documenting their remarkable heritage. These results are the culmination of decades of study by three pioneering researchers, Archaeologists Rob Neal, Dr Richard Robins and Geomorphologist Dr Errol Stock. Together, they have laid a foundation for the scientific understanding of what is a complex, dynamic and internationally significant environmental and cultural landscape. Vital financial support for this research was provided by QYAC, the University of Queensland Faculty of Humanities and Social Science, Everick Foundation and the Queensland Parks and Wildlife Service.”

The Author contributions now reads:

“RR and ES conceived the project. CC, KN and SA wrote the main text with specialist contributions from JK and ZJ. Figures by KN, CC, SA, PM and JK. CC, AF, KN, MC, DB, KM, TM and TS re-excavated WWC. SA, ZF, SL, MC, TS, KM and DB excavated MCA20. AF obtained funding for WWC and KM obtained funding for WWC and MCA20 excavation. TR contributed funding for WWC and MCA20 excavation and analyses. KN collected, prepared and measured the optical dating samples, and ZJ generated the optical ages at WWC. ZJ wrote the optical dating SI with assistance from KN. JK performed the optical dating at MCA20. CC analysed stone artefacts from WWC and MCA20. AF performed flotation and identified charcoal for radiocarbon dating from WWC. TM and TD conducted faunal analysis at WWC and MCA20. TS performed mollusc analysis from WWC and MCA20. KML performed GPR, magnetic gradiometry and magnetic susceptibility at WWC. MM identified heat treatment on silcretes from WWC and MCA20.”

The original Article has been corrected.

