# Supplementary material for: Early human occupation of Australia’s eastern seaboard
Source: Sci Rep. 2024 Jan 31;14:2579. doi: 10.1038/s41598-024-52000-y (PMC10830458; doi:10.1038/s41598-024-52000-y)
Supplement: Supplementary file 1 — Supplementary Information. [file 41598_2024_52000_MOESM1_ESM.docx]

**Supplementary Information**

1. Excavation Methods
2. WWC Radiocarbon Chronology
3. WWC Luminescence Chronology
4. MCA20 Radiocarbon Chronology
5. MCA20 Luminescence Chronology
6. WWC Lithics
7. MCA20 Lithics
8. WWC Fauna and Shell
9. MCA20 Fauna and Shell
10. Minjerribah Palaeoecology
11. WWC Geophysics
12. Supplementary References

- Informed consent has been obtained to publish identifiable images of subjects in an online open-access publication.

**1. Excavation methods**

The 1985 WWC pit was emptied, cleaned, photographed and new levels taken with a total station. All sample depths were tied into the original 1985 surface (indicated as 1985 datum in Fig. S1) spit levels taken as the northwest corner of the pit. A column sample was obtained for archaeobotany, sediment samples, magnetic susceptibility and pollen analysis. OSL and radiocarbon samples were collected. The original shoring could not be removed due to safety concerns and hence the sections could not be re-exposed for the lower three steps.

MCA20 consisted of a 300 cm x 200 cm excavation to 300 cm depth. The excavation was reduced to 200 cm x 200 cm in size from 60 cm depth. An auger hole 150 m south of MCA20 was logged and sampled to ascertain the age of the underlying dune sands. The excavation exposed 300 cm of weakly cemented fine, aeolian and colluvial sand below 14 cm of loose, very weakly organic fine sand that is interpreted as surficial layer of disturbance (Fig. S2).


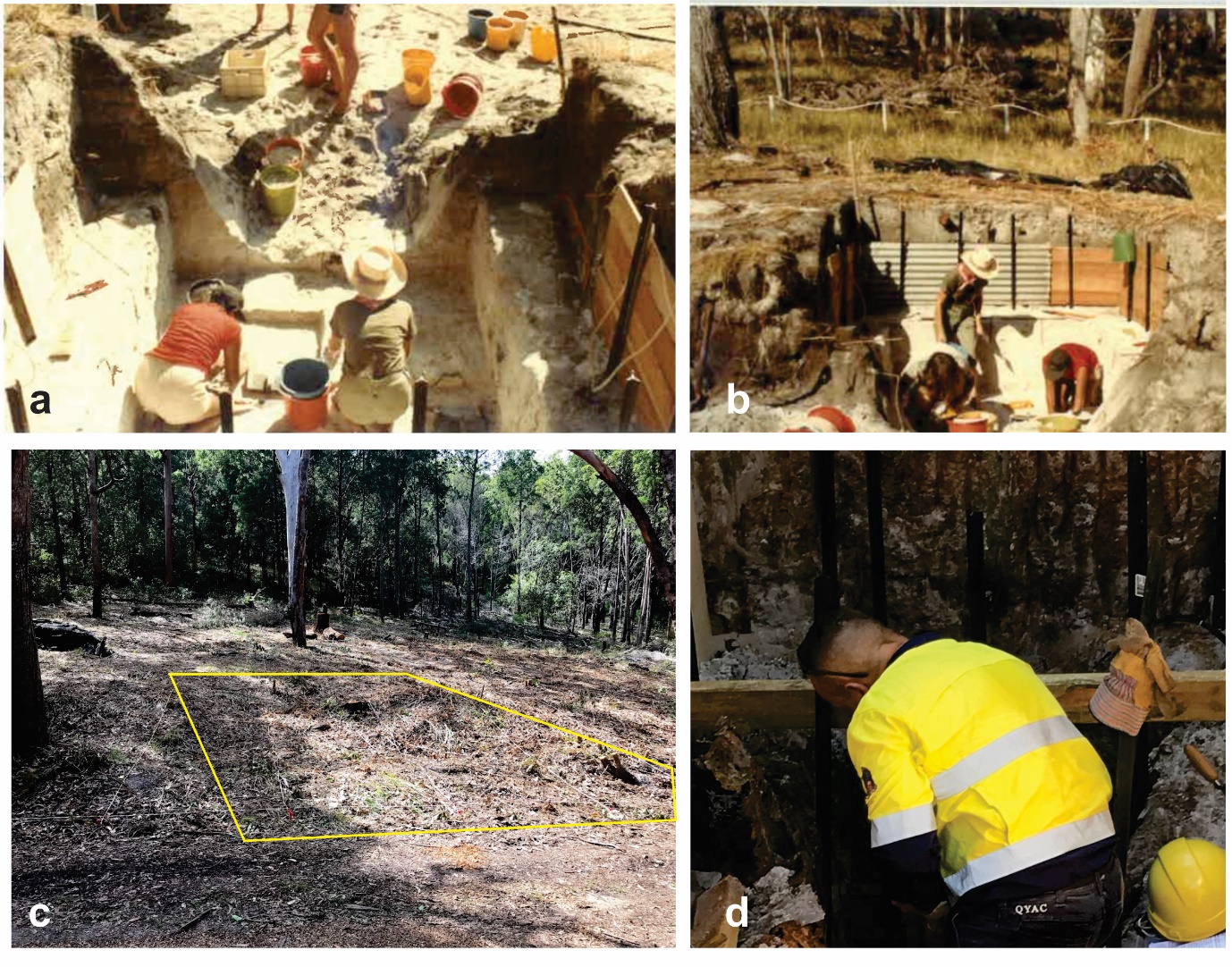


**Figure S1. a** The original 1985 Neal excavation in progress. **b** The 1985 sheet iron and wood shoring is put in place after the first step. **c** Location of the 1985 excavation as seen in 2019 after vegetation clearance by QYAC rangers. **d** QYAC Cultural Heritage Manager, Mr Michael Costelloe, empties backfill from the lowest step prior to collecting OSL samples.


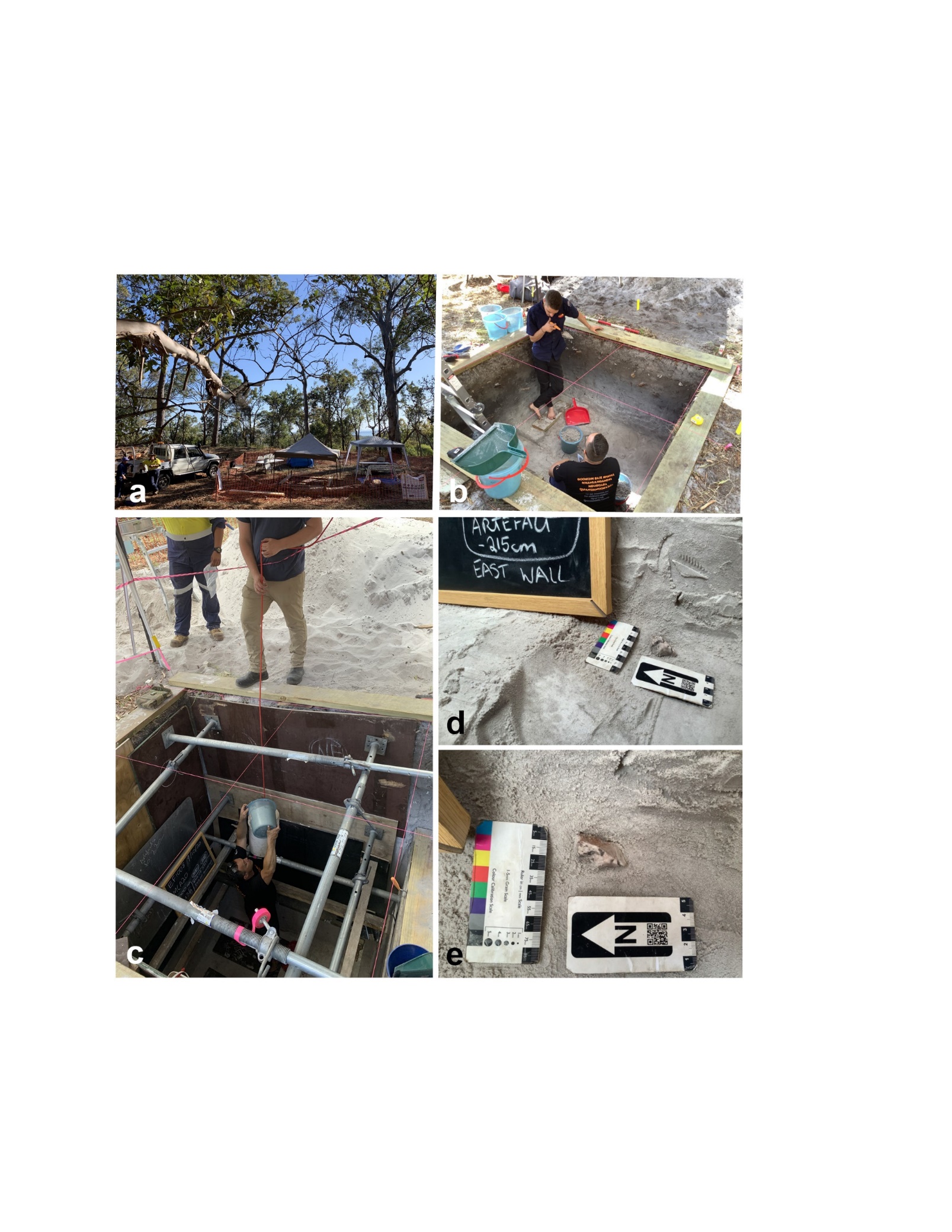


**Figure S2. a** MCA20 setting and position within a large sand swale overlooking Moreton Bay. **b** QYAC cultural heritage team excavating, note the transition from upper dark highly organic sands to weakly cemented white sands at ~120 cm. **c** QYAC Cultural Heritage Manager, Mr Michael Costelloe at 300 cm depth. **d** Lowest artefacts from MCA20 recovered in-situ from 215 cm. **e** Pink chert core recovered from 215 cm depth.

**2. Wallen Wallen Creek Radiocarbon Chronology**

**Table S1:** Previously published radiocarbon ages for samples collected from WWC^[1,2]^.

| **Lab code** | **Area** | **Spit** | **Depth below 1985 datum (cm)** | **Technique^a^** | **Material^b^** | **Pretreatment^C^** | **^14^C age (years BP)** | **Calibrated age (cal yrs BP)^d^** | |
| --- | --- | --- | --- | --- | --- | --- | --- | --- | --- |
|  |  |  |  |  |  |  |  | **68.2% probability** | **95.4% probability** |
| SUA-2461 | ZH | 7 | 22-25 | GPC | Shell^$^ | Acid etch | 1070 ± 50 | 685–550 | 770–495 |
| SUA-2465 | ZH | 12 | 33-37 | GPC | Shell^$^ | Acid etch | 1230 ± 50 | 850–685 | 925–630 |
| SUA-2462 | ZD | 19 | 58-61 | GPC | Charcoal | ABA | 2120 ± 70 | 2130–1995 | 2310–1840 |
| SUA-2466 | ZC | 23 | 69-71 | GPC | Charcoal | ABA | 4290 ± 90 | 4960–4620 | 5260–4450 |
| SUA-2343 | ZC | 32 | 97-100 | GPC | Charcoal | ABA | 6950 ± 80 | 7840–7670 | 7930–7605 |
| SUA-2467 | ZD | 38 | 114-118 | GPC | Charcoal | ABA | 9760 ± 140 | 11255–10785 | 11620–10660 |
| SUA-2463 | X(U+V) | 43 | 138-143 | GPC | Charcoal | ABA | 13040 ± 220 | 15885–15245 | 16285–14940 |
| SUA-2342 | ZB | 47 | 158-164 | GPC | Charcoal | ABA | 13650 ± 240 | 16855–16125 | 17115–15735 |
| SUA-2464 | X/Z | 50 | 175-179 | GPC | Charcoal | ABA | 16420 ± 260 | 20140–19490 | 20445–19160 |
| SUA-2341 | XT | 56 | 201-207 | GPC | Charcoal | ABA | 20560 ± 250 | 25015–24330 | 25320–23985 |
| OxA-807 | ZK | 56 | 201-207 | AMS | Charcoal | ABA | 21430 ± 400 | 26025–25210 | 26795–24690 |
| OxA-806 | XT | 56 | 201-207 | AMS | Charcoal | ABA | 21800 ± 400 | 26475–25645 | 27025–25270 |
| OxA-808 | ZD | 59 | 217-220 | AMS | Charcoal | ABA | 4410 ± 80 | 5255–4845 | 5290–4830 |
| OxA-809 | ZD | 63 | 234-238 | AMS | Charcoal | ABA | 8200 ± 90 | 9275–9005 | 9425–8780 |
| OxA-810 | ZD | 70 | 256-281 | AMS | Charcoal | ABA | 9640 ± 120 | 11155–10775 | 11225–10585 |
| SUA-2344R | XV | 75 | 301-310 | GPC | sediment^^^ | Boil in HCl | 11990 ± 70 | 14015–13760 | 14035–13610 |
| OxA-1011 | XV | 75 | 301-310 | AMS | Charcoal | ABA | 13500 ± 400 | 16865–15700 | 17420–15100 |

^a^GPC = gas proportional counting; AMS = accelerator mass spectrometry

^b^Shell samples were from the Hercules Club mud whelk (*Pyrazus ebeninus*) and the sediment sample a peat (organic-rich) sediment. Charcoal samples were not identified to taxa or species in original publications.

^c^ABA = Acid Base Acid

^d^ All radiocarbon ages were calibrated using the SH20 calibration curve^[3]^, except those for the shell samples that were calibrated using the Marine20 calibration curve^[4]^ and a ΔR value of -156 ± 18 based on 10 datapoints located closest to WWC, sourced from calib.org/marine/. All age ranges are rounded to the nearest 5 years.

**Table S2:** New WWC radiocarbon ages obtained in 2019.

| **Lab code** | **Excavation** | **Area/Total Station Point** | **Spit** | **Depth below 1985 Datum**  **(cm)^a^** | **Material** | **F^14^C**  **(%)** | **^14^C age**  **(yrs BP)** | | **Calibrated ^14^C age (cal yrs BP)** | | | |  |
| --- | --- | --- | --- | --- | --- | --- | --- | --- | --- | --- | --- | --- | --- |
|  |  |  |  |  |  |  |  |  | **68.2% probability** | | **95.4% probability** | |  |
| Wk51722 | WW19 | Sample H | NA | 8 | Charcoal, Angiosperm wood | 73.2 ± 0.2 | | 2506 ± 21 | | 2705-2360 | | 2715–2370 | |
| Wk51720 | WW19 | Sample F | NA | 35 | Charcoal, Angiosperm wood | 64.6 ± 0.2 | | 3509 ± 20 | | 3825–3695 | | 3835–3645 | |
| Wk51721 | WW19 | Sample G | NA | 92 | Charcoal, Angiosperm wood | 30.3 ± 0.1 | | 9595 ± 24 | | 11070– 10765 | | 11085–10720 | |
| Wk51719 | WW19 | Sample E | NA | 126 | Wood charcoal, Proteaceae/Casuarinaceae | 16.3 ± 0.1 | | 14568 ± 35 | | 17885–17565 | | 17935–17470 | |
| Wk51715 | WWC | XH | 57 | 207-211 | Wood charcoal, Proteaceae/Casuarinaceae | 6.0 ± 0.1 | | 22549 ± 92 | | 27075–26495 | | 27125–26450 | |
| Wk51716 | WWC | M28A | 57 | 207-211 | Charred Monocot stem fragment (short-lived) | 7.8 ± 0.1 | | 20442 ± 67 | | 24625–24340 | | 24780–24230 | |
| Wk51717 | WWC | M28A | 58 | 211-216 | Charcoal, Angiosperm wood | 8.1 ± 0.1 | | 20215 ± 65 | | 24315–24080 | | 24500–23960 | |
| Wk51718 | WWC | M28A | 60 | 220-225 | Charcoal, Angiosperm wood | 17.0 ± 0.1 | | 14240 ± 34 | | 17350–17130 | | 17390–17095 | |

^a^Samples E-H were collected from a 2019 column sample and shot in with a total station to give depth below the 1985 datum. Samples from Spits 57-60 derive from a 1984 test excavation.

**3. Wallen Wallen Creek Optical (OSL) Chronology**

OSL dating provides a means of determining burial ages for sediments and associated artefacts and fossils^[5,6,7,8,9,10,11]^. The method is based on the time-dependent increase in the number of trapped electrons induced in mineral grains—such as quartz—by low levels of ionising radiation from the decay of natural uranium, thorium and potassium in the surrounding deposits, and from cosmic rays. The time elapsed since the light-sensitive electron traps were emptied can be determined from measurements of the luminescence signals from quartz (optically stimulated luminescence, OSL) from which the equivalent dose (D_e_) is estimated, together with determinations of the radioactivity of the sample and the material surrounding it to a distance of ~30 cm (the environmental dose rate). The luminescence ‘clock’ is reset by just a few seconds (quartz) of exposure to sunlight. The D_e_ divided by the environmental dose rate gives the burial time of the grains in calendar years ago.

We exploited the inherent benefits of single-grain dating that include the identification and elimination of individual grains that exhibit aberrant luminescence characteristics^[8,12]^ and the use of D_e_ distributions to investigate the potential impact that depositional and post-depositional processes, such as sediment mixing or insufficient exposure to sunlight (partial bleaching), may have on age determination.

**Sample collection**

Thirteen samples were collected for optical dating from depths of between 5 and 337 cm below surface (Table S1). Fig. 2 shows the position of all 13 samples in their sedimentary, stratigraphic and archaeological context. The re-opened 1985 excavation pit stepped down three times to the base, with samples collected from the vertical wall of each step. Samples OSL1–7 were collected from the southeast corner of the excavation from the exposed and cleaned profile with depths below surface measured using a tape measure and shot in with the total station. Samples OSL 8–13 were collected down the middle of the pit in the East wall and shot in with the total station. The original pit walls were shored with galvanised steel. Due to the instability of the dune sediments, these were left in place for safety during collection of the six deepest samples, and circular openings drilled through the steel to expose the sediment behind. The exposed profile wall was then scraped back at the sample locations to reduce pit fill contamination. Sample were collected at similar depths from which samples for radiocarbon (^14^C) dating was collected to facilitate direct comparisons, and also from deeper deposits down to the base of the excavation, that may be older than the effective limit of ^14^C dating. All samples were collected using 20 mm diameter sample tubes of 20 cm length and the tubes were gently hammered into the excavated profile wall. Once collected the samples were immediately sealed. The hole left after extraction of the sample tube was widened to allow insertion of a field gamma spectrometer (FGS) probe of either 1-inch or 2-inch diameter.

Sample field and lab codes, together with mid-point depths of the sample tubes below datum and below surface are provided in Table S1. Both below datum and below surface depths are provided for samples OSL 1–7 as the site slopes towards the south; the datum is based on measurements in the northeast corner of the excavation. Samples are presented in depth order.

**Table S3:** Sample field and lab codes, and depth below datum and surface.

| **Field Code** | **CABAH code** | **Depth below 1985 datum (cm)** | **Depth below surface (cm)^a^** |
| --- | --- | --- | --- |
| WWC19-1 | CABAH-536 | 15 | 5 |
| WWC19-2 | CABAH-537 | 35 | 25 |
| WWC19-3 | CABAH-538 | 60 | 50 |
| WWC19-4 | CABAH-539 | 75 | 65 |
| WWC19-5 | CABAH-540 | 94 | 84 |
| WWC19-6 | CABAH-541 | 114 | 104 |
| WWC19-7 | CABAH-542 | 134 | 124 |
| WWC19-12 | CABAH-547 | 169 | 169 |
| WWC19-13 | CABAH-548 | 198 | 198 |
| WWC19-11 | CABAH-546 | 234 | 234 |
| WWC19-10 | CABAH-545 | 250 | 250 |
| WWC19-9 | CABAH-544 | 273 | 273 |
| WWC19-8 | CABAH-543 | 317 | 317 |

^a^WWC19-1-7 depth below surface measured from a measuring tape from surface. WWC19-8-13 depth below surface measured from the 2019 NW corner surface of pit with the total station.

**Sample preparation and analytical facilities**

All samples were prepared using routine optical dating procedures^[6]^. Samples were first sieved to obtain a range of sand-sized grain fractions. Grains of 180–212 µm in diameter were used for dating. Grains were then treated with hydrochloric (HCl) acid and hydrogen peroxide (H_2_O_2_) solution to remove carbonates and organic matter, respectively. The quartz grains were etched using 40% hydrofluoric (HF) acid for 45 min to dissolve any remaining feldspar grains that may be present in the quartz separates, and to remove the alpha-irradiated layer around the surface of each grain. The HF-etched quartz grains were then rinsed in HCl acid to remove any precipitated fluorides and sieved again.

Single-grain OSL measurements of D_e_ were made for all quartz samples. OSL measurements were made on an automated Risø TL-DA-20 luminescence reader equipped with a focused green (532 nm) laser for single-grain stimulation^[13]^ Luminescence emissions were detected using a 9107Q-AP-TTL-03 photomultiplier tube. The OSL signals were detected through Hoya U-340 filters. Single-grain measurements were made using aluminium discs drilled with 100 holes, each 300 µm in diameter and 300 µm deep^[13].^ Irradiations were carried out inside each luminescence reader using ^90^Sr/^90^Y beta sources that have been calibrated using a range of known gamma-irradiated quartz. Spatial variations in beta dose rate to individual grain positions were taken into account for D_e_ determination^[14]^.

**Environmental dose rate determination and results**

The total environmental dose rate consists of contributions from beta, gamma and cosmic radiation external to the grains, plus a small alpha dose rate due to the radioactive decay of uranium and thorium inclusions inside sand-sized grains of quartz. To calculate the OSL ages, we have assumed that the present-day radionuclide activities and dose rates have prevailed throughout the period of sample burial.

We estimated the beta dose rates directly by low-level beta counting of dried, homogenised and powdered sediment samples in the laboratory, using a Risø GM-25-5 multi-counter system^[15]^. We prepared and measured samples, analysed the resulting data, and calculated the beta dose rates and their uncertainties following the procedures described and tested in Jacobs and Roberts^[16]^, three sub-samples were measured for each sample. For all samples, allowance was made for the effect of sample moisture content^[17]^, grain size^[18]^ and HF acid etching^[19]^ on beta-dose attenuation.

Gamma dose rates were measured directly by *in situ* gamma spectrometry to take into account any spatial heterogeneity in the gamma radiation field within ~30 cm of each sample (as gamma rays can penetrate this distance through most sediments and rocks). The gamma dose rate was measured at every sample location. Counts were collected for 1 hr with either a 1-inch or 2-inch NaI(Tl) detector. The detectors were calibrated using the concrete blocks at Oxford University^[20]^ and the gamma dose rates were determined using the ‘threshold’ technique^[21]^. This approach gives an estimate of the combined dose rate from gamma-ray emitters in the U and Th decay chains and from ^40^K.

The cosmic-ray dose rates were calculated following Prescott and Hutton^[22]^, and adjusted for geomagnetic latitude, altitude, sediment overburden and water content. In most sedimentary settings the cosmic-ray dose rate only makes up a small proportion of the total dose rate for a sample. The beta and gamma dose rates for samples from Wallen Wallen are very low, but typical for sand from the eastern seaboard of Australia^[23,24,25]^. The cosmic-ray dose rate for the uppermost eight samples makes up between ~40–50% of the total dose rate, and between 20–30% for deeper samples. Calculation of the cosmic-ray dose rate for each sample is based on the depth below current ground surface, and it assumes that sedimentation was steady over time. If there were any significant breaks in sedimentation, such as a stable surface over a long period of time, or the rate of sedimentation was much slower, then the latter assumption may not be valid, and the cosmic-ray dose rate will be underestimated. This is more likely to affect the deeper samples.

We assumed an effective internal alpha dose rate of 0.03 ± 0.01 Gy/ka. Current water contents of 2–6% were measured. We used a water content of 5 ± 1.3% for all samples and assumed this to represent the long-term water content (i.e. averaged over the entire period of sample burial) with an uncertainty sufficient to accommodate the likely range of water contents experienced by these deposits; the OSL ages increase by ~1% for each 1% increase in water content. The environmental dose rate data together with the total dose rates are provided in Table S5.

**Equivalent dose (D_e_) determination**

All single-grain quartz measurements were made using the single-aliquot regenerative-dose (SAR) procedure^[26,27]^. The SAR procedure involves measuring the OSL signals from the natural (burial) dose (L_n_) and from a series of regenerative doses (L_x_) that adequately bracket the D_e_ value (given in the laboratory by means of the calibrated ^90^Sr/^90^Y beta source). Grains were preheated at 260°C for 10 s prior to optical stimulation by an intense, green (532 nm) laser beam for 2 s at 125°C. A duplicate regenerative dose was included in the sequence to check on the adequacy of this sensitivity correction, and a ‘zero regenerative dose’ (0 Gy) measurement cycle was included to monitor the extent of any ‘recuperation’ induced by the preheat treatment. As a check on possible contamination of the acid-etched quartz grains by other mineral inclusions, we also applied the OSL IR depletion ratio test^[28]^ to each grain at the end of the SAR sequence, using an infrared exposure of 40 s at 50°C.

A total of 7,800 individual quartz grains were measured (between 400 and 1,000 grains per sample). Unsuitable grains were identified using a series of quality-assurance tests^[12]^ and grains were rejected for the following reasons:

1. Initial T_n_ signal is less than 3σ above the corresponding background count, or the relative error on T_n_ is >25%.
2. Recuperation ratio (i.e., the ratio of the L_x_/T_x_ values for the 0 Gy and maximum regenerative doses) is >5%.
3. Recycling ratio (i.e., the ratio of L_x_/T_x_ values for the duplicate regenerative doses) is not consistent with unity at 2σ.
4. OSL IR depletion ratio is more than 2σ less than unity^[28]^.
5. L_x_/T_x_ ratios are too scattered to be reliably fitted with a curve, or have a large figure-of-merit (FOM) value with an upper limit of 10%, or a have a reduced chi-square value of >5^[29,30]^.
6. D_e_ value is obtained by extrapolation of the fitted DRC, rather than interpolation among the regenerative-dose signals.
7. L_n_/T_n_ ratio is statistically consistent with, or higher than, the saturation level of the corresponding DRC, so that a finite D_e_ value and error estimate could not be obtained.

Table S4 lists the numbers of individual grains measured, rejected and accepted for D_e_ determination for each of the samples, and the reasons for grain rejection. By far the most grains (48–66%) are rejected because they simply do not emit light or emit so little light that the counting statistics are too poor to obtain meaningful information (criterion 1). All accepted grains provide reliable estimates of D_e_.

**Table S4:** Number of individual quartz grains measured, rejected and accepted for each, together with the reasons for grain rejection.

| **Sample** | **No. of grains measured** | **Rejection criteria** | | | | | | | **Sum of grains rejected** | **No. of grains accepted** | **No of ‘modern’ grains** |
| --- | --- | --- | --- | --- | --- | --- | --- | --- | --- | --- | --- |
|  |  | **1** | **2** | **3** | **4** | **5** | **6** | **7** |  |  |  |
| WWC19-1 | 1000 | 526 | 51 | 61 | 24 | 75 | 0 | 0 | 738 | 262 | 142 |
| WWC19-2 | 500 | 270 | 7 | 44 | 8 | 23 | 0 | 2 | 354 | 146 | 17 |
| WWC19-3 | 500 | 308 | 4 | 22 | 15 | 24 | 2 | 2 | 377 | 123 | 9 |
| WWC19-4 | 600 | 308 | 5 | 35 | 23 | 44 | 0 | 1 | 416 | 184 | 4 |
| WWC19-5 | 600 | 335 | 7 | 23 | 17 | 38 | 11 | 2 | 433 | 167 | 2 |
| WWC19-6 | 1000 | 656 | 13 | 29 | 15 | 57 | 3 | 2 | 775 | 225 | 2 |
| WWC19-7 | 400 | 251 | 3 | 9 | 6 | 23 | 3 | 1 | 296 | 104 | 0 |
| WWC19-12 | 500 | 274 | 8 | 47 | 14 | 47 | 1 | 2 | 393 | 107 | 0 |
| WWC19-13 | 500 | 304 | 9 | 46 | 12 | 23 | 2 | 1 | 397 | 103 | 0 |
| WWC19-11 | 500 | 251 | 9 | 47 | 8 | 57 | 3 | 0 | 375 | 125 | 0 |
| WWC19-10 | 400 | 195 | 10 | 40 | 9 | 43 | 2 | 2 | 301 | 99 | 0 |
| WWC19-9 | 500 | 258 | 2 | 39 | 20 | 51 | 11 | 8 | 391 | 109 | 0 |
| WWC19-8 | 800 | 380 | 12 | 113 | 31 | 67 | 17 | 18 | 638 | 162 | 1 |

L_n_, L_x_, T_n_ and T_x_ values were estimated from the first 0.22 s of OSL decay, with the mean count recorded over the last 0.3 s subtracted as background. Sensitivity-corrected (L_x_/T_x_) dose response curves were then constructed from the L_x_ and T_x_ OSL signals, using a general-order kinetic (GOK) function^[31]^, and the sensitivity-corrected natural OSL signal (L_n_/T_n_) was projected onto the fitted DRC to estimate the D_e_ value by interpolation. All data analyses, including curve fitting, D_e_ determination and error estimations, were achieved using the functions implemented in the R-package ‘numOSL’^[32]^.

**a.**
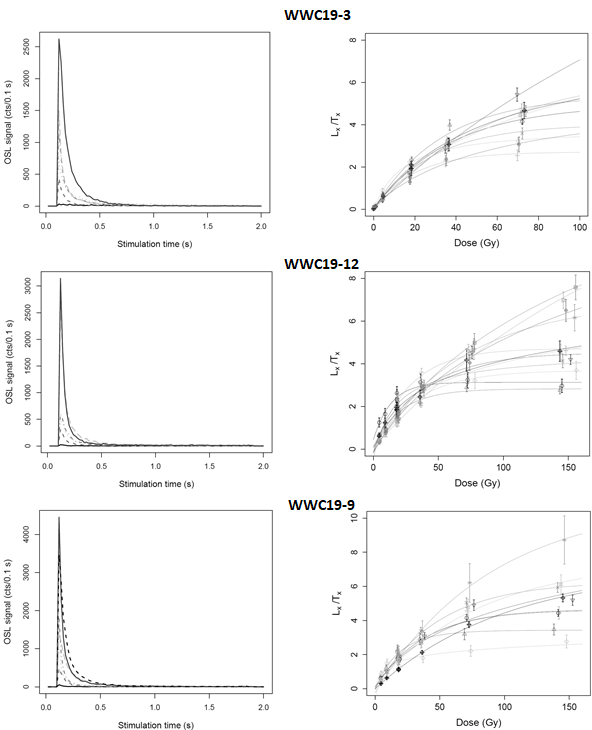


**b.**
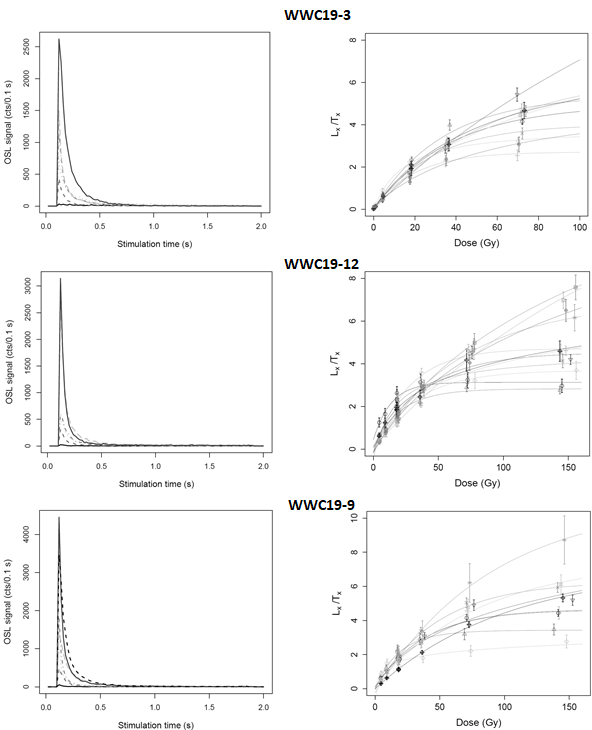


**c.**
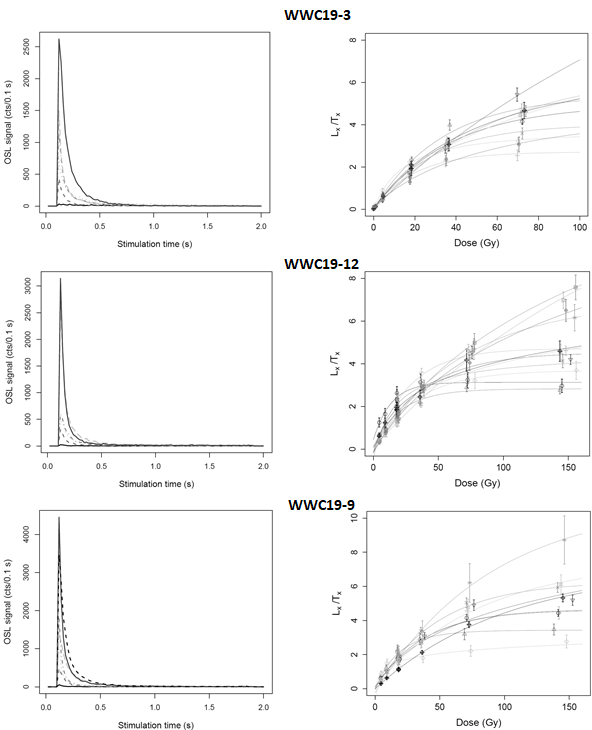


**Figure S3:** Representative OSL decay curves and their corresponding dose response curves for a selection of grains from three samples (a) WWC19-3, (b) WWC19-12 and (c) WWC19-9.

Fig. S3 shows a selection of representative OSL decay and dose response curves for one sample from the top, middle and bottom of the excavated profile (WWC19-3, WWC19-12 and WWC19-9, respectively). The OSL decay curves exhibit a range of shapes but are generally quite reproducible and decay rapidly to instrumental background, with less than ~5% of the initial signal remaining after 0.3 seconds of optical stimulation. The majority of dose response curves have very similar shapes and continue to grow with an increase in dose up to at least a dose of 50 Gy, which captures the D_e_ values for most samples, except the deepest sample.

The performance of the single-grain OSL procedure, including the rejection criteria, was tested using a dose recovery test^[26]^ on sample WWC19-10. The grains were first bleached for two days in natural sunlight and then given a beta dose of 307 s to act as a surrogate ‘natural’ dose. The sample was measured using two different PH combinations. Two hundred grains were measured using the procedure outlined above, using a preheat combination of 240°C for 10 s (PH-1) and 160°C for 5 s (PH-2) and another 300 grains were measured using a PH1 of 260°C for 10 s and a PH2 of 160°C for 5 s. Both sets of measurements resulted in a weighted mean dose recovery ratio (i.e., the ratio of measured dose to given dose) consistent with unity at 2σ; PH1 of 240°C for 10 s (0.96 ± 0.02; n=32) and PH1 of 260°C (1.01 ± 0.02; n=85). This demonstrates that the single-grain OSL procedure can produce reliable estimates of measured dose for the samples measured in this study. The dose recovery results for the two data sets are shown as radial plots in Fig. S4. Overdispersion values of 7 ± 7% and 6 ± 2% were obtained.


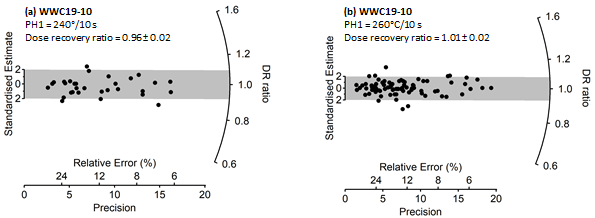


**Figure S4:** Given over measured dose values (DR ratios) for individual grains from sample WWC19-10, measured using two different PH1 temperatures. PH2 was 160°C for 5 s for both datasets. All grains consistent with the given dose fall within the grey band that is centred on a value of unity (ratio of 1).

**Single-grain equivalent dose (D_e_) results**

It is commonplace in single-grain OSL dating for there to be some spread in the data due to natural variability and other complicating factors. To quantify the degree of spread, we routinely calculate the overdispersion (OD) values for the D_e_ distributions of each sample. OD represent the relative standard deviation (i.e., the coefficient of variation) of the D_e_ distribution after accounting for the various measurement uncertainties^[33,34]^. Even for samples that have been well-bleached (zeroed by sunlight) prior to deposition and that remained undisturbed since burial, some degree of OD is present. The CAM OD values for the samples from Wallen-Wallen range between 46 ± 4 % (WWC19-9) and 83 ± 7 % (WWC19-3), with even greater values for the two samples collected closest to the modern surface. All samples have OD values that are higher than expected for samples that were well-bleached prior to deposition and remained undisturbed since burial, the primary assumptions in optical dating.

Information about potential reasons for OD can be obtained by looking at the shape and patterns of the D_e_ distributions for each sample when plotted as radial plots. The D_e_ values for all accepted grains (Table S5) are displayed as radial plots in Fig. S6, for each of the samples, except WWC19-1 and WWC19-2 for which histograms are provided. In radial plots, each point represents a single grain, for which the D_e_ can be read by extending a line from the ‘standardised estimate’ axis on the left-hand side to intersect the radial axis on the right; the point of intersection is the D_e_. The uncertainty on this estimate can be read by extending a line vertically from the data point to intersect the horizontal axis running along the bottom of the plot. This axis shows the relative standard error in % (i.e. the standard error, in Gy, divided by the D_e_ estimate, in Gy, multiplied by 100) and its reciprocal (the ‘precision’). The most precise estimates fall to the right and the least precise to the left. If these independent estimates are consistent with statistical expectations, then 95% of the points should scatter within a band of width ±2 units projecting from the left-hand (‘standardised estimate’) axis to any chosen D_e_ value on the right-hand, radial axis. The radial plot, therefore, provides simultaneous information about the spreads, precision and statistical consistency of the D_e_ values. The radial plots in Fig. S6, showing the data for the laboratory-controlled dose recovery tests, show best–case scenarios.

The two uppermost samples—WWC19-1 and WWC19-2—are both dominated by a population of grains with D_e_ values that are consistent with zero (including negative values). Log transformations are not applicable to such data sets, so the standard radial plot and statistical models cannot be used. Instead, the distributions for these two samples are shown as histograms in Fig. S5 and we applied an unlogged version of the CAM (CAMul) to WWC19-1 and an unlogged version of the minimum age model (MAMul) to WWC19-2.


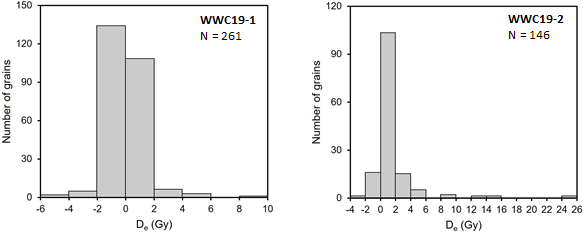


**Figure S5**: D_e_ distributions for WWC19-1 and WWC19-2 shown as histograms.

For the rest of the samples, shown in Fig. S6, two key observations that can be made from the radial plots for the samples:

1. Contamination by older grains is observed, often as discrete outliers or even a series of outliers that form a discrete dose component (e.g. WWC19-5 and WWC19-10). These are shown as open triangles in the radial plots, above the grey bars, and were excluded from calculation of the final D_e_ value for age determination. Occasional younger outliers are also observed, often associated with modern grains and these are shown as open triangles below the grey bars. The statistical outliers were identified using the normalised median absolute deviation (nMAD) method^[35,36]^. Log D_e_ values with nMADs of greater than 2.5 were excluded.
2. The remaining grains that are thought to represent the original depositional age of the sediment and shown as black filled circles in the radial plot, still show some additional spread, but is spread randomly around a central value represented by the grey bar in each of the radial plots. The latter was determined using the central age model (CAM) of Galbraith^[26]^ after rejection of the statistical outliers and ‘modern’ grains. The CAM assumes that the D_e_ values for all grains are centred on some average value of D_e_ (similar to the median) and the estimated standard error takes account of any D_e_ overdispersion; hence, the greater the OD, the larger the error.

Together, these observations suggest that there is some post-depositional mixing of the sediment likely due to bioturbation from soil fauna and flora and that the stratigraphic integrity of the sediment is to some degree compromised. Association between the age obtained for the sediment and artefacts found within the deposit is therefore not assured, and additional sources of corroborating evidence is required. The final D_e_ values determined using the approaches discussed above are provided in Table S5.


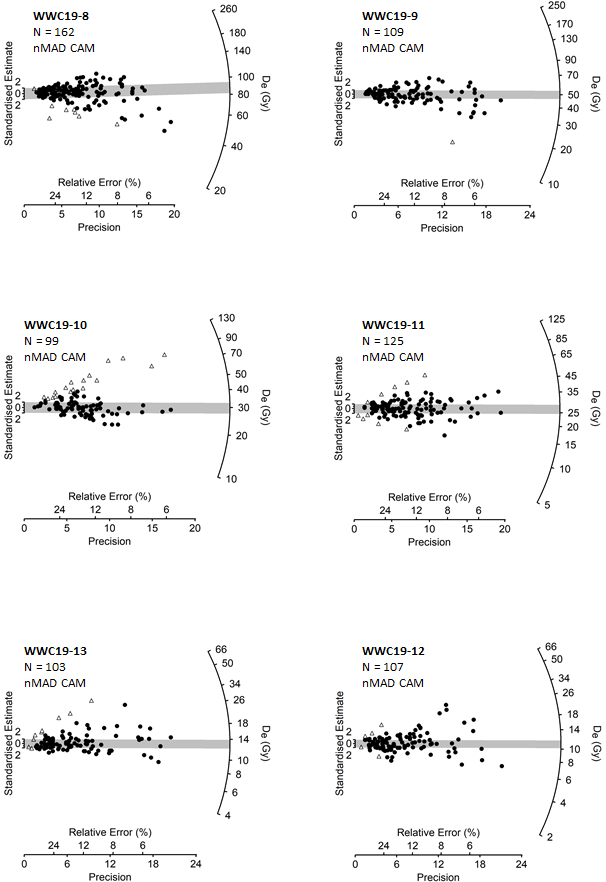


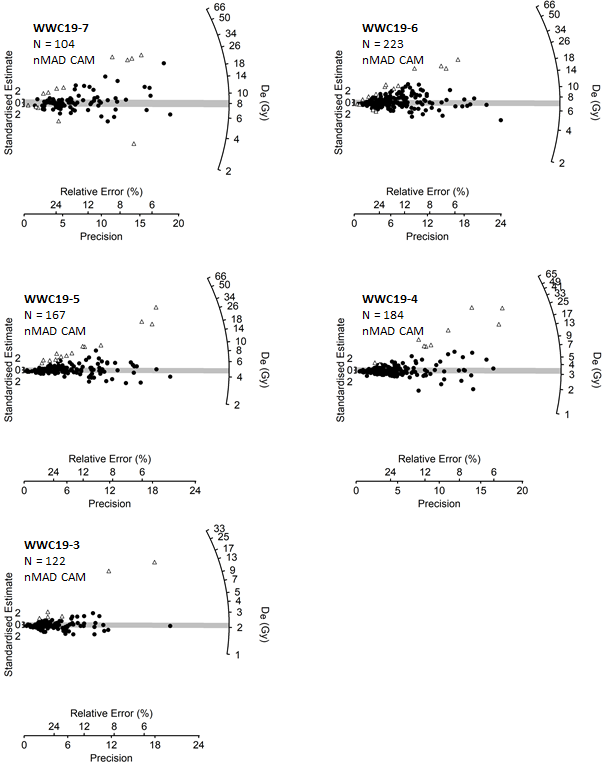


**Figure S6:** Radial plots for the thirteen samples measured in this study. Samples are presented from deepest to shallowest. The grey band is centred on the D_e_ value determined using the central age model after removal of outliers (shown as open triangles) identified using nMAD.

**Age estimates**

OSL age estimates for all samples at WWC are listed in Table S5. Uncertainties on the ages are given at 1σ (the standard error on the mean) and were estimated by combining, in quadrature, all known and estimated sources of random and systematic error.

**Table S5:** Dose rate data, equivalent dose (D_e_) and overdispersion (OD) values, and ages for WWC. Samples are listed in depth order.

| **Sample** | **Layer** | **Water (%)** | **External dose rate (Gy/ka)** | | | | **D_e_ (Gy)** | **Model** | **OD (%)** | **Optical age (ka)** |
| --- | --- | --- | --- | --- | --- | --- | --- | --- | --- | --- |
|  |  |  | **Beta** | **Gamma** | **Cosmic** | **Total** |  |  |  |  |
| WWC19-1 | 1 | 5 ± 1 (6.0) |  |  |  |  | -0.01 ± 0.03 | CAMul |  | modern |
| WWC19-2 | 1 | 5 ± 1 (5.4) | 0.10 ± 0.02 | 0.12 ± 0.01 | 0.188 | 0.44 ± 0.04 | 0.34 ± 0.03 | MAMul | 173 ± 28 | 0.77 ± 0.09 |
| WWC19-3 | 1 | 5 ± 1 (2.5) | 0.09 ± 0.02 | 0.12 ± 0.01 | 0.185 | 0.44 ± 0.04 | 2.17 ± 0.11 | nMAD CAM | 72 ± 6 (39) | 5.0 ± 0.5 |
| WWC19-4 | 2 | 5 ± 1 (2.3) | 0.10 ± 0.02 | 0.11 ± 0.01 | 0.183 | 0.43 ± 0.03 | 3.43 ± 0.16 | nMAD CAM | 76 ± 5 (49) | 8.0 ± 0.8 |
| WWC19-5 | 2 | 5 ± 1 (6.6) | 0.08 ± 0.01 | 0.10 ± 0.01 | 0.181 | 0.39 ± 0.03 | 4.84 ± 0.20 | nMAD CAM | 75 ± 5 (43) | 12.4 ± 1.2 |
| WWC19-6 | 2 | 5 ± 1 (2.7) | 0.06 ± 0.01 | 0.09 ± 0.01 | 0.179 | 0.35 ± 0.03 | 7.12 ± 0.29 | nMAD CAM | 67 ± 4 (53) | 20.2 ± 2.1 |
| WWC19-7 | 2 | 5 ± 1 (3.5) | 0.06 ± 0.01 | 0.08 ± 0.01 | 0.177 | 0.36 ± 0.03 | 8.16 ± 0.48 | nMAD CAM | 76 ± 6 (53) | 23.0 ± 2.4 |
| WWC19-12 | 3 | 5 ± 1 (1.5) | 0.08 ± 0.01 | 0.12 ± 0.01 | 0.169 | 0.40 ± 0.03 | 11.0 ± 0.68 | nMAD CAM | 68 ± 5 (59) | 27.9 ± 2.9 |
| WWC19-13 | 3 | 5 ± 1 (2.4) | 0.09 ± 0.01 | 0.15 ± 0.01 | 0.165 | 0.43 ± 0.03 | 13.0 ± 0.66 | nMAD CAM | 66 ± 5 (44) | 30.3 ± 2.7 |
| WWC19-11 | 4 | 5 ± 1 (4.4) | 0.16 ± 0.02 | 0.20 ± 0.01 | 0.163 | 0.55 ± 0.03 | 26.8 ± 1.15 | nMAD CAM | 57 ± 4 (42) | 49.0 ± 3.7 |
| WWC19-10 | 4 | 5 ± 1 (4.0) | 0.18 ± 0.02 | 0.24 ± 0.01 | 0.162 | 0.61 ± 0.03 | 30.3 ± 1.36 | nMAD CAM | 60 ± 5 (36) | 49.6 ± 3.6 |
| WWC19-9 | 4 | 5 ± 1 (3.8) | 0.25 ± 0.02 | 0.26 ± 0.01 | 0.159 | 0.70 ± 0.03 | 50.8 ± 2.1 | nMAD CAM | 44 ± 4 (37) | 72.8 ± 4.8 |
| WWC19-8 | 5 | 5 ± 1 (1.5) | 0.12 ± 0.02 | 0.17 ± 0.01 | 0.155 | 0.48 ± 0.03 | 86.8 ± 2.9 | nMAD CAM | 48 ± 3 (35) | 182.1 ± 13.6 |

**4. Middle Canalpin Creek Radiocarbon Chronology**

AMS radiocarbon dating was undertaken on a total of four samples from 15 cm, 110 cm, 250 cm and 280 cm below surface at Middle Canalpin Creek. Samples were recorded in-situ and collected in plastic vials. Samples were obtained from four quadrats of the excavation. The sample from 15 cm consisted of a *Trichomya* *hirsute* shell, while all other samples were charred organic remains interpreted as charcoal potentially from anthropogenic burning. Charred remains were not identified to species level prior to radiocarbon analyses.

Samples were analysed using the AMS radiocarbon technique at the Australian National University Radiocarbon Laboratory, Research School of Earth Sciences. δ^13^C values were used to correct for fractionation. All samples were calibrated to 2 sigma using the Oxcal radiocarbon online calibration tool. The Hogg^[3]^ Southern Hemisphere calibration curve was utilised for the MCA20 sequence. Results are shown in Table S6.

The upper samples show good consistency with the OSL results from MCA20. However, the lower samples at 250 cm and 280 cm deviate from the OSL ages (Table S8). This was a feature noted in the original Neal and Stock manuscript^[1]^ where ^14^C results demonstrated an inversion of age estimates below 200 cm depth. Neal and Stock interpreted these results to be associated with modern carbon in ground water contaminating the samples^[1]^. Here we see similar results indicating that similar processes may have taken place throughout the island.

**Table S6:** Radiocarbon results for samples collected from MCA20.

| **Laboratory code** | **Depth** | **Material^a^** | **F^14^C** | **^14^C age** | **Calibrated ^14^C age (cal yrs BP)^b^** | |
| --- | --- | --- | --- | --- | --- | --- |
|  | **(cm)** |  | **(%)** | **(years BP)** | **68.3% probability** | **95.4% probability** |
| S-ANU-69133 | 15 | Shell (*Trichomya* *hirsuta*) | 0.9165 ± 0.0023 | 696 ± 21 | 405–245 | 455–160 |
| S-ANU-68033 | 110 | Charcoal | 0.2734 ± 0.0010 | 10,418 ± 35 | 12,465–12,095 | 12,475–12,020 |
| S-ANU-68036 | 250 | Charcoal | 0.0102 ± 0.0005 | 36,829 ± 430 | 41,960–41,380 | 42,170–41,105 |
| S-ANU-68035 | 280 | Charcoal | 0.0055 ± 0.0005 | 41,794 ± 796 | 45,340–44,015 | 45,920–43,200 |

^a^Shell was pretreated using an acid etch, whereas charcoal was pretreated using a acid-base-acid (ABA) procedure.

^b^ All radiocarbon ages obtained for charcoal samples were calibrated using the SH20 calibration curve^[3]^. The shell sample was calibrated using the Marine20 calibration curve^[4]^ and a ΔR value of -156 ± 18 based on 10 datapoints located closest to MCA20, sourced from calib.org/marine/. All age ranges are rounded to the nearest 5 years.

**5. MCA20 Luminescence Chronology**

## **OSL Sample Preparation**

Sample preparation was designed to isolate pure extracts of 180-212 µm light-safe quartz grains following standard procedures^[6]^. Treatments were applied to remove contaminant carbonates, feldspars, organics, heavy minerals and acid soluble fluorides. The outer ~10 µm alpha-irradiated rind of each grain was removed by double etching in 48 % hydrofluoric acid for 40 min. Quartz grains were loaded on to custom-made aluminum discs drilled with a 10 x 10 array of chambers, each of 300 µm depth and 300 µm diameter^[37]^. Burial doses (D_b_) were determined from OSL measurements made on 1000 individual quartz grains on a Risø TL/OSL DA-20 reader using a green (532 nm) laser for optical stimulation, and the ultraviolet emissions were detected by an Electron Tubes Ltd 9235QA photomultiplier tube fitted with 7.5 mm Hoya U-340 filter. Laboratory irradiations were conducted using a calibrated ^90^Sr/^90^Y beta source mounted on the reader.

Lithogenic radionuclide activity concentrations of material extracted from sampling tubes were determined using high-resolution gamma spectrometry^[38]^. Samples were counted for 3-7 days on an Ortec HPGe co-axial gamma detector. Dose rates were calculated using the conversion factors of Liritzis^[39]^ with β-attenuation factors taken from^[40]^. Cosmic dose rates were calculated from Prescott and Hutton^[22]^. Burial doses were calculated using age modelling techniques of Galbraith and co-workers^[26,41,42]^.

**Analysis**

Equivalent doses (D_e_) were determined using a modified single-aliquot-regenerative dose (SAR) protocol^[43]^. A dose-response curve was constructed for each grain. OSL signals were measured for 1 s at 125 °C (laser at 90% power) using a preheat of 240 °C (held for 10 s) for the ‘natural’ and regenerative doses, and a pre-heat of 160 °C (held for 10 s) for the test doses (1-10 Gy). The OSL signal was determined from the initial 0.1 s of data, using the final 0.2 s to estimate the background count rate. Each disc was exposed to infrared (IR) radiation for 40 s at 125 °C prior to measurement of the OSL signal to bleach any IR-sensitive signal.

Grains were selected based on criteria from Pietsch^[44]^ and Pietsch^[45]^, i.e. grains were rejected if they did not produce a measurable OSL signal in response to the 1-10 Gy test dose, had OSL decay curves that did not reach background after 1 s of laser stimulation, produced natural OSL signals that did not intercept the regenerated dose-response curves, or had unacceptable sensitivity changes throughout the measurement cycle i.e. they were rejected if either of the second or third Test Dose signals varied in sensitivity from the first Test Dose (associated with the Natural Dose) by more than 30 %. The sensitivity rule was relaxed for GU72.1, which displayed poor luminescence characteristics. No outliers were excluded from the data set.

**Results**

All samples had low concentrations of radionuclides, with no clear evidence for disequilibrium in the U-238 decay chain within the limits of measurement uncertainties (Table S7). Following measurement of sample water content, a long-term water content of 5 ± 2.5 % was used for all samples. The estimated dose rates were very low, ranging from 0.29±0.02 Gy/ka to 0.39±0.04 Gy/ka. Australian quartz sediment has a bright luminescence signal with a high recovery, i.e. a large proportion of analysed grains yield a consistent OSL signal^[46]^, and recoveries were excellent for all samples. Burial doses were modelled using the Central Age Model^[26]^ with an applied overdispersion parameter of 15%. Equivalent doses obtained from OSL analysis of single grains of sand yield additional information on likely rates of disturbance to the host sands from burrowing animals, humans and other disturbance factors. At MCA20 overdispersion (i.e. the degree of spread in the data beyond that which can be explained by measurement uncertainties) ranges from 28% to 69% (Fig. S7, Fig. S8). This is higher than global average of 20 ± 1%^[47]^, but in Pleistocene-age sediments with low dose rates, additional scatter often arises from rare, high-dose grains such as zircon^[48]^, or incomplete zeroing of sediments prior to deposition. In this context, the OSL results are relatively well-defined and are consistent with stratigraphic depth, suggesting vertical mixing has been minor (Table S8).

**Table S7:** Radionuclide activities, water contents and estimated dose rates for MCA20

|  | **^238^U** | **^226^Ra** | **^210^Pb** | **^232^Th** | **^40^K** | **^137^Cs** | **Water** | **Dose Rate** |
| --- | --- | --- | --- | --- | --- | --- | --- | --- |
| **Lab code** | **Bq/kg** | **Bq/kg** | **Bq/kg** | **Bq/kg** | **Bq/kg** | **Gy/ka** | **% dry wt** | **Gy/ka** |
| GU72.1 | 3.5±0.2 | 4.2±0.1 | 3.9±0.3 | 2.3±0.1 | 12.4±0.3 | -0.02±0.02 | 2.6 | 0.35±0.03 |
| GU72.2 | 4.8±1.0 | 3.6±0.2 | 4.3±1.0 | 2.2±0.3 | 11.9±1.3 | 0.04±0.08 | 3.0 | 0.35±0.03 |
| GU72.3 | 2.3±0.3 | 2.9±0.1 | 3.3±0.4 | 1.2±0.1 | 10.8±0.5 | -0.01±0.03 | 2.5 | 0.29±0.03 |
| GU72.4 | 9.0±1.0 | 9.8±0.3 | 10.3±1.0 | 2.9±0.4 | 13.8±1.1 | 0.07±0.07 | 3.6 | 0.39±0.04 |
| GU72.5 | 2.0±0.6 | 3.4±0.1 | 3.8±0.6 | 1.4±0.3 | 10.1±0.9 | 0.00±0.06 | 61 | 0.29±0.03 |
| GU72.6 | 3.2±0.3 | 3.4±0.1 | 2.8±0.3 | 1.6±0.3 | 9.1±0.4 | -0.02±0.03 | 61 | 0.28±0.02 |
| GU72.7 | 2.9±0.3 | 3.6±0.1 | 3.8±0.4 | 1.6±0.2 | 9.2±0.4 | -0.01±0.03 | 59 | 0.28±0.02 |
| GU72.8 | 4.0±0.4 | 3.5±0.1 | 4.0±0.4 | 1.4±0.3 | 9.3±0.5 | 0.02±0.04 | 3.2 | 0.29±0.03 |
| GU72.9 | 3.3±0.3 | 3.9±0.1 | 4.0±0.3 | 1.7±0.2 | 9.0±0.4 | 0.02±0.02 | 3.5 | 0.28±0.02 |

**Table S8:** MCA20 OSL results and ages

| **Lab No** | **Sample** | **n** | **Recovery** | ****_d_** | **D_e_**  **CAM** | **D_e_**  **MAM** | **Age**  **CAM** |
| --- | --- | --- | --- | --- | --- | --- | --- |
|  |  |  | **(%)** | **(%)** | **(Gy)** | **(Gy)** | **(ka)** |
| GU72.1 | MCA20-40 | 26 | 3 | 42 | 0.78±0.10 | 0.54±0.13 | 2.24±0.30 |
| GU72.2 | MCA20-67 | 112 | 13 | 69 | 2.17±0.16 | 1.42±0.09 | 6.2±0.7 |
| GU72.3 | MCA20-121 | 59 | 6 | 57 | 5.2±0.4 | 4.0±0.5 | 17.7±2.0 |
| GU72.4 | MCA1-1 585 | 75 | 8 | 28 | 59±3 | 52±5 | 151±15 |
| GU72.5 | MCA20-190cm | 69 | 8 | 40 | 10.3±0.6 | 7.9±0.7 | 36±4 |
| GU72.6 | MCA20-205cm | 97 | 10 | 45 | 10.4±0.5 | 7.6±0.8 | 38±4 |
| GU72.7 | MCA20-240 | 72 | 8 | 46 | 14.3±0.9 | 8.0±0.8 | 51±5 |
| GU72.8 | MCA20-225 | 83 | 8 | 32 | 11.9±0.5 | 8.6±0.8 | 41±4 |
| GU72.9 | MCA20-300 | 96 | 10 | 38 | 21.3±1.0 | 13.6±1.3 | 77±7 |


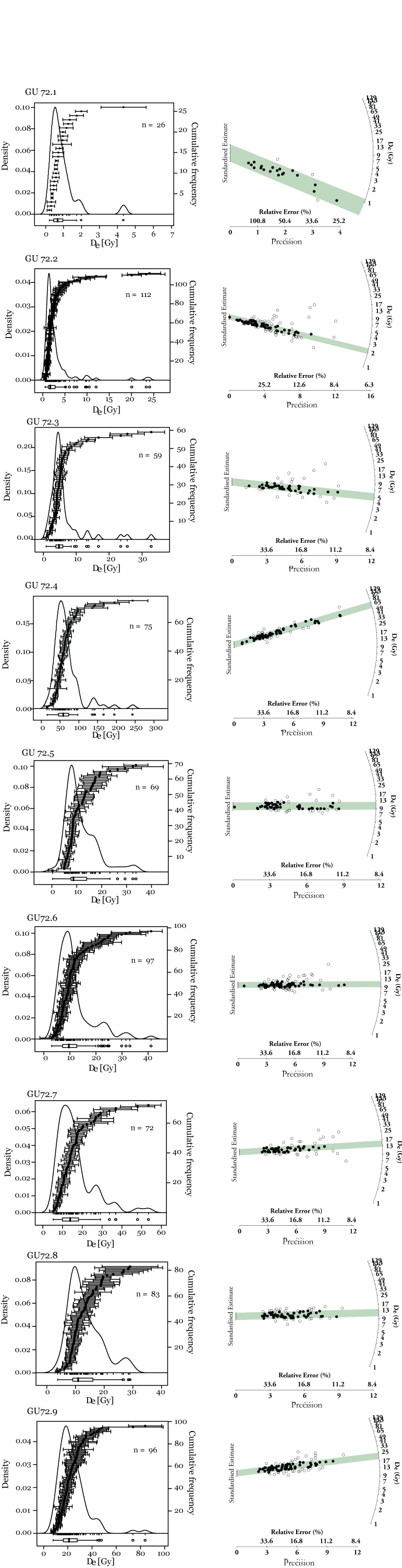


**Figure S7:** Kernel density functions and radial plots for all OSL samples from MCA20.


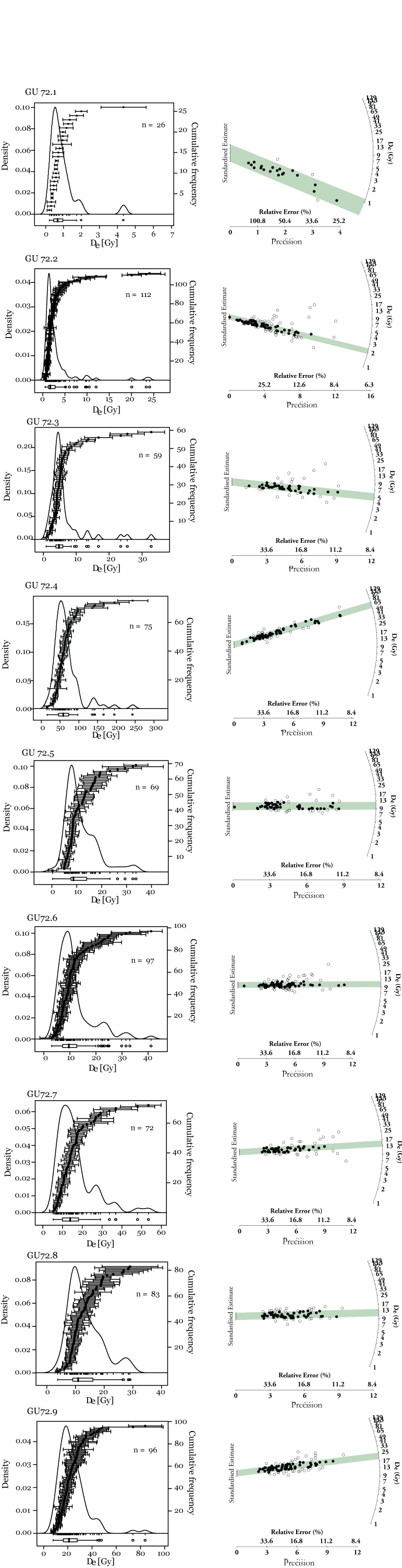


**Figure S8:** Individual D_e_ values for MCA20 are displayed as a kernel density function (left) with the ranked individual D_e_ values plotted cumulatively (closed circles). At right, the same data for each sample is shown as a radial plot. Shaded areas on the radial plot show the D_b_ (burial doses) measured using the Central Age Model (CAM) with 2σ errors.

**6. WWC Lithics**

**Table S9**: Artefact counts and density for WWC.

| Spit | Depth (m) | Banded Red Hydrothermal Chert | Chert | Silcrete | Banded Rhyolite | Quartz | Crystal Quartz | Quartzite | Ground Volcanic | Ground Basalt | Basalt | Ground Sandstone | Green Volcanic | Volcanic | Ground Ochre | Retouched | Core | Bipolar | Axe Flake | Yellow Ochre | Total Artefacts | Area Excavated m^2^ | Heated Silcrete | Heated Silcrete/m^2^ | Artefacts/m2 |
| --- | --- | --- | --- | --- | --- | --- | --- | --- | --- | --- | --- | --- | --- | --- | --- | --- | --- | --- | --- | --- | --- | --- | --- | --- | --- |
| 1 | 0.04 |  | 4 |  |  | 1 |  | 1 |  |  |  |  |  |  |  |  |  |  |  |  | 6 | 24 | 0 | 0.00 | 0.25 |
| 2 | 0.07 |  | 8 | 3 |  | 5 |  | 3 |  |  |  |  |  |  |  |  |  |  |  |  | 19 | 24 | 0 | 0.00 | 0.79 |
| 3 | 0.11 |  | 4 | 7 |  | 3 |  | 4 |  |  |  |  |  |  |  |  | 1 |  |  |  | 18 | 24 | 0 | 0.00 | 0.75 |
| 4 | 0.14 |  | 4 | 8 |  | 9 | 1 | 4 |  |  |  |  |  |  |  |  |  |  |  |  | 26 | 24 | 0 | 0.00 | 1.08 |
| 5 | 0.18 |  | 11 | 4 |  | 11 |  | 2 |  |  | 1 |  | 1 | 1 |  |  |  |  |  |  | 31 | 24 | 0 | 0.00 | 1.25 |
| 6 | 0.21 |  | 19 | 7 |  | 7 |  | 4 |  |  |  |  |  |  |  |  |  |  |  |  | 37 | 24 | 2 | 0.08 | 1.54 |
| 7 | 0.25 |  | 26 | 4 |  | 13 |  | 12 |  |  | 1 | 1 |  | 1 |  |  |  | 2 |  |  | 58 | 24 | 2 | 0.08 | 2.42 |
| 8 | 0.27 |  | 18 | 15 |  | 8 | 1 | 6 |  |  | 1 |  |  | 1 |  |  |  |  |  |  | 50 | 24 | 1 | 0.04 | 2.04 |
| 9 | 0.30 |  | 21 | 20 |  | 9 | 1 | 5 |  |  | 2 |  |  | 2 |  |  |  |  |  |  | 60 | 24 | 5 | 0.21 | 2.42 |
| 10 | 0.32 | 1 | 41 | 39 |  | 12 | 1 | 3 |  |  |  |  |  |  |  | 1 |  |  |  |  | 97 | 24 | 6 | 0.25 | 4.00 |
| 11 | 0.35 |  | 47 | 40 |  | 18 | 2 | 2 |  | 1 | 5 |  |  | 6 |  | 1 |  |  | 1 |  | 121 | 24 | 2 | 0.08 | 4.79 |
| 12 | 0.37 |  | 13 | 21 | 1 | 8 |  | 5 |  |  | 1 |  |  | 1 |  |  |  |  |  |  | 50 | 24 | 7 | 0.29 | 2.04 |
| 13 | 0.41 |  | 24 | 31 |  | 22 | 1 | 3 |  |  |  |  | 1 |  |  |  |  |  |  |  | 82 | 24 | 3 | 0.13 | 3.42 |
| 14 | 0.45 |  | 14 | 31 |  | 17 | 1 | 13 |  |  | 1 |  |  | 1 |  | 2 |  |  |  |  | 78 | 24 | 2 | 0.08 | 3.21 |
| 15 | 0.48 |  | 21 | 33 |  | 39 | 1 | 3 |  |  |  | 1 | 1 |  |  |  |  | 1 |  |  | 99 | 24 | 4 | 0.17 | 4.17 |
| 16 | 0.52 |  | 9 | 27 | 1 | 54 | 2 | 5 |  | 1 | 1 |  | 2 | 2 |  |  | 1 |  | 2 |  | 104 | 24 | 0 | 0.00 | 4.25 |
| 17 | 0.56 |  | 17 | 26 |  | 51 | 1 | 1 | 1 |  |  |  |  | 1 |  |  |  | 1 |  |  | 98 | 20 | 0 | 0.00 | 4.85 |
| 18 | 0.59 |  | 17 | 7 |  | 41 |  | 1 | 1 |  |  | 1 |  | 1 |  |  |  |  |  |  | 69 | 18 | 3 | 0.17 | 3.78 |
| 19 | 0.61 |  | 20 | 7 |  | 29 | 2 | 3 | 1 |  |  |  | 1 | 1 |  |  | 1 |  |  |  | 64 | 18 | 1 | 0.06 | 3.56 |
| 20 | 0.64 |  | 18 | 4 |  | 10 | 1 | 1 |  |  |  |  |  |  |  |  |  |  |  | 1 | 34 | 18 | 0 | 0.00 | 1.89 |
| 21 | 0.66 | 2 | 16 | 4 |  | 12 | 3 |  |  |  |  |  |  |  |  | 1 |  |  |  |  | 37 | 18 | 0 | 0.00 | 1.94 |
| 22 | 0.69 |  | 10 |  |  | 12 | 4 |  |  |  |  |  | 1 |  |  |  |  |  |  |  | 27 | 18 | 0 | 0.00 | 1.50 |
| 23 | 0.71 |  | 16 | 2 |  | 8 |  |  |  |  |  | 3 |  |  |  |  | 1 |  |  |  | 29 | 18 | 0 | 0.00 | 1.61 |
| 24 | 0.74 |  | 19 | 4 |  | 6 |  |  |  |  |  |  |  |  | 2 |  |  |  |  |  | 31 | 18 | 0 | 0.00 | 1.78 |
| 25 | 0.77 |  | 2 |  |  | 1 |  |  |  |  |  |  |  |  | 2 |  |  |  |  |  | 5 | 18 | 0 | 0.00 | 0.28 |
| 26 | 0.81 |  | 5 | 2 |  | 3 |  |  |  |  |  |  |  |  | 3 |  |  |  |  |  | 13 | 18 | 0 | 0.00 | 0.72 |
| 27 | 0.84 |  | 5 | 7 |  | 4 |  |  |  |  |  |  |  |  |  |  |  |  |  |  | 16 | 18 | 0 | 0.00 | 0.89 |
| 28 | 0.87 |  | 10 | 3 |  | 7 |  |  |  |  |  |  |  |  | 2 |  |  |  |  | 1 | 22 | 18 | 0 | 0.00 | 1.22 |
| 29 | 0.90 |  |  |  |  | 1 |  |  |  |  |  |  |  |  |  |  |  |  |  |  | 1 | 18 | 0 | 0.00 | 0.06 |
| 30 | 0.94 |  | 3 | 3 |  | 8 |  |  |  |  |  |  |  |  |  |  |  |  |  |  | 14 | 18 | 0 | 0.00 | 0.78 |
| 31 | 0.97 |  | 8 | 3 |  | 2 |  |  |  |  |  |  |  |  |  |  |  |  |  |  | 13 | 18 | 0 | 0.00 | 0.72 |
| 32 | 1.00 |  | 1 | 1 |  | 2 |  |  |  |  |  |  |  |  |  |  |  |  |  |  | 4 | 18 | 0 | 0.00 | 0.22 |
| 33 | 1.03 |  | 1 | 3 |  |  |  |  |  |  |  |  |  |  |  |  |  |  |  |  | 4 | 8 | 0 | 0.00 | 0.50 |
| 34 | 1.06 |  | 1 |  |  |  |  |  |  |  |  |  |  |  |  |  |  |  |  |  | 1 | 8 | 0 | 0.00 | 0.13 |
| 35 | 1.09 |  |  | 2 |  |  |  |  |  |  |  |  |  |  |  |  |  |  |  |  | 2 | 8 | 0 | 0.00 | 0.25 |
| 36 | 1.12 |  | 2 | 1 |  | 1 |  |  | 1 |  |  |  |  | 1 |  |  |  |  |  |  | 6 | 8 | 0 | 0.00 | 0.63 |
| 37 | 1.15 |  |  | 3 |  |  |  |  |  |  |  |  |  |  |  |  |  |  |  |  | 3 | 8 | 0 | 0.00 | 0.50 |
| 38 | 1.18 |  | 1 | 2 |  |  |  |  |  |  |  |  |  |  |  |  |  |  |  |  | 3 | 8 | 0 | 0.00 | 0.38 |
| 39 | 1.23 |  | 1 | 4 |  | 1 |  |  |  |  |  |  |  |  |  |  |  |  |  |  | 6 | 8 | 2 | 0.25 | 0.75 |
| 40 | 1.28 |  | 3 | 5 |  |  |  |  |  |  |  |  |  |  |  |  |  |  |  |  | 8 | 8 | 0 | 0.00 | 1.13 |
| 41 | 1.33 |  | 4 | 1 |  |  |  |  |  |  |  |  |  |  | 1 |  |  |  |  |  | 6 | 8 | 0 | 0.00 | 0.88 |
| 42 | 1.38 |  | 3 | 5 |  |  |  |  |  |  |  |  |  |  |  |  |  |  |  |  | 8 | 8 | 0 | 0.00 | 1.00 |
| 43 | 1.43 |  | 4 | 1 |  |  |  |  |  |  |  |  |  |  |  |  |  |  |  |  | 5 | 8 | 0 | 0.00 | 0.63 |
| 44 | 1.50 |  | 2 |  |  | 1 |  |  |  |  |  |  |  |  |  |  |  |  |  |  | 3 | 8 | 0 | 0.00 | 0.38 |
| 45 | 1.57 |  | 3 | 4 |  | 6 |  |  |  |  |  |  |  |  |  |  |  |  |  |  | 13 | 8 | 0 | 0.00 | 1.63 |
| 47 | 1.64 |  | 3 | 3 |  |  |  |  |  |  |  |  |  |  |  |  |  |  |  |  | 6 | 8 | 0 | 0.00 | 0.75 |
| 48 | 1.69 |  |  | 3 |  |  |  |  |  |  |  |  |  |  |  |  |  |  |  |  | 3 | 8 | 0 | 0.00 | 0.38 |
| 49 | 1.74 |  | 2 | 1 |  | 2 |  |  |  |  |  |  |  |  |  |  |  |  |  |  | 5 | 8 | 0 | 0.00 | 0.63 |
| 50 | 1.79 |  | 7 | 1 |  |  |  |  |  |  |  |  |  |  |  |  |  |  |  |  | 8 | 8 | 0 | 0.00 | 1.00 |
| 51 | 1.86 |  | 10 | 1 |  |  |  |  |  |  |  |  |  |  |  |  |  |  |  |  | 11 | 8 | 0 | 0.00 | 1.38 |
| 52 | 1.93 |  | 8 | 2 |  |  |  |  |  |  |  |  |  |  |  |  |  |  |  |  | 10 | 8 | 0 | 0.00 | 1.25 |
| 53 | 2.00 |  | 7 | 3 |  |  |  |  |  |  |  |  |  |  |  | 1 |  |  |  |  | 10 | 8 | 2 | 0.25 | 1.25 |
| 54 | 2.02 |  | 8 | 4 |  |  |  |  |  |  |  |  |  |  |  |  |  |  |  |  | 12 | 8 | 0 | 0.00 | 1.50 |
| 55 | 2.05 | 1 | 13 | 5 |  |  |  |  |  |  |  |  |  |  |  |  |  |  |  |  | 19 | 8 | 0 | 0.00 | 2.38 |
| 56 | 2.07 | 1 | 10 | 5 |  |  |  |  |  |  |  |  |  |  |  | 2 | 1 |  |  |  | 16 | 8 | 0 | 0.00 | 2.00 |
| 57 | 2.11 | 1 | 19 | 16 |  |  |  |  |  |  |  |  |  |  |  | 1 |  |  |  |  | 36 | 8 | 0 | 0.00 | 4.50 |
| 58 | 2.16 | 1 | 12 | 11 |  |  |  |  |  |  |  |  |  |  |  |  |  |  |  |  | 24 | 8 | 0 | 0.00 | 3.00 |
| 59 | 2.20 |  | 12 | 3 |  |  |  |  |  |  |  |  |  |  |  | 1 |  |  |  |  | 15 | 8 | 0 | 0.00 | 1.88 |
| 60 | 2.25 |  | 3 | 1 |  |  |  |  |  |  |  |  |  |  |  |  |  |  |  |  | 4 | 2 | 0 | 0.00 | 2.00 |
| 61 | 2.29 |  |  | 2 |  |  |  |  |  |  |  |  |  |  |  | 1 |  |  |  |  | 2 | 2 | 0 | 0.00 | 1.00 |
| 62 | 2.34 |  | 1 | 1 |  |  |  |  |  |  |  |  |  |  |  |  |  |  |  |  | 2 | 2 | 0 | 0.00 | 1.00 |
| 63 | 2.38 |  |  |  |  |  |  |  |  |  |  |  |  |  |  |  |  |  |  |  | 0 | 2 | 0 | 0.00 | 0.00 |
| 64 | 2.44 |  |  |  |  |  |  |  |  |  |  |  |  |  |  |  |  |  |  |  | 0 | 2 | 0 | 0.00 | 0.00 |
| 65 | 2.50 |  | 1 |  |  |  |  |  |  |  |  |  |  |  |  |  |  |  |  |  | 1 | 2 | 0 | 0.00 | 0.50 |
| 66 | 2.56 |  |  |  |  |  |  |  |  |  |  |  |  |  |  |  |  |  |  |  | 0 | 0 | 0 | 0.00 | 0.00 |
| 70 | 2.81 |  |  |  |  |  |  |  |  |  |  |  |  |  |  |  |  |  |  |  | 0 | 0 | 0 | 0.00 | 0.00 |
| 75 | 3.10 |  |  |  |  |  |  |  |  |  |  |  |  |  |  |  |  |  |  |  | 0 | 0 | 0 | 0.00 | 0.00 |
| Total | | 7 | 592 | 456 | 2 | 444 | 22 | 81 | 4 | 2 | 13 | 6 | 7 | 19 | 10 | 11 | 5 | 4 | 3 | 2 | 1665 |  |  |  |  |


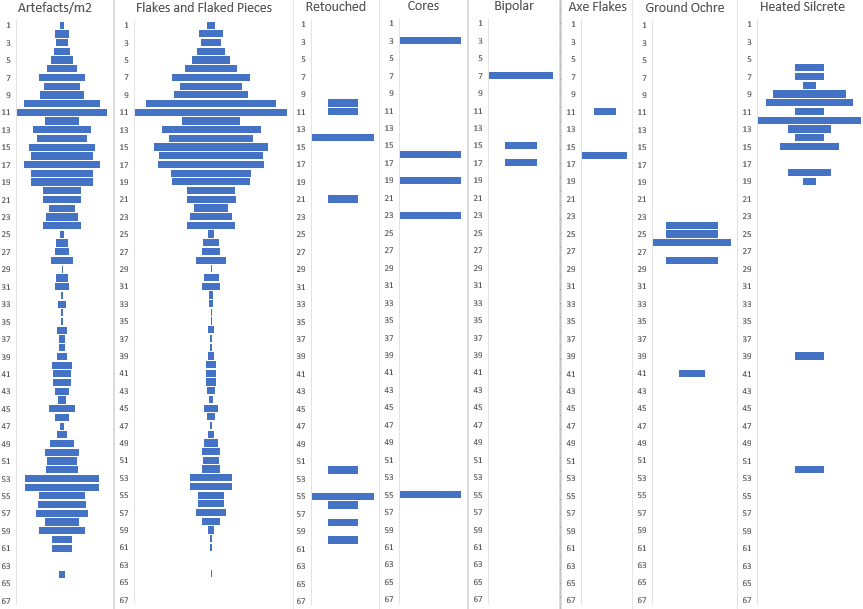


**Figure S9:** Changes in stone type counts by spit at WWC.


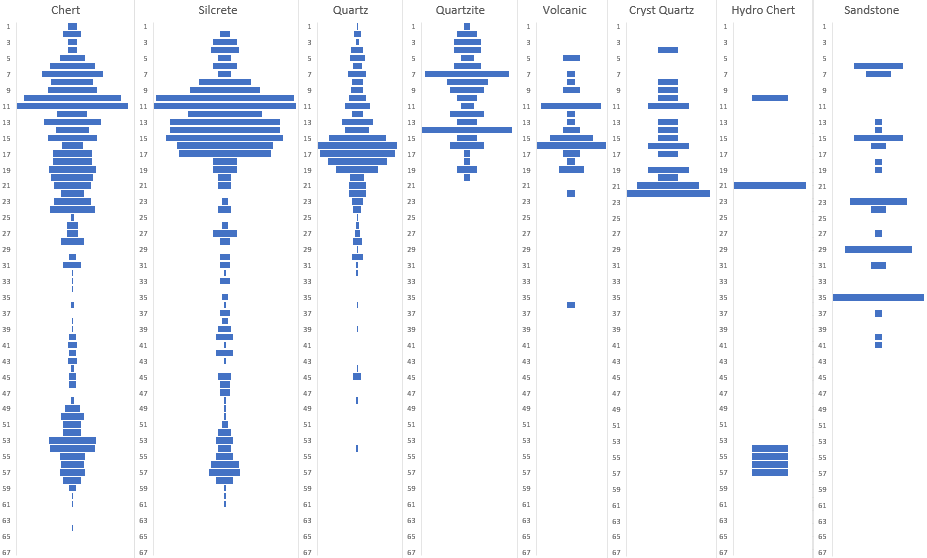


**Figure S10:** Changes in raw material counts by spit at WWC.

**7. MCA20 Lithics**

**Vertical Distribution**

Four distinct peaks in artefact discard are observed at MCA20, with two larger upper peaks in Spits 3 and 6, and two smaller lower peaks in Spits 12 and 20 (Figure S11). Two pronounced peaks in ironstone nodules are also seen, one in Spit 6 and one in Spit 11, but ironstone is largely absent below Spit 14. Whether this reflects changing cultural practices of collection for use as heat retainer or other purposes, and/or changes in local geomorphology (such as weathering and exposure of ironstone nodules through sea level rise and wave action) remains to be determined. Some of the ironstone nodules appear to have been ground (Spits 1-5) and were possibly used as red pigment. Yellow ochre (some potentially ground) also occurs in Spits 11 and 12 and may have been ground for pigment.

**Figure S11:** Vertical distribution of stone artefacts and ironstone nodules at MCA20 by spit.

Artefact types also show differing frequencies through time (Table S10). Figure S12 shows changes in the dominant artefact types at MCA20 over the 26 spits. Flakes and flaked pieces make up 96% of the assemblage and occur to the base of the excavation. Retouched flakes make up only 1.1% of the assemblage and occur between spits 5 and 26 but do not occur in the uppermost peak in artefact discard above Spit 5. Cores show a similar distribution, but do not occur below Spit 22, likely due to small sample size. Redirecting flakes are most common in above Spit 11 and peak in Spit 4. Bipolar flakes are most commonly above Spit 8. Axe flakes also occur in the top 6 spits where sample size is largest, but this may also reflect the mid to late Holocene introduction for ground axes in southern Australia. Similarly, the single backed artefact in the site is found in Spit 5. Ground ochre occur above Spit 5 and is most common in Spit 3. Yellow ochre is found in a small pulse in Spits 11 and 12. Overall, greatest technological diversity occurs in the upper deposit above and including Spit 9. This may be cultural, reflecting a greater range of activities on site including flake and axe manufacture and use, but also at least partly reflects sample size, with diversity strongly correlated to sample size (r^2^ = 0.752)

**Table S10:** Vertical distribution of major artefact types in the assemblage.

| **Spit** | **Depth cm** | **Total** | **Total Artefact Mass g** | **Mean Mass g** | **Ironstone** | **Flakes and**  **Flaked Pieces** | **Heat Break** | **Retouched** | **Redirecting** | **Core** | **Bipolar** | **Axe Flake** | **Backed artefact** | **Yellow Ochre** | **Ground Ochre** | **Diversity** | |
| --- | --- | --- | --- | --- | --- | --- | --- | --- | --- | --- | --- | --- | --- | --- | --- | --- | --- |
| 1 | 5 | 48 | 43.63 | 0.91 | 13.19 | 48 |  |  |  |  |  |  |  |  | 2 | 1 |  |
| 2 | 10 | 162 | 176.4 | 1.09 | 27.39 | 157 | 1 |  | 1 |  | 2 | 1 |  |  | 1 | 5 |  |
| 3 | 15 | 234 | 391.25 | 1.67 | 83.76 | 227 | 2 |  | 1 |  | 1 | 3 |  |  | 5 | 5 |  |
| 4 | 20 | 111 | 364.42 | 3.28 | 64.56 | 109 |  |  | 1 |  | 1 |  |  |  |  | 2 |  |
| 5 | 25 | 202 | 839.53 | 4.16 | 219.34 | 192 | 3 | 1 | 3 | 2 |  |  | 1 |  | 1 | 6 |  |
| 6 | 30 | 233 | 764.53 | 3.28 | 280.47 | 227 | 1 | 4 |  | 1 |  | 0 |  |  |  | 4 |  |
| 7 | 35 | 145 | 1209.05 | 8.34 | 199.09 | 134 | 1 | 3 |  | 5 | 1 | 1 |  |  |  | 5 |  |
| 8 | 40 | 78 | 381.27 | 4.89 | 95.34 | 73 |  | 2 | 1 | 1 | 1 |  |  |  |  | 4 |  |
| 9 | 50 | 21 | 109.6 | 5.22 | 50.78 | 17 | 2 |  | 1 | 1 |  |  |  |  |  | 3 |  |
| 10 | 60 | 7 | 22.14 | 3.16 | 29.44 | 7 |  |  |  |  |  |  |  |  |  | 0 |  |
| 11 | 70 | 7 | 22.1 | 3.16 | 427.10 | 7 |  |  |  |  |  |  |  | 2 |  | 1 |  |
| 12 | 80 | 13 | 40.73 | 3.13 | 39.56 | 11 |  | 1 | 1 |  |  |  |  | 4 |  | 3 |  |
| 13 | 90 | 5 | 16.22 | 3.24 | 0.16 | 4 |  | 1 |  |  |  |  |  |  |  | 1 |  |
| 14 | 100 | 3 | 2.63 | 0.88 | 36.95 | 3 |  |  |  |  |  |  |  |  |  | 0 |  |
| 15 | 110 | 1 | 0.52 | 0.52 | 0.00 | 1 |  |  |  |  |  |  |  |  |  | 0 |  |
| 16 | 120 | 0 |  |  | 0.00 | 0 |  |  |  |  |  |  |  |  |  | 0 |  |
| 17 | 130 | 0 |  |  | 0.00 | 0 |  |  |  |  |  |  |  |  |  | 0 |  |
| 18 | 140 | 3 | 10.06 | 3.35 | 0.00 | 3 |  |  |  |  |  |  |  |  |  | 0 |  |
| 19 | 150 | 2 | 0.15 | 0.08 | 0.00 | 2 |  |  |  |  |  |  |  |  |  | 0 |  |
| 20 | 160 | 15 | 1.49 | 0.10 | 0.00 | 12 | 3 |  |  |  |  |  |  |  |  | 1 |  |
| 21 | 170 | 5 | 5.22 | 1.04 | 0.00 | 4 |  | 1 |  |  |  |  |  |  |  | 1 |  |
| 22 | 180 | 2 | 269.43 | 134.72 | 27.15 | 1 |  |  |  | 1 |  |  |  |  |  | 1 |  |
| 23 | 190 | 0 |  |  | 0.00 | 0 |  |  |  |  |  |  |  |  |  | 0 |  |
| 24 | 200 | 1 | 0.02 | 0.02 | 0.00 | 1 |  |  |  |  |  |  |  |  |  | 0 |  |
| 25 | 210 | 4 | 1.39 | 0.35 | 0.00 | 4 |  |  |  |  |  |  |  |  |  | 0 |  |
| 26 | 220 | 2 | 22.08 | 11.04 | 0.00 | 0 | 1 | 1 |  |  |  |  |  |  |  | 2 |  |
| Total |  | 1303 | 4693.86 | 3.60 | 1594 | 1244 | 14 | 14 | 9 | 11 | 6 | 5 | 1 | 6 | 9 |  |  |
| % |  |  |  |  |  | 95.5 | 1.1 | 1.1 | 0.7 | 0.8 | 0.5 | 0.4 | 0.1 | 0.5 | 0.7 |  |  |

|  |  |  |  |  |  |  |  |  |  |  |  |  |  |  |  |  |
| --- | --- | --- | --- | --- | --- | --- | --- | --- | --- | --- | --- | --- | --- | --- | --- | --- |


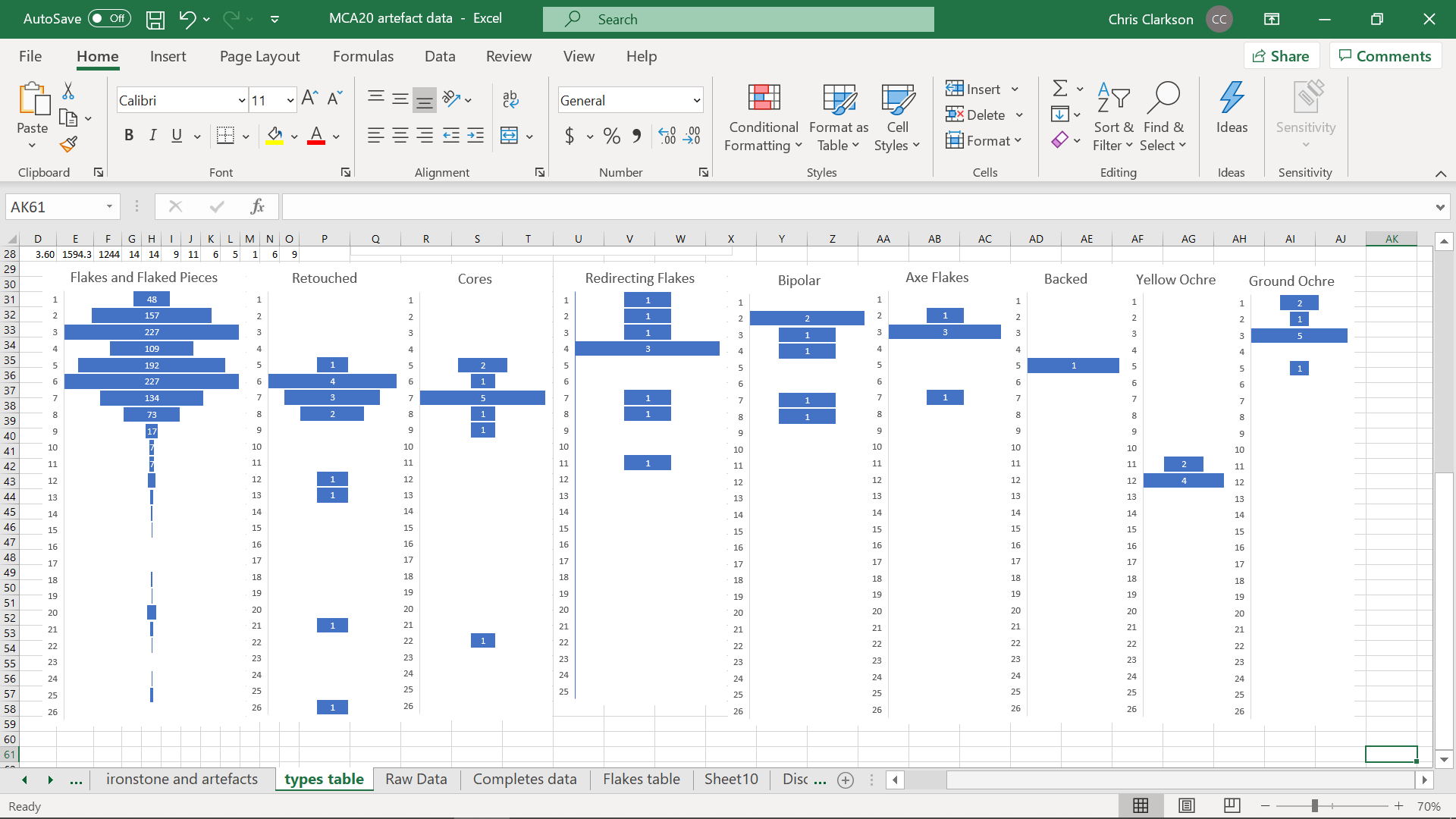


**Figure S12:** Changes in technological components at MCA20 through time.

Raw materials also change in frequency through time (Table S11, Figure S13), with quartz - the dominant raw material found down to Spit 22 (60%). Silcrete is the second most common raw material (14%) and occurs throughout the sequence but dominates the assemblage between Spits 14-19. Chert is another common raw material (12%) and appears in a variety of colours, textures and quality. Chert artefacts found in the lowest spits at MC20 are made of a pure white or white/pink stone also found in the lowest layers at Wallen Wallen Creek.

A number of other raw materials occur in the upper deposit in and above Spit 9. Rhyolite and greenstone occur on the island and appear only in the upper six spits. Quartzite is typically white or pale grey and of high quality and occurs in low numbers above Spit 9. Greater diversity in raw materials in the upper deposit likely reflects greater sample size (raw material diversity vs sample size: r^2^ = 0.821), but likely also reflects cultural preferences, and changing territories, access to stone, and trade with the mainland following sea level rise in the early to mid-Holocene.

**Table S11: Raw material changes at MCA20 by spit.**

| **Depth cm** | **White/Pink Chert** | **Chert** | **Silcrete** | **Rhyolite** | **Quartz** | **Crystal Quartz** | **Quartzite** | **Volcanic** | **Greenstone** |
| --- | --- | --- | --- | --- | --- | --- | --- | --- | --- |
| 5 |  | 6 | 4 |  | 28 |  | 1 |  |  |
| 10 |  | 27 | 21 |  | 84 |  | 5 | 2 |  |
| 15 |  | 29 | 25 |  | 122 |  | 11 | 2 |  |
| 20 |  | 10 | 7 |  | 86 |  | 4 |  | 2 |
| 25 |  | 9 | 35 | 3 | 150 |  | 1 | 1 | 1 |
| 30 |  | 40 | 38 | 4 | 148 | 1 | 1 |  |  |
| 35 |  | 33 | 24 | 2 | 79 |  | 5 | 1 |  |
| 40 |  | 10 | 19 |  | 48 |  | 1 |  |  |
| 50 |  | 3 | 4 | 1 | 18 |  |  | 1 |  |
| 60 |  | 1 | 3 |  | 3 |  |  |  |  |
| 70 |  |  |  |  | 5 |  |  |  |  |
| 80 |  |  | 1 |  | 8 |  |  |  |  |
| 90 |  |  |  |  | 5 |  |  |  |  |
| 100 |  |  | 2 |  | 1 |  |  |  |  |
| 110 |  |  | 1 |  |  |  |  |  |  |
| 120 |  |  |  |  |  |  |  |  |  |
| 130 |  |  |  |  |  |  |  |  |  |
| 140 |  |  | 3 |  |  |  |  |  |  |
| 150 |  |  | 2 |  |  |  |  |  |  |
| 160 | 9 | 4 | 2 |  |  |  |  |  |  |
| 170 | 2 | 1 | 2 |  |  |  |  |  |  |
| 180 |  |  |  |  | 1 |  |  |  |  |
| 190 |  |  |  |  |  |  |  |  |  |
| 200 | 1 |  |  |  |  |  |  |  |  |
| 210 | 2 |  | 2 |  |  |  |  |  |  |
| 220 |  | 1 |  |  |  |  |  |  |  |
| Total | 16 | 158 | 195 | 10 | 786 | 1 | 29 | 3 | 3 |
| % | 1.23 | 12.13 | 14.97 | 0.77 | 60.32 | 0.08 | 2.23 | 0.23 | 0.23 |

**Figure S13:** Stacked bar graph showing changes in the proportion of raw materials at MCA20 by spit.

**Post-Depositional Disturbance**

There is always the possibility that the layering of artefacts observed in a soft sand dune may be the result of downward movement or displacement through water, bioturbation or other site formation mechanisms such as clearing or mining activities. In the case of MCA20, there is good reason to believe the lower assemblage has not derived from the upper assemblage due to changes in raw materials and weathering. The chert found in the lower assemblage is a highly weathered white chert that rarely occurs in the upper assemblage above Spit 10 and never in a weathered state. Types believed to by mid-to-late Holocene in age also occur only in the upper assemblage (axe flakes and a backed artefact).

One further way to examine whether there has been disturbance in the form of downward displacement of artefacts through time is to examine whether there is size sorting of artefacts with depth, potentially reflecting differential movement of large or small artefacts as observed in at least some cases where bioturbation, site disturbance of water action in known to have moved artefacts in a site over time. At MCA20, there is no evidence of size sorting with depth (Figure S14), with artefact mass peaking in tandem with artefact abundance. Artefact size ranges increase with each artefact pulse, as is typically found in archaeological sites where larger artefacts are rare and typically occur only in larger assemblages.


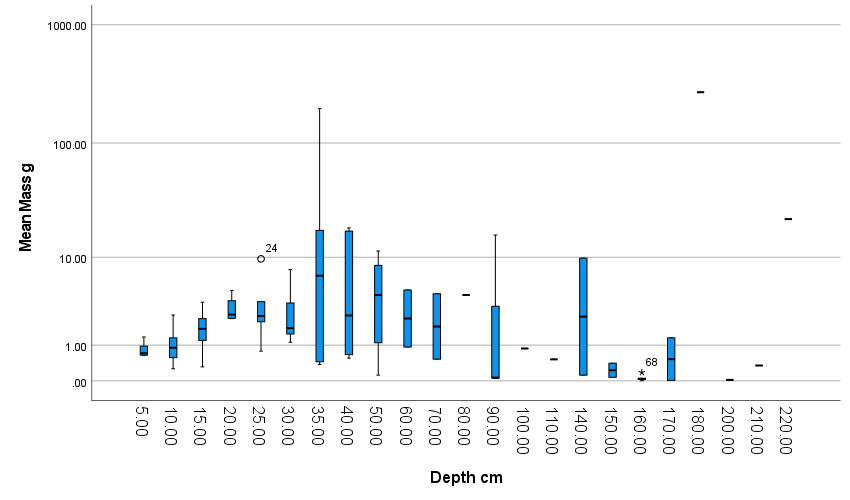


**Figure S14:** Box plot of distribution of mean artefact mass with depth.

**Technological Characteristics of the Flaked Stone Assemblage**

Notable differences in size and technology occur at MCA20 according to raw material type. This no doubt reflects the size of available stone clasts, the quality of the stone for flaking and its availability (distance to source, ease of access, etc). Differences in the size of stone artefacts of different raw materials are evident when the mass of complete artefacts made from different raw materials is examined. In Figure S15, we see that quartz artefacts are the largest, followed by rhyolite, chert and silcrete. Quartzite and volcanic flakes are the smallest artefacts in the sample. Quartz likely has a local or nearby origin given high levels of cortex. Ironstone is sometimes found concreted to quartz pebbles at the site, suggesting they may have a similar source nearby, such as a beach platform or eroding gravel bed.


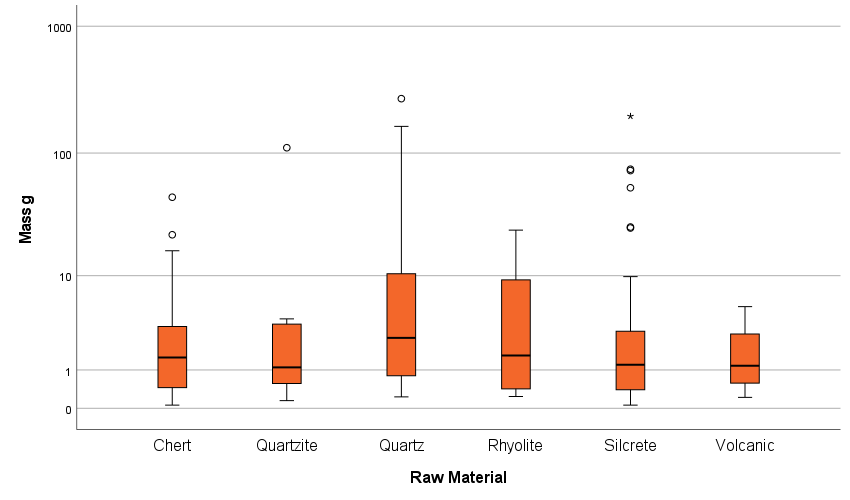


**Figure S15:** Box plot of artefact mass for complete artefacts >1cm in maximum dimension.


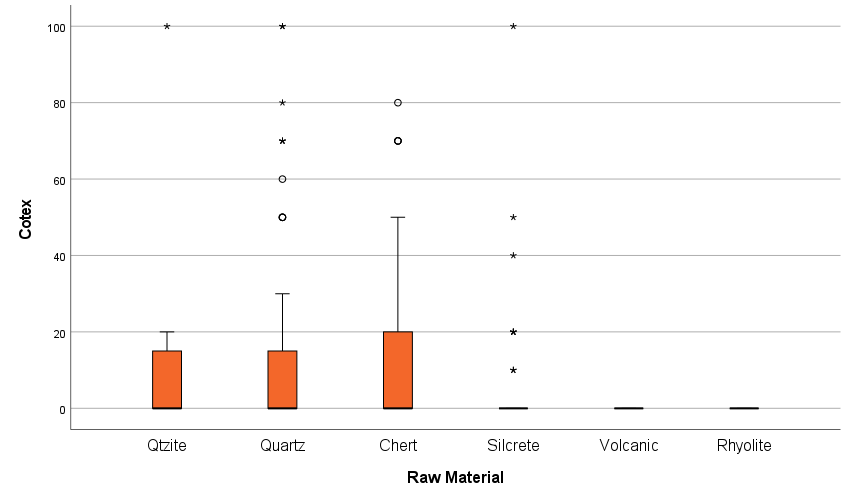


**Figure S16:** Proportions of cortex found on complete artefacts >1cm at MCA20.

Cortex appears on 25% of artefacts in the assemblage but is unevenly distributed on different raw material types (Figure S16, S17). Cortex is most abundant on quartzite, quartz and chert artefacts, but almost entirely absent on silcrete and entirely absent on volcanic and rhyolite artefacts. Cortex is mostly angular in type on chert artefacts suggesting a rock outcrop or near-rock outcrop origin, but is predominantly rounded for quartzite, quartz and silcrete artefacts, suggesting a water-rolled procurement context.


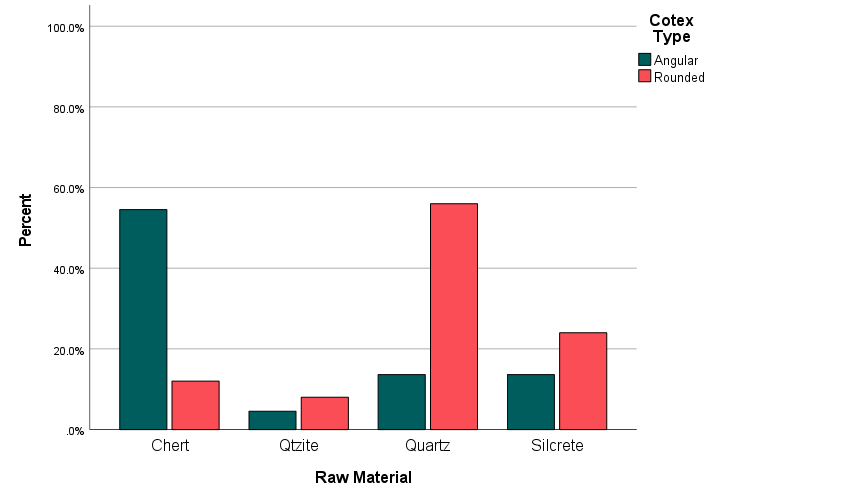


**Figure S17:** Types of cortex found on complete artefacts >1cm at MCA20.

**Cores**

There are 11 cores in the assemblage, eight of which are complete with summary measurements provided below (Table S12). The majority are made from quartz (N = 9), with one quartzite and one silcrete core (Figure S18). Cores are typically small at under 110g and under 50mm in maximum dimension. There is very little cortex on cores (mean = 20%) suggesting they were heavily reduced. All but one are multiplatform cores with on average 3.7 ± 1.8 rotations and 11 flake scars. In quartz cores at least, the more platforms they have the lighter they are (r^2^ = 0.571). Final platform angles are on average 90 degrees and around a third of scars are step terminated, which together indicates cores were close to exhaustion. The silcrete core is nearly double the size of the quartz and quartzite cores and has a large heat break on one side. The flakes struck from these cores prior to discard are quite small at around 27 ± 10 mm.

**Table S12:** Summary statistics for cores by raw material types at MCA20.

| **Raw Material** | **Quartzite N =1** | **Quartz N=8** | | **Silcrete N=1** | **Total N =10** | |
| --- | --- | --- | --- | --- | --- | --- |
|  | Mean | Mean | SD | Mean | Mean | SD |
| **Weight** | 110.34 | 79.04 | 91.97 | 195.80 | 93.85 | 89.21 |
| **Maximum Dimension** | 67.00 | 44.27 | 20.83 | 71.00 | 49.21 | 21.14 |
| **% Cortex** | 20.00 | 22.50 | 33.27 | 0.00 | 20.00 | 30.18 |
| **Length** | 65.00 | 43.45 | 19.78 | 69.00 | 48.16 | 20.09 |
| **Width** | 53.00 | 37.40 | 15.99 | 58.00 | 41.02 | 16.08 |
| **Thickness** | 40.00 | 28.63 | 10.52 | 44.00 | 31.30 | 10.90 |
| **Platform Width** | 66.00 | 35.64 | 24.58 | 62.00 | 43.74 | 24.40 |
| **Platform Thickness** | 53.00 | 26.31 | 18.73 | 65.00 | 35.65 | 22.37 |
| **Number of Scars** | 23.00 | 8.63 | 5.18 | 22.00 | 11.40 | 7.43 |
| **# Step Terminations** | 6.00 | 3.25 | 2.38 | 7.00 | 3.90 | 2.51 |
| **Longest Scar** | 39.00 | 24.30 | 7.90 | 43.00 | 27.64 | 9.95 |
| **Number of Platforms** | 2.00 | 3.75 | 1.91 | 5.00 | 3.70 | 1.83 |
| **Final Platform Angle** | 67.00 | 92.13 | 9.17 | 100.00 | 90.40 | 11.80 |

**
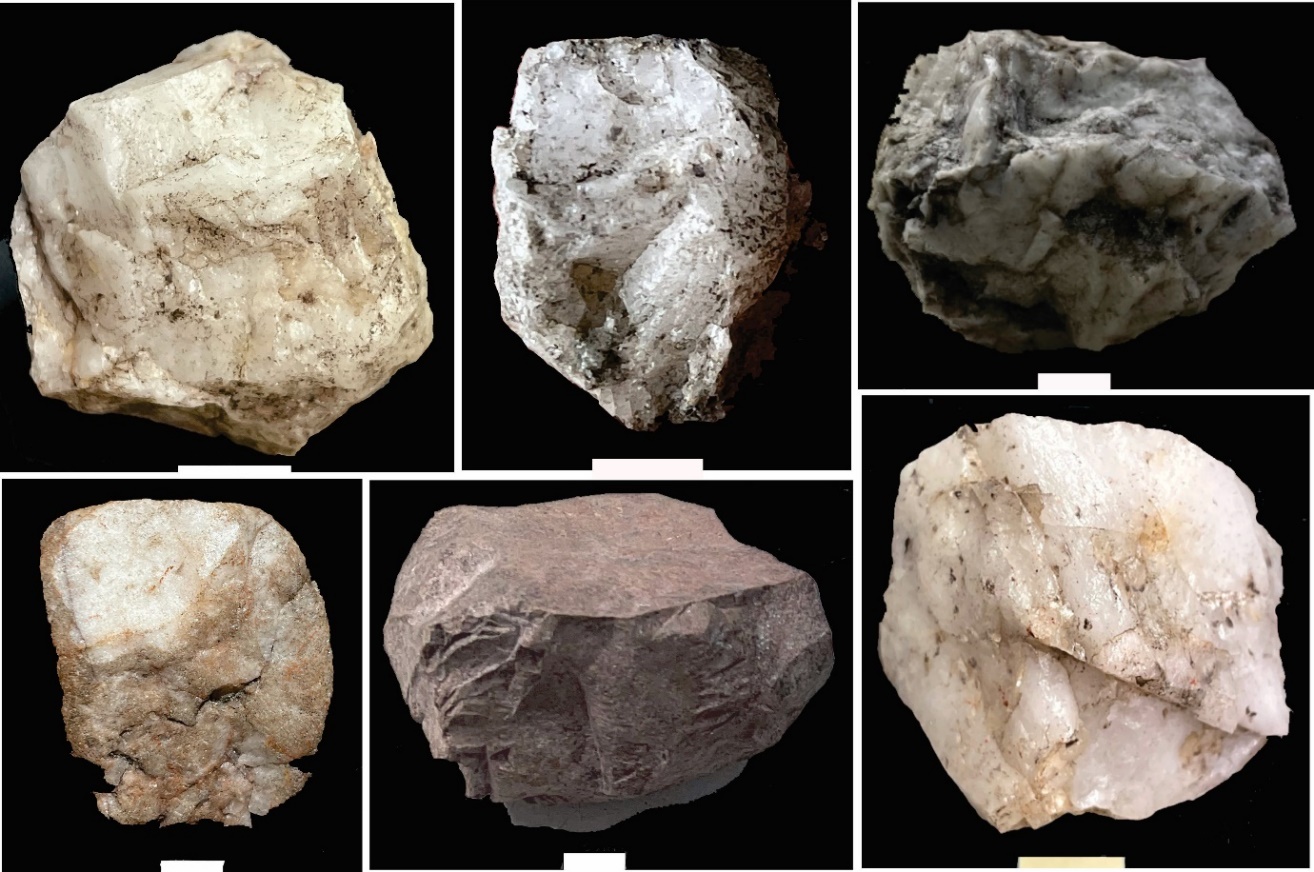
**

**Figure S18:** Examples of quartz and silcrete multiplatform cores from MCA20. All from XU7 except bottom left which is from XU5, top right is from XU22, and bottom right which is from XU4.

**Flakes**

Flakes are the most numerous stone artefact type in the assemblage. They are mostly of small size, averaging 3 ± 6 g and 22 ± 10 mm in maximum dimension (Table S13). Flakes typically are free from cortex, but those that do have cortex (25.7%) have only 9 ± 20 % cortex. The combination of small size and low proportions of cortex is consistent with rationing of raw material and limited access to replacement stone. Flakes are as wide as they are long (16 x 16mm) and around a third as thick as they are wide (5mm). Platforms are typically broad and thick given the average size of flakes, with platform angles of 70 ± 12 degrees. Dorsal surfaces typically show 3.7 ± 1.7 dorsal scars. Most flakes have feather terminations (84%) (Table S14). Platforms are typically single conchoidal (47%) or multiple conchoidal/dihedral (28%) scars, consistent with being struck from multiplatform cores, although crushed, cortical and focalised platforms occur in under 10% of cases each (Table S15). Platform preparation is common and typically overhang removal (46%) with some faceting (8%) (Table S16). Dorsal scars derive from the proximal end in 91% of cases (Table S17).

**Table S13:** Summary statistics for complete flakes > 1cm by raw material type**.**

| **Raw Material** | | **N** | **Weight** | **Max Dim (mm)** | **% Cortex** | **Length (mm)** | **Width (mm)** | **Thickness (mm)** | **Platform Width** | **Platform Thickness** | **Platform Angle** | **Dorsal Scars** |
| --- | --- | --- | --- | --- | --- | --- | --- | --- | --- | --- | --- | --- |
| Chert | Mean | 47 | 4.08 | 24.03 | 11.96 | 18.42 | 17.53 | 5.78 | 11.28 | 4.41 | 69.27 | 3.77 |
|  | SD |  | 7.70 | 11.31 | 22.17 | 9.79 | 9.60 | 4.16 | 6.90 | 2.81 | 15.72 | 1.55 |
| Quartzite | Mean | 6 | 1.61 | 21.02 | 18.33 | 20.22 | 14.81 | 3.15 | 10.11 | 2.43 | 73.67 | 4.00 |
|  | SD |  | 1.61 | 7.28 | 40.21 | 6.80 | 5.95 | 1.78 | 9.16 | 1.65 | 13.02 | 2.28 |
| Quartz | Mean | 35 | 3.17 | 21.96 | 12.29 | 16.55 | 16.81 | 5.52 | 9.91 | 4.25 | 75.39 | 3.83 |
|  | SD |  | 4.99 | 11.04 | 25.22 | 5.89 | 9.77 | 3.53 | 5.46 | 2.50 | 13.15 | 1.64 |
| Silcrete | Mean | 60 | 1.48 | 19.84 | 4.50 | 14.95 | 15.30 | 3.89 | 10.07 | 3.26 | 69.65 | 3.85 |
|  | SD |  | 2.03 | 7.36 | 15.56 | 6.16 | 6.12 | 2.06 | 5.02 | 1.53 | 9.27 | 1.76 |
| Total | Mean | 166 | 3.09 | 22.14 | 8.85 | 16.94 | 16.84 | 5.05 | 10.93 | 4.00 | 70.52 | 3.77 |
|  | SD |  | 6.02 | 10.31 | 20.96 | 7.88 | 9.07 | 3.51 | 6.83 | 2.51 | 12.83 | 1.66 |

**Table S14:** Frequency of termination types for complete flakes >1cm maximum dimension.

| **Termination Type** | **Frequency** | **Percent** |
| --- | --- | --- |
| Feather | 139 | 83.7 |
| Hinge | 7 | 4.2 |
| Step | 19 | 11.4 |
| Total | 166 | 100.0 |

**Table S15:** Frequency of platform types for complete flakes >1cm maximum dimension.

| **Platform Type** | **Frequency** | **Percent** |
| --- | --- | --- |
| Cortical | 14 | 8.4 |
| Crushed | 13 | 7.8 |
| Dihedral | 6 | 3.6 |
| Focalised | 9 | 5.4 |
| Multiple Conchoidal | 43 | 25.9 |
| Single & Cortical | 2 | .12 |
| Single Conchoidal | 78 | 47.0 |
| Total | 166 | 100.0 |

**Table S16:** Frequency of platform preparation for complete flakes >1cm maximum dimension.

| **Platform Preparation** | **Frequency** | **Percent** |
| --- | --- | --- |
| Faceting & Overhang Removal | 13 | 7.8 |
| Faceting | 5 | 3.0 |
| Overhang Removal | 93 | 46 |
| Total | 166 | 100.0 |

**Table S17:** Frequency of dorsal scar patterning for complete flakes >1cm maximum dimension.

| **Dorsal Scar Pattern** | **Frequency** | **Percent** |
| --- | --- | --- |
| Bidirectional | 2 | 1.2 |
| Cortical | 1 | .6 |
| Distal | 1 | .6 |
| Non-Proximal | 5 | 3.5 |
| Proximal | 151 | 91.0 |
| Proximal & Ridge | 1 | .6 |
| Total | 166 | 100.0 |

**Redirecting Flakes**

Redirecting flakes are formed when cores are rotated to create a new platform and remove part of an old platform edge in the process, preserved on the dorsal surface. These flakes are quite common at the site (N = 9), consistent with a high degree of core rotation (Table S18). In the sample of complete artefacts, redirecting flakes are most commonly identified on silcrete and quartz, although the actual number of quartz redirecting flakes is higher but the flakes were broken due to the high rate of shatter on the generally low quality quartz used at the site. Redirecting are typically thicker than regular flakes, show no cortex, have higher numbers of dorsal scars and preserve steep old platform angles on the dorsal surface consistent with core rotation occurring when platforms were becoming unworkably steep. As redirecting flakes are often among the larger flakes in the assemblage, they are often retouched as larger flakes are almost always selected for retouch. Three of the nine redirecting flakes show retouch.

**Table S18. Summary statistics for complete redirecting flakes >1cm at MCA20.**

| **Raw Material** | **Quartz** |  | **Silcrete** |  | **Total** |  |
| --- | --- | --- | --- | --- | --- | --- |
|  | **N = 2** |  | **N = 3** |  | **N = 5** |  |
|  | Mean | SD | Mean | SD | Mean | SD |
| Weight | 3.79 | 1.02 | 4.89 | 0.85 | 4.45 | 0.99 |
| Max Dim (mm) | 26.26 | 1.12 | 30.49 | 3.57 | 28.79 | 3.47 |
| % Cortex | 0.00 | 0.00 | 0.00 | 0.00 | 0.00 | 0.00 |
| Length | 20.99 | 7.67 | 22.86 | 4.83 | 22.11 | 5.23 |
| Width | 16.39 | 2.45 | 22.43 | 8.53 | 20.01 | 6.99 |
| Thickness | 9.35 | 1.92 | 8.44 | 2.98 | 8.81 | 2.37 |
| Platform Width | 12.25 | 10.67 | 9.15 | 8.15 | 10.70 | 7.95 |
| Platform Thickness | 5.65 | 3.78 | 4.60 | 4.14 | 5.12 | 3.30 |
| Platform Angle | 71.50 | 9.19 | 53.50 | 4.95 | 62.50 | 12.01 |
| # Dorsal Scars | 5.00 | 1.41 | 8.33 | 6.11 | 7.00 | 4.74 |
| Old Dorsal Platform Angle | 99.00 | 9.89 | 92.00 | 1.00 | 94.80 | 6.30 |
| % Platform Preparation |  |  |  |  | 20 |  |
| % Single Conchoidal Scar |  |  |  |  | 40 |  |
| % Feather Termination |  |  |  |  | 100 |  |

**
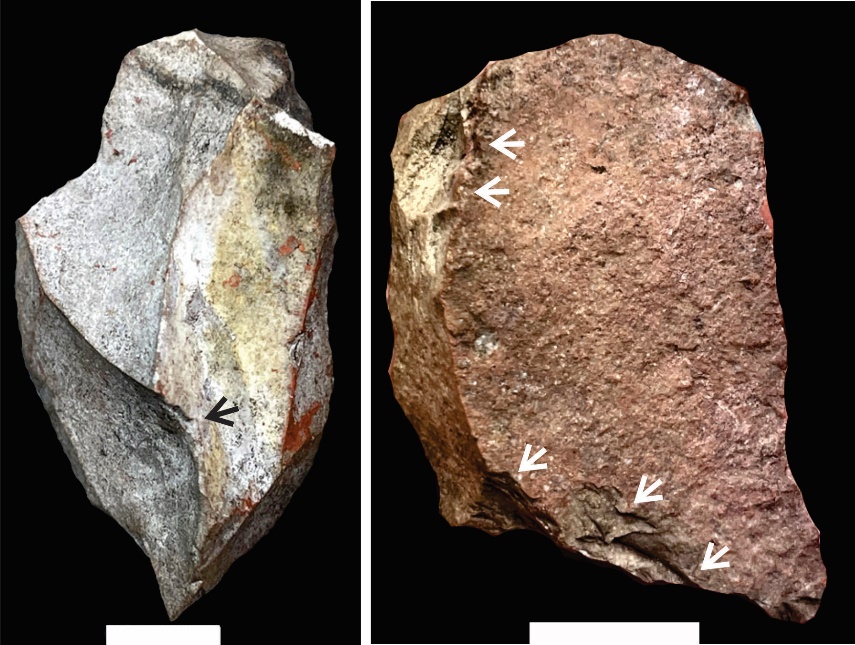
**

**Figure S19:** Silcrete redirecting flake from XU12. Flake on the right is also retouched and shown in Figure S18. Arrows mark initiations of old platforms on the dorsal surface.

**Retouched Flakes**

There are 14 retouched flakes in the assemblage (Table S19, Figure S20) and are found from Spits 5 through 26. Summary statistics are provided for the 11 complete specimens in Table 11. Nine of the 11 complete retouched flakes are made from silcrete, with only two made from quartz. These tend to be much larger in mass and linear dimensions than average flakes and were likely selected for retouch for their greater size. Retouched flakes have no cortex, are 40 ± 18 mm in length, 24 ± 31 g in weight and are slightly more elongate than ordinary flakes. Retouch characteristics indicate steep edge angles (87 ± 8 degrees), with around 50% of the perimeter retouched. Kuhn’s GIUR shows extensive retouch on the edges with most flakes possessing at least one notch (1.5 ± 1). Both ventral and bifacial retouch is common and extensive in some cases (Figure S20, left middle, top right).

**Table S19:** Summary statistics for complete retouched flakes >1cm from the MCA20 assemblage.

| **Attribute** | **Mean** | **SD** |
| --- | --- | --- |
| Weight | 24.31 | 31.30 |
| Max Dim (mm) | 40.32 | 18.35 |
| % Cortex | 0.00 | 0.00 |
| Length | 35.86 | 16.05 |
| Width | 26.72 | 14.92 |
| Thickness | 12.01 | 7.29 |
| Platform Width | 23.76 | 15.10 |
| Platform Thickness | 6.51 | 3.99 |
| Platform Angle | 74.25 | 21.47 |
| # Dorsal Scars | 4.17 | 2.64 |
| Retouched Edge Angle | 87.13 | 8.11 |
| # Notches | 1.50 | 1.05 |
| GIUR | 0.77 | 0.18 |
| % Retouch Perimeter | 0.54 | 0.34 |
| % Feather Termination | 100.00 |  |
| % Platform Preparation | 37.00 |  |
| % Single Conchoidal Platform | 25.00 |  |
| % Proximal Scar Orientation | 100.00 |  |


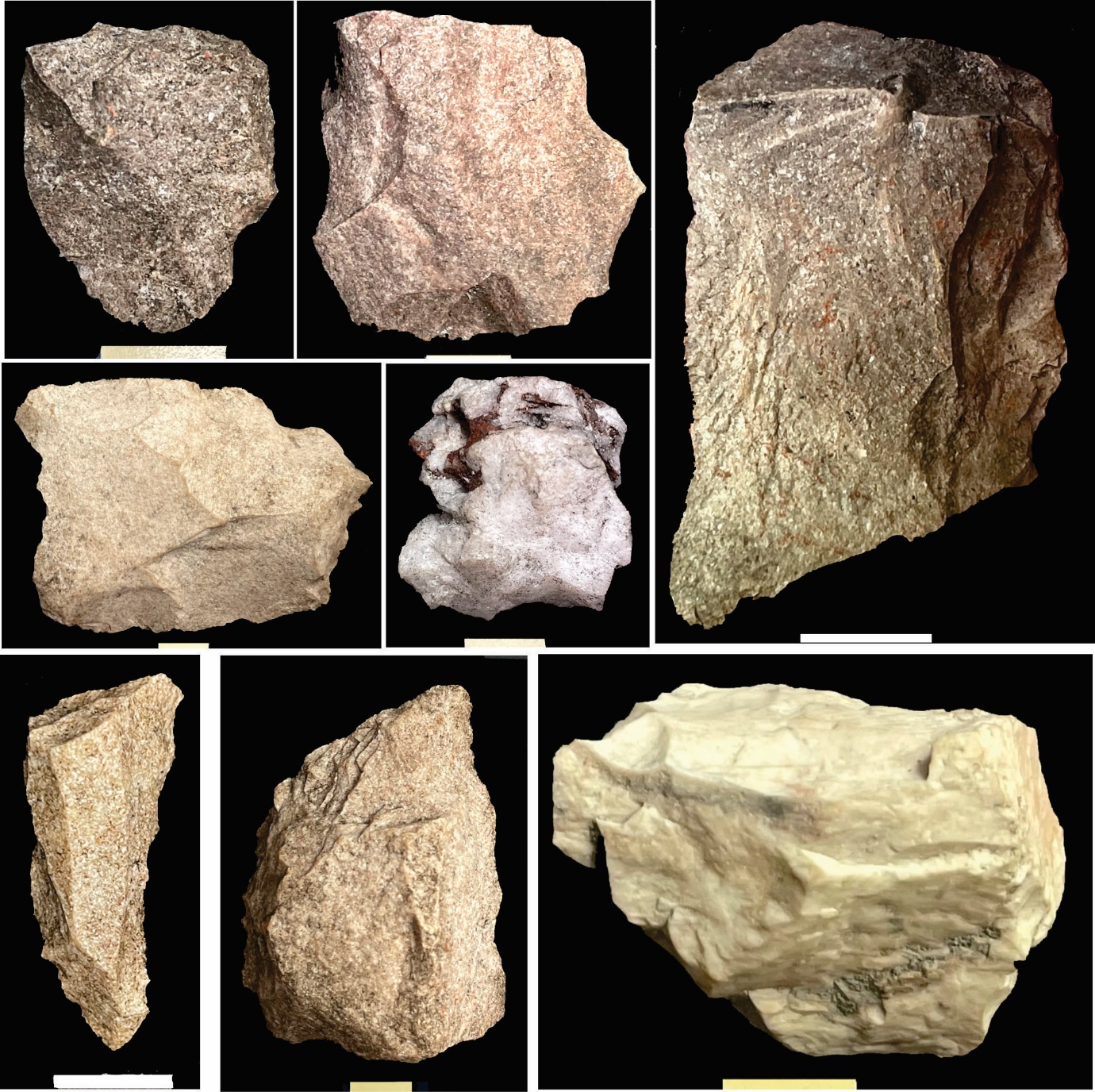


**Figure S20:** Retouched flakes from MCA20. All silcrete unless indicated. Top left: Notch, XU6; top middle: bifacial notched scraper, XU7; top right: ventral notched scraper, XU5; second row left: bifacial notched scraper; second row middle: quartz notch, XU13; bottom left: double sided steep edged scraper, XU6; bottom middle: double side and double end steep edged scraper, XU6; bottom right: chert steep end scraper, XU26.

**Backed Artefact**

A single backed artefact was found in the assemblage in Spit 5 (Figure S21). These are artefacts made in the mid to late Holocene in eastern Australia, indicating the upper 5 sits are likely less than 5000 years old. The backed artefact is made from silcrete and only minimally bidirectionally backed around the proximal end of one margin.

**
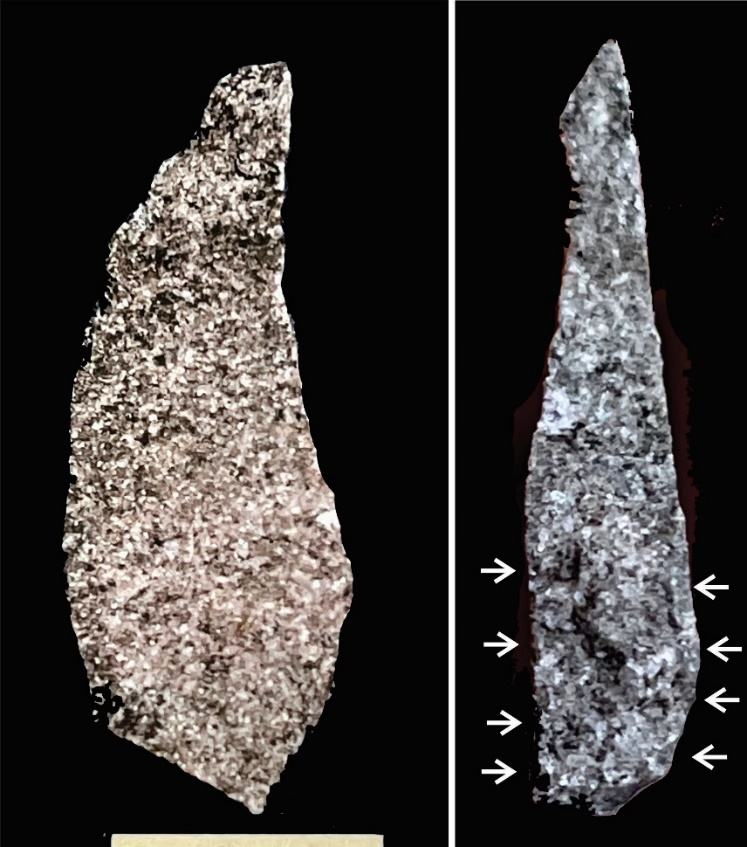
**

**Figure S21:** Backed artefact from XU5.

**Axe Flakes**

Four axe flakes were recovered in the MCA20 assemblage. One is of greenstone and the rest are of volcanic rock of unknown type (Figure S22). Axe flakes occur only in the upper 7 spits. One axe flake shows grinding on the platform but not the dorsal surface, whereas others are ground only on the dorsal surface. This indicates flakes were likely struck the edge and sides of axes that were undergoing rejuvenation.


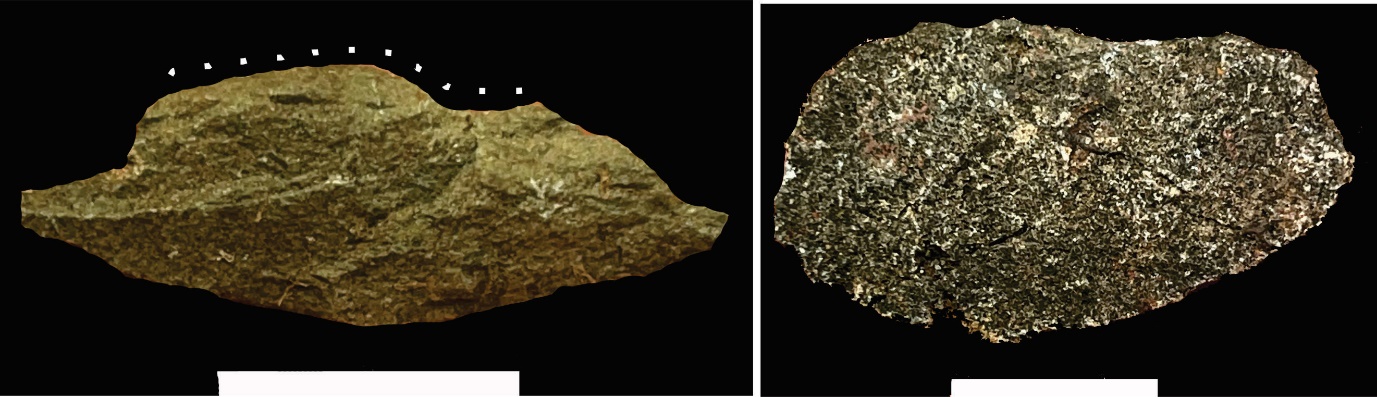


**Figure S22: Left:** Flake with grinding on the platform, indicated with dotted line. Right: flake with a ground dorsal surface.

**Heat Treatment Identification**

Silcrete artefacts excavated from both WWC and MCA20 were analysed for evidence of intentional heat treatment. The method employed for this analysis was based of the visual identification and replica tape 3D scanning microscopy procedure developed by Schmidt^[49]^. This method has been successfully used to identify intentional silcrete heat treatment at multiple sites around Australia and South Africa^[49,50,51,52]^. The method involved two steps: a visual examination and identification of silcrete artefacts from each site, followed by 3D scanning microscopy of surface imprints taken from negative flake scars using foam replica tape.

The visual analysis relied on identification of ‘diagnostic pieces’ found among the assemblages. Thermal alteration typically reduces fracture surface roughness, so flakes removed prior to heating produce visibly rougher negative scars than those removed after heating^[53]^. Diagnostic pieces therefore exhibit a smooth scar superimposed over an older rough scar, which can only result from deliberate heat treatment occurring between knapping phases^[49]^. Such diagnostic pieces have long been used as evidence of intentional heat treatment^[54]^. Each silcrete artefact with a maximum length >10 mm was visually examined for evidence of heat treatment based on comparisons with the known surfaces of the diagnostic pieces. The primary proxy for heat treatment was surface roughness, though colour, lustre, and heat-induced non-conchoidal fractures were also considered in the identification of each artefact.

The results of the visual identification were confirmed by 3D scanning microscopy of surface imprints taken on samples of artefacts from both sites. These samples included all artefacts with an even surface that was large enough for the 1 x 1 cm measurement area of the replica tape. *Testex Press-O-Film* replica tape was employed for this task as it is an inexpensive and efficient technique that eliminates the need to scan artefacts directly. Scanning of the imprints was undertaken using a *Solutionix C500* structured light 3D scanner with a 90mm FOV lens. The scans were processed in *Gwyddion*, an open-source scanning probe microscopy software. A total of 54 silcrete artefacts were analysed, with 42 measurements taken on 35 artefacts from WWC and 30 measurements taken on 19 artefacts from MCA20. For diagnostic pieces, imprints were taken of the ventral surface and at least one pre-HT dorsal scar. For non-diagnostic pieces, one imprint was taken of the ventral surface.

Two parameters were calculated from each scan: mean areal roughness (*S*_a_) and dimensionless differential entropy (*H*). These data were analysed with a variety of statistical methods in the freely available software package PAST (PAleontological STatistics) version 4. The natural logarithms of the *S*_a_ values [LN(*S*_a_)] were used since these are normally distributed for both sites, while the raw data are not. LN(*S*_a_) was plotted against *H* to produce a linear function. Cut-off values were determined along this function based on the maximum heated roughness and minimum unheated roughness values of known surfaces. Non-diagnostic artefacts falling above or below these cut-offs were assigned as unheated or heated, respectively. Non-parametric Mann-Whitney tests for equal medians were used to determine the significance of roughness differences between heated and unheated pieces from both sites. The influence of inter-site silcrete variation and of heat treatment on surface roughness were explored with a two-way fixed effects ANOVA. A logistic regression was employed to estimate the prediction power of roughness measurements and calculate probabilities of DHT for unknown artefacts.

**8. WWC Fauna and Shell**

Vertebrate fauna remains were identified using comparative skeletal reference materials held at the Queensland Museum and the University of Queensland Archaeology Zooarchaeology Laboratory. Any identifiable teeth, cranial and post-cranial specimens were assigned to either their highest taxonomic level or body-size. Specimens were examined for percussion and cutmarks, along with other predator damage such as gastric etching or carnivore marks, using an Olympus LG-PS2 low-powered microscope.

The total weight of the assemblage was 460.24 g, which was mostly recovered from the upper 23 excavation units (XUs). Of the 3133 specimens recovered, 851 (27%) were identifiable to some degree, with the majority of these assigned as bony fish (n=702). The composition of the identified taxa is similar to what is found on Minjerribah today^[55]^. Identified terrestrial fauna included long-nosed bandicoot (*Perameles*), brushtail possum (*Trichosurus vulpecula*), dingo (*Canis familiaris*) and unidentified small macropods, while marine fauna comprise dugong (*Dugon dugon*), sea turtle (Cheloniidae), tarwhine (*Rhabdosargus sarba)*, whiting (c.f*. Sillago ciliate*), catfish (Siluriformes) and bream (Sparidae) (Table S20). Most specimens were below 1cm in length, indicating the highly fragmentary nature of the assemblage. No specimens were identified with percussion, tool, carnivore or rodent marks. Approximately 25% of the bone was burned, with 15% of specimens being carbonised (n=455) and 10% calcined (n=301).

The molluscan assemblage available for analysis comprised 42.95 g of shell from a total of 112 XUs across 22 excavation squares. Ten marine taxa were identified to genus/species, and a further six to family (Table S21). Three species of land snail were observed but could only tentatively be ascribed to the family Camaenidae for the larger of the species, and to cf. Charopidae for the two smaller species. For the most part the shell assemblage is highly fragmented and may represent the fine sieve fraction.

**Table S20:** Wallen Wallen vertebrate taxa

| **Taxon** | **Common Name** | **NISP** |
| --- | --- | --- |
| *Perameles* | long-nosed bandicoot | 1 |
| *Trichosurus vulpecula* | brushtail possum | 1 |
| *Canis lupus c.f. dingo* | dingo | 1 |
| *Dugong dugon* | dugong | 5 |
| Small macropodidae | small wallaby | 2 |
| Small-medium mammal |  | 18 |
| Micromammal |  | 6 |
|  |  |  |
| Aves | bird | 1 |
| Chelonioidea | sea turtle | 35 |
| Testudines | freshwater turtle | 2 |
| Varanidae | goanna | 2 |
| Pythonidae | python | 6 |
| Serpentes | snake | 37 |
| Reptilia |  | 32 |
|  |  |  |
| *Rhabdosargus sarba* | Tarwhine | 81 |
| c.f. *Sillago ciliata* | Whiting | 1 |
| Sparidae | Sparid | 14 |
| Siluriformes | Catfish | 2 |
| Osteichthyes | Bony fish | 604 |
| Total |  | 851 |

**Table S21:** Wallen Wallen molluscan taxa

| **Class** | **Family** | **Taxon** |
| --- | --- | --- |
| Bivalvia (M) | Arcidae | Arcidae |
|  | Mytilidae | *Trichomya hirsuta* |
|  | Ostreidae | *Saccostrea glomerata* |
|  | Psammobiidae | *Plebidonax deltoides* |
|  | Pteriidae | *Isognomon* spp. |
|  |  | *Pinctada* spp. |
| Gastropoda (M) | Batillariidae | Batillariidae |
|  |  | *Batillaria australis* |
|  |  | *Pyrazus ebeninus* |
|  | Calliostomatidae | *Astele* spp. |
|  | Littorinidae | *Bembicium* spp. |
|  | Patellidae | Patellidae |
|  | Ranellidae | Ranellidae |
|  | Trochidae | Trochidae |
|  |  | *Austrocochlea* spp. |
|  | Vermetidae | Vermetidae |
| Gastropoda (T) | Camaenidae/  Charopidae | Camaenidae/  Charopidae |

**9. MCA20 Fauna and Shell**

MCA20 is only the second midden deposit from the west coast of Minjerribah to be analysed and reported. In total 450.79 g of bone and 17804.21 g of shell was recovered from constrained strata throughout the MCA20 profile. Vertebrate fauna remains were identified using comparative skeletal reference materials held at the University of Queensland Archaeology Zooarchaeology Laboratory. Any identifiable teeth, cranial and post-cranial specimens were assigned to their highest taxonomic level. Specimens were weighed individually with scientific scales accurate to 0.01 g. Specimens were examined for percussion and cutmarks, along with other predator damage such as gastric etching or carnivore marks, using an Olympus LG-PS2 low-powered microscope.

The total weight of the assemblage was 450.79 g, representing 267 specimens and primarily distributed in the NW and ENE squares between XU4 and XU7. The faunal remains were fragmented and weathered with only 21 specimens being identified with certainty. The vertebrate fauna comprises dugong (*Dugong dugon,* n=5*)*, bird (Aves, n=1), sea turtle (Chelonioidea, n=4), python (Pythonidae, n=3), skates and rays (Rajiformes, n=4), and non-diagnostic bony fish (Osteichthyes, n=4). Approximately 31% (n = 82) of all bone was burned, with 25% of specimens (n = 68) calcined and the rest (n = 14) carbonised. This higher proportion of calcined remains suggests degradation and loss of non-burned bone^[56]^.

A conservative approach to taxonomic identification of the molluscan elements was employed, with care taken to avoid the over-identification of specimens. Except for the land snails, most of the molluscs were identified to species; however, shell fragments whose taxa were indeterminate due to a lack of preserved diagnostic features were bagged together and weighed. Specimens for which the taxon was determined were weighed and the numbers of identifiable specimens (NISP) and minimum number of individuals (MNI) counted. The method of calculating MNI followed the protocols formalised by Harris^[57]^. Additionally, specimens that were taxonomically identifiable but did not retain the necessary non-repeating element for MNI calculation were noted as being present, and an MNI of 1 assigned per taxon for each individual excavation unit.

Molluscan material was concentrated within the upper ten XUs (0-60 cm) and exhibited ten identifiable taxa dominated by Hercules Club mud whelk (*Pyrazus ebeninus*) (Table S22). The species accounted for 16722.13 g of the total shell weight (93.92%), 84.64% of total NISP, and 92.5% of the total MNI. There were no juvenile molluscan specimens recovered from the assemblage, suggesting targeted hunting strategies and potential long-term management strategies. Targeted harvesting ensures that the next generation of molluscs is not depleted or wiped out and is therefore a form of resource management. The midden deposit commences 7 cm above the OSL date of 5830 ± 650 years, with a peak in deposition at the OSL date of 2130±290 years.

**Table S22:** MCA20 molluscan taxa

| **Class** | **Family** | **Taxon** |
| --- | --- | --- |
| Bivalvia (M) | Arcidae | *Anadara trapezia* |
|  | Chamidae | *Chama* sp. |
|  | Mytilidae | *Trichomya hirsuta* |
|  | Ostreidae | *Saccostrea glomerata* |
|  | Psammobiidae | *Plebidonax deltoides* |
| Gastropoda (M) | Batillariidae | *Pyrazus ebeninus* |
|  | Cerithoidea | Cerithoidea |
|  | Naticidae | Naticidae |
|  | Vermetidae | Vermetidae |
| Gastropoda (T) | cf Charopidae | cf Charopidae |

**10. Minjerribah Palaeoecology**

Minjerribah has an extensive number of palaeoecological sites, with 16 wetland sites cored and dated^[59]^. Seven of these sites have continuous deposition that incorporates the LGM, suggesting a positive moisture balance for the island during this arid phase^[58,59]^. One of these sites, Native Companion Lagoon, is situated on the southeast coast of the island, ~7 km south of the MCA20 site and ~14 km southwest of Wallen Wallen. It exhibits a 45,000-year vegetation and fire history providing landscape context for both the MCA20 and Wallen Wallen archaeological data.

The revised age model is presented in Fig. S23 with Marine Isotope Stages 3 to 1. For the duration of this record Casuarinaceae and eucalypt pollen (both major components of the sclerophyll arboreal taxa group) are the dominant taxa, reflecting open sclerophyll forest and woodland. Rainforest, primarily *Araucaria*, maintain relatively high values from until 20 ka, suggesting the nearby presence of a rainforest community (potentially on the dry Moreton Plain). There is a sharp decline in rainforest after 20 ka, which may reflect the influence of arid LGM climates and rising sea level that occurred during the Last Glacial Interglacial Transition. Mangroves are represented in the record from around 10 ka and reflect the development of Moreton Bay in response to rising sea levels. Drier climates are seen between 35 to 21 ka in the Native Companion Lagoon record, with the decline in rainforest taxa, although rainforest does recover at the Native Companion Lagoon site between 21 to 20 ka, reflecting variable LGM climate/vegetation in the region^[60]^.

The age model for the pollen and charcoal record was recalibrated using the Bacon software and based on the SHCal20 calibration curve^[3]^, with the results providing landscape context for both the MCA20 and Wallen Wallen archaeological data. The palaeoenvironmental record has been discussed in detail in Moss^[59]^.


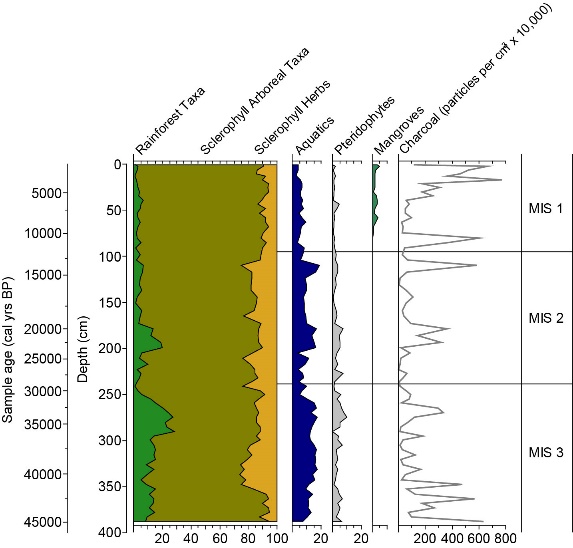


## **Figure S23:** Recalibrated Native Companion Lagoon palaeoecological sequence.

**11. WWC Geophysics**

Ground-penetrating radar (GPR) is a non-invasive geophysical technique that detects buried subsurface features. GPR works by actively pulsing radar waves into the ground. When these waves encounter materials with different physical and chemical properties, a reflection occurs, sending part of the wave back to the surface, where it is received and recorded by the instrument. The remainder of the radar wave continues downward until parts of it are reflected back to the surface by deeper objects or dissipate from being absorbed by subsurface materials. The time between transmission and reception of the radar wave from the antenna to the ground is measured in nanoseconds (ns). Mathematical calculations can approximate the depth at which a reflection occurs. For this project, a Geophysical Survey Systems, Inc. (GSSI) SIR-3000, 400 MHz antenna and a model 620 survey wheel were used to collect the GPR data. Transects were spaced every 0.5 m, and sixteen-bit data were collected with a 40 nS time window, 512 samples/scan and 25 scans/meter. The data were processed (time zero correction, background removal and bandpass filter) and converted into slice maps.

Magnetic gradiometry was used primarily for mapping subsurface magnetic contrasts rich in iron (e.g., ferromagnetic materials) or with thermoremanent magnetisation (TRM)^[61]^. Since magnetometry measures alterations in the Earth’s magnetic field. These features can be geomorphological or human-induced, and their magnetisation can be either remnant or induced^[61]^. The differences in the local field are defined as ‘anomalies,’ and these changes are associated with iron-bearing ferrous material. A Bartington Instruments Fluxgate Grad601-2 was used to collect the magnetic gradiometer data. This instrument utilises four magnetometers—two pairs stacked vertically 1 m apart- to measure the magnetic gradient at each measuring station. Gradiometers allow recording very subtle (0.1 nT) fluctuations in the local magnetic field. The instrument was set up to record data eight times per meter with 0.5 m spaced survey transects (16 samples/m2). Both instruments were used to detect any buried cultural material on the site and the relocation of the previous excavations.

In the GPR data, localised differences in the radar waves were detected. These differences were interpreted based on contrasts and reflectivity differences (either strong or weak amplitude reflections), size, orientation, and shape. GPR reflections with higher contrasts or strong reflections are shown in yellow and red, while weaker reflections are shown in blue. Due to the complex nature of mapped GPR features with depth, an Overlay Analysis was used to combine the amplitude time-slices to show GPR targets of interest. This helps interpret the reflection features as the time slice levels are overlaid to show the most robust reflectors at specified depths^[62]^. The GPR data showed the location of the previous excavation and many high amplitude reflections surrounding it (Fig. S24). The previous excavation pit was easy to detect as it was back filled with lots of sheet metal. Smaller circular amplitude reflections are also present and are likely related to trees based on the reflection profiles and the fact that trees were present in the survey area.

Positive (high values) and negative (low values) response magnetic anomalies were also visible throughout the survey area (Fig. S24). As detected by the GPR, the magnetic gradiometry also mapped the previous excavation pit. Since the site was fenced in the 1980s, the gradiometery also detected the metal pilings and railings associated with the fence. All metal anomalies are dipoles, with lower values to the east and higher values to the west, based on magnetic polarity. Due to the large degree of metal on the site, other features, such as potential hearths or pits were not detected.

To understand the column sample’s stratigraphy, it was important to examine their physical properties in more detail through a series of laboratory analyses, including loss on ignition (LOI), major elemental analyses and low-field magnetic susceptibility (χ). This analysis was achieved by measuring 25 bulk sediment samples collected every 5 cm from WWC’s column sample.

LOI and major elemental measure the amount of organic matter in samples and the major elements present in sediments themselves. These were measured on an Inductively Coupled Plasma Optical Emission Spectrometry (ICP-OES). Magnetic susceptibility was used to help understand the magnetic character of the excavated samples, mainly the presence of iron oxides, which result from their parent material, and the degree to which these oxides have been enhanced by processes associated with human activity (e.g., fires^[63]^). All samples were packed in small non-magnetic Althor P15 boxes (5.28 cm3 volume) and all measurements were completed in the lab using the Bartington Instruments MS2B sensor. Low-field mass-normalised magnetic susceptibility readings (χ) were taken using a 300 A/m field oscillating at both low (460 Hz) and high (4600 Hz) frequencies to calculate the susceptibility. The percentage loss of the low-frequency value (χfd%=(χ460Hz-χ4600Hz /χ460Hz ∗ 100)^[64]^ could not be calculated due to the low magnetic enhancement of all samples.

The magnetic susceptibility (χfd) analysis using only the low-frequency reveals magnetic enhancement in the upper portion of the sedimentary sequence, or the upper 65 cm (Fig. S25). Magnetic enhancement begins to decrease with this depth to about 85 cm, where we see a slight increase in magnetic enhancement. This occurs again at 95 cm below surface; however, these increases are low overall compared to the upper layers with values nearing zero or (10-9 m^3^/kg). Comparison of the magnetic susceptibility to the geochemical analysis reveals a positive correlation between the relative abundance of LOI and elemental material calcium carbonate (CaO), aluminium oxide (Al_2_O_3_)_,_ iron oxide (Fe_2_O_3_), magnesium oxide (Mgo) and manganese oxide (MnO) (Table S23). In general, we see positive correlations between these variables with magnetic susceptibility in the upper layers. This is also where we see increases in heat treated stone artefacts. Other elements such as phosphorus (P), and potassium (K2O) are low or were not present in the samples.

The enhanced signals are likely dependent on both the presence of organic matter and by the relative proportion of aluminium and iron oxide in the sediment. Other elements such as titanium (TiO2) and silicon dioxide (SiO2) were present, especially silicon dioxide with the highest elemental percentage which is to be expected in a sand dune deposits. The correlation with iron oxides might be a form of anthropogenic firing as these minerals can transform into more strongly magnetic minerals by heating^[63,65]^. The lower values for all other variables may be a factor of post-depostional processes such as leaching or the natural divergene to the local geology and sediment elemental composition of the site itself. The high presence of silicon dioxide confirms that a majority of the sedimentary compostion is quartz sand.


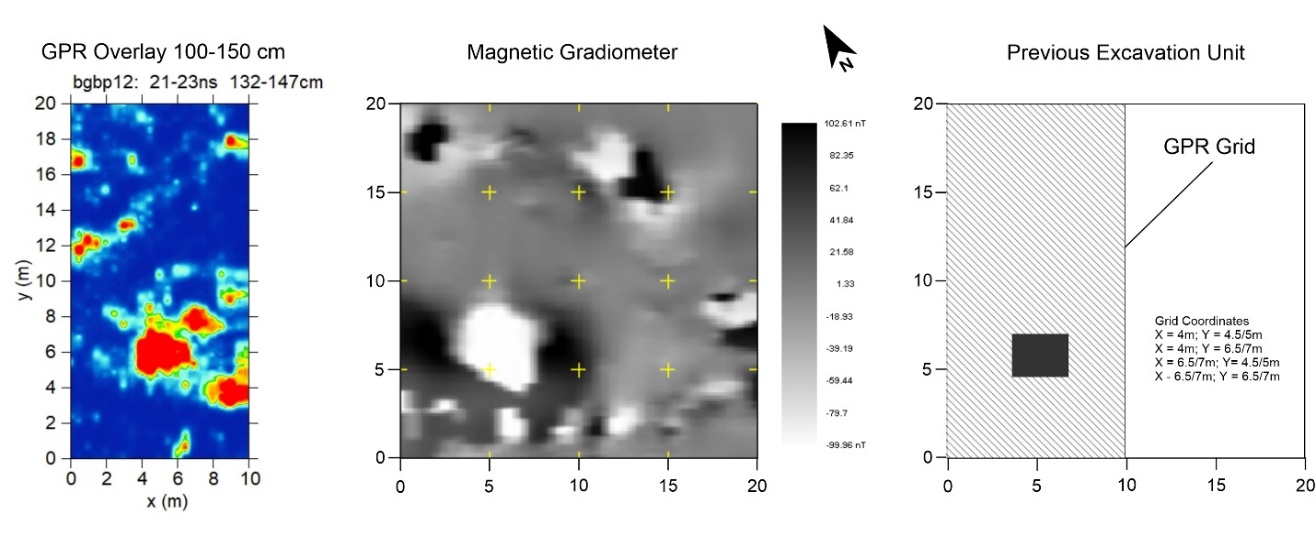


**Figure S24:** GPR slice-map shows high amplitude reflections (left), positive and negative magnetic responses (center), and location of previous excavations.


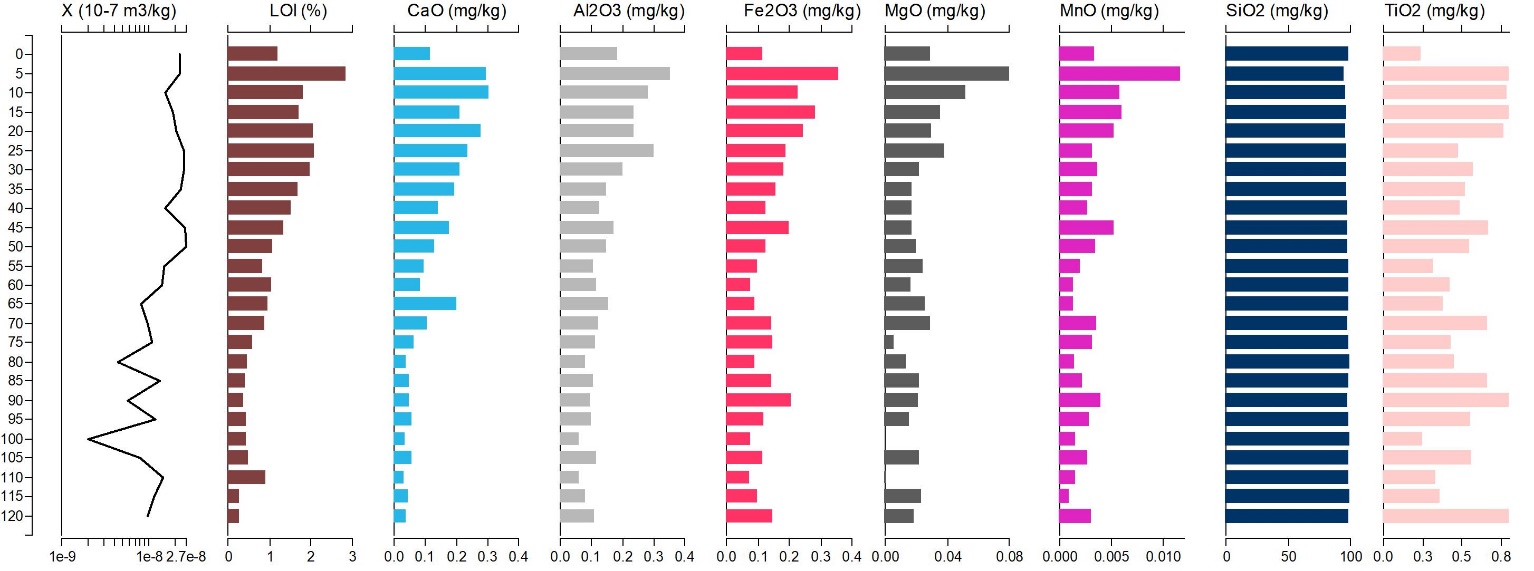


**Figure S25:** Profile of low-field magnetic susceptibility (χlf) with loss on ignition (LOI), calcium equivalent, aluminium and iron oxides, magnesium oxide (Mgo), manganese oxide (MnO), silicon oxide (SiO2) and titanium oxide (TiO2).

**Table S23: Laboratory sediment analysis of WWC**

| **Depth (cm)** | **Mass Susceptibility (m^3/kg)** | **%LOI** | **Al2O3** | **CaO** | **Fe2O3** | **MgO** | **MnO** | **SiO2** | **TiO2** |
| --- | --- | --- | --- | --- | --- | --- | --- | --- | --- |
|  | 2.26E-08 | 1.18 | 0.18 | 0.12 | 0.12 | 0.03 | 0.00 | 97.85 | 0.24 |
| 5 | 2.25E-08 | 2.83 | 0.35 | 0.30 | 0.36 | 0.09 | 0.01 | 94.64 | 0.99 |
| 10 | 1.54E-08 | 1.80 | 0.28 | 0.30 | 0.23 | 0.05 | 0.01 | 95.49 | 0.79 |
| 15 | 1.90E-08 | 1.70 | 0.24 | 0.21 | 0.28 | 0.04 | 0.01 | 96.30 | 1.02 |
| 20 | 2.11E-08 | 2.04 | 0.24 | 0.28 | 0.24 | 0.03 | 0.01 | 95.57 | 0.77 |
| 25 | 2.58E-08 | 2.08 | 0.30 | 0.24 | 0.19 | 0.04 | 0.00 | 96.46 | 0.47 |
| 30 | 2.58E-08 | 1.98 | 0.20 | 0.21 | 0.18 | 0.02 | 0.00 | 96.71 | 0.57 |
| 35 | 2.31E-08 | 1.66 | 0.15 | 0.19 | 0.16 | 0.02 | 0.00 | 96.55 | 0.52 |
| 40 | 1.55E-08 | 1.51 | 0.13 | 0.14 | 0.13 | 0.02 | 0.00 | 97.18 | 0.48 |
| 45 | 2.60E-08 | 1.33 | 0.17 | 0.18 | 0.20 | 0.02 | 0.01 | 97.17 | 0.67 |
| 50 | 2.67E-08 | 1.06 | 0.15 | 0.13 | 0.13 | 0.02 | 0.00 | 97.38 | 0.55 |
| 55 | 1.49E-08 | 0.83 | 0.10 | 0.10 | 0.10 | 0.02 | 0.00 | 98.36 | 0.32 |
| 60 | 1.40E-08 | 1.03 | 0.12 | 0.09 | 0.08 | 0.02 | 0.00 | 97.97 | 0.42 |
| 65 | 8.11E-09 | 0.97 | 0.16 | 0.20 | 0.09 | 0.03 | 0.00 | 97.82 | 0.38 |
| 70 | 9.70E-09 | 0.88 | 0.13 | 0.11 | 0.15 | 0.03 | 0.00 | 97.73 | 0.66 |
| 75 | 1.11E-08 | 0.59 | 0.11 | 0.06 | 0.15 | 0.01 | 0.00 | 98.25 | 0.43 |
| 80 | 4.37E-09 | 0.46 | 0.08 | 0.04 | 0.09 | 0.01 | 0.00 | 98.78 | 0.45 |
| 85 | 1.35E-08 | 0.40 | 0.11 | 0.05 | 0.14 | 0.02 | 0.00 | 98.20 | 0.66 |
| 90 | 5.83E-09 | 0.37 | 0.10 | 0.05 | 0.21 | 0.02 | 0.00 | 97.77 | 1.06 |
| 95 | 1.19E-08 | 0.42 | 0.10 | 0.05 | 0.12 | 0.02 | 0.00 | 98.59 | 0.56 |
| 100 | 1.99E-09 | 0.43 | 0.06 | 0.04 | 0.08 | 0.00 | 0.00 | 99.04 | 0.25 |
| 105 | 7.90E-09 | 0.47 | 0.12 | 0.06 | 0.12 | 0.02 | 0.00 | 98.31 | 0.56 |
| 110 | 1.46E-08 | 0.90 | 0.06 | 0.03 | 0.08 | 0.00 | 0.00 | 98.54 | 0.33 |
| 115 | 1.17E-08 | 0.26 | 0.08 | 0.05 | 0.10 | 0.02 | 0.00 | 98.81 | 0.36 |
| 120 | 9.87E-09 | 0.27 | 0.11 | 0.04 | 0.15 | 0.02 | 0.00 | 98.48 | 0.83 |

**12. Supplementary References**

1. Neal, R. & Stock, E. Pleistocene occupation in the south-east Queensland coastal region. *Nature.* **323**, 618-621 (1986).
2. Gowlett, J. A. J., Hedges, R. E. M., Law, I. A. & Perry, C. Radiocarbon dates from the Oxford AMS system: Archaeometry datelist 5. *Archaeometry*. **29**, 125-55 (1987).
3. Hogg, A. G. *et al.* SHCal20 Southern Hemisphere calibration, 0–55,000 years cal BP. *Radiocarbon*. **62**, 759-778 (2020).
4. Heaton, T. J. *et al.* Marine20—the marine radiocarbon age calibration curve (0–55,000 cal BP). *Radiocarbon*, **62**, 779-820 (2020).
5. Huntley, D. J., Godfrey-Smith, D. I. & Thewalt, M. L. Optical dating of sediments. *Nature.* **313**, 105-107 (1985).
6. Aitken, M. J. *An Introduction to Optical Dating: the Dating of Quaternary Sediments by the Use of Photon-stimulated Luminescence*. (Oxford University Press, 1998).
7. Duller, G. A. T. Luminescence dating of Quaternary sediments: recent advances. *Journal of Quaternary Science.* **19**, 183–192 (2004).
8. Jacobs, Z. & Roberts, R. G. Advances in optically stimulated luminescence dating of individual grains of quartz from archeological deposits. *Evolutionary Anthropology.* **16**, 210–223 (2007).
9. Wintle, A.G., 2014. Luminescence dating methods. In: Holland, H.D., Turekian, K.K. (Eds), Treatise on Geochemistry, vol. 14, pp. 17–35. Elsevier, Oxford.
10. Li, B., Jacobs, Z., Roberts R. G., Galbraith, R. & Peng, J. Variability in quartz OSL signals caused by measurement uncertainties: problems and solutions. *Quaternary Geochronology.* **41**, 11–25 (2017).
11. Roberts, R. G., Jacobs, Z., Li, B., Jankowski, N. R. & Cunningham, A. C. & Rosenfeld, A. B. Optical dating in archaeology: thirty years in retrospect and grand challenges for the future. *Journal of Archaeological Science.* **56**, 41–60 (2015).
12. Jacobs, Z., Duller, G. A. T. & Wintle, A. G. Interpretation of single grain De distributions and calculation of De. *Radiation Measurements.* **41**, 264–277 (2006).
13. Bøtter-Jensen, L., Andersen, C. E., Duller, G. A. T. Murray, A. S. Developments in radiation, stimulation and observation facilities in luminescence measurements. *Radiation Measurements*. **37**, 535–541 (2003).
14. Ballarini, M., Wintle, A. G. & Wallinga, J. Spatial variation of dose rate from beta sources as measured using single grains. *Ancient TL.* **24**, 1-8 (2006).
15. Bøtter-Jensen, L. & Mejdahl, V. Assessment of beta dose-rate using a GM multicounter system. *International Journal of Radiation Applications and Instrumentation*. **14**, 187-191 (1988).
16. Jacobs, Z. & Roberts, R. G. An improved single grain OSL chronology for the sedimentary deposits from Diepkloof Rockshelter, Western Cape, South Africa. *Journal of Archaeological Science.* **63**, 175–192 (2015).
17. Nathan, R. P. & Mauz, B. On the dose-rate estimate of carbonate-rich sediments for trapped charge dating. *Radiation Measurements.* **43**, 14–25 (2008).
18. Brennan, B. J. Beta doses to spherical grains. *Radiation Measurements*. **37**, 299–303 (2003).
19. Bell, W. T. & Zimmerman, D. W. The effect of HF acid etching on the morphology of quartz inclusions for thermoluminescence dating. *Archaeometry.* **20**, 63-65 (1978).
20. Rhodes, E. J. & Schwenninger, J. L. Dose rates and radioisotope concentrations in the concrete calibration blocks at Oxford. *Ancient TL.* **25**, 5–8 (2007).
21. Mercier, N. & Falguères, C. Field gamma dose-rate measurement with a NaI(Tl) detector: reevaluation of the “threshold” technique. *Ancient TL.* **25**, 1–4 (2007).
22. Prescott, J. R. & Hutton, J. T. Cosmic-ray contributions to dose rates for luminescence and ESR dating: large depths and long-term time variations. *Radiation Measurements.* **23**, 497–500 (1994).
23. Brook, B. P., Pietsch, T. J., Olley, J. M., Sloss, C. R. & Cox, M. E. A preliminary OSL chronology for coastal dunes on Moreton Island, Queensland, Australia–Marginal deposits of A large-scale quaternary shelf sediment system. *Continental Shelf Research*. **105**, 79-94 (2015).
24. Lewis, R. J. *et al.* Insights into subtropical Australian aridity from Welsby Lagoon, north Stradbroke Island, over the past 80,000 years. *Quaternary Science Reviews.* **234**, 106262 (2020).
25. Ellerton, D. *et al.* An 800 kyr record of dune emplacement in relationship to high sea level forcing, Cooloola Sand Mass, Queensland, Australia. *Geomorphology*. **354**, 10699 (2020).
26. Galbraith, R. F., Roberts, R. G., Laslett, G. M., Yoshida, H. & Olley, J. M. Optical dating of single and multiple grains of quartz from Jinmium rock shelter, northern Australia: Part I, experimental design and statistical models. *Archaeometry.* **41**, 339–364 (1999).
27. Murray, A. S. & Wintle, A. G. Luminescence dating of quartz using an improved single-aliquot regenerative-dose protocol. *Radiation Measurements.* **32**, 57–73 (2000).
28. Duller, G. A. T. Distinguishing quartz and feldspar in single grain luminescence measurements. *Radiation Measurements.* **37** 161–165 (2003).
29. Balian, H. G. & Eddy, N. W. Figure-of-merit (FOM), an improved criterion over the normalized chi-squared test for assessing goodness-of-fit of gamma-ray spectral peaks. *Nuclear Instruments and Methods*, **145**, 389-395 (1977).
30. Peng, J., Dong, Z. & Han, F. Optically stimulated luminescence dating of sandy deposits from Gulang county at the southern margin of the Tengger Desert, China. *Journal of Arid Land*. **8,** 1-12 (2016).
31. Guralnik, B. *et al.* OSL-thermochronometry of feldspar from the KTB borehole, Germany. *Earth and Planetary Science Letters*. **423** 232-243 (2015).
32. Peng, J., Dong, Z., Han, F., Long, H. & Liu, X. R package numOSL: numeric routines for optically stimulated luminescence dating. *Ancient TL.* **31**, 41–48 (2013).
33. Galbraith, R. F., Roberts, R. G. & Yoshida, H. Error variation in OSL palaeodose estimates from single aliquots of quartz: a factorial experiment. *Radiation Measurements.* **39**, 289–307 (2005).
34. Galbraith, R. F. & Roberts, R. G. Statistical aspects of equivalent dose and error calculation and display in OSL dating: an overview and some recommendations. *Quaternary Geochronology.* **11**, 1–27 (2012).
35. Rousseeuw, P. J. & Croux, C. Alternatives to the median absolute deviation. *Journal of the American Statistical Association.* **88**, 1273–1283 (1993).
36. Rousseeuw, P. J., Debruyne, M., Engelen, S. & Hubert, M. Robustness and outlier detection in chemometrics. *Critical Reviews in Analytical Chemistry.* **36**, 221–242 (2006).
37. Bøtter-Jensen, L., Bulur, E., Duller, G. A. T. & Murray A. S. Advances in luminescence instrument systems. *Radiation Measurements.* **32**, 523-528 (2000).
38. Murray, A. S. & Clemmensen, L. B. Luminescence dating of Holocene aeolian sand movement, Thy, Denmark. *Quaternary Science Reviews.* **20**, 751-754 (2001).
39. Liritzis, I., Stamoulis, K., Papachristodoulou, C. & Ioannides, K. A re-evaluation of radiation dose-rate conversion factors. *Mediterranean Archaeology and Archaeometry.* **13**, 1-15 (2013).
40. Mejdahl, V. Thermoluminescence dating: beta dose attenuation in quartz grains. *Archaeometry*. **21**, 61–72 (1979).
41. Galbraith R. F. & Laslett G. M. Statistical models for mixed fission track ages. *Radiation Measurements.* **21**, 459-470 (1993).
42. Roberts, R. G., Galbraith R. F., Yoshida, H., Laslett, G. M & Olley, J. M. Distinguishing dose populations in sediment mixtures: a test of single-grain optical dating procedures using mixtures of laboratory-dosed quartz. *Radiation Measurements.* **32**, 459-465 (2000).
43. Olley, J. M, Pietsch, T. & Roberts, R. G. Optical dating of Holocene sediments from a variety of geomorphic settings using single grains of quartz. *Geomorphology.* **60**, 337-358 (2004).
44. Pietsch, T. J. Optically stimulated luminescence dating of young (< 500 years old) sediments: Testing estimates of burial dose. *Quaternary Geochronology.* **4**, 406-422 (2009).
45. Pietsch, T. J., Nanson, G. C. & Olley, J. M. Late Quaternary changes in flow-regime on the Gwydir distributive fluvial system, southeastern Australia. *Quaternary Science Reviews.* **69**, 168-180 (2013).
46. Pietsch, T. J. Olley, J. M. & Nanson, G. C. Fluvial transport as a natural luminescence sensitiser of quartz. *Quaternary Geochronology.* **3**, 365-376 (2008).
47. Arnold, L. J. & Roberts, R. G. Stochastic modelling of multi-grain equivalent dose (De) distributions: Implications for OSL dating of sediment mixtures. *Quaternary Geochronology.* **4**, 204-230 (2009).
48. Lomax, J., Hilgers, A., Twidale, C. R., Bourne, J. A. & Radtke, U. Treatment of broad palaeodose distributions in OSL dating of dune sands from the western Murray Basin, South Australia*. Quaternary Geochronology.* **2**, 51-56 (2007).
49. Schmidt, P. How reliable is the visual identification of heat treatment on silcrete? A quantitative verification with a new method. *Archaeological and Anthropological Sciences.* **11**, 713-726 (2019).
50. Schmidt, P. & Hiscock, P. Evolution of silcrete heat treatment in Australia—A regional pattern on the South-East Coast and its evolution over the last 25 ka. *Journal of Paleolithic Archaeology.* **2**, 74-97 (2019).
51. Schmidt, P. & Hiscock, P. The antiquity of Australian silcrete heat treatment: Lake Mungo and the Willandra Lakes. *Journal of Human Evolution*. **142**, 1-13 (2020).
52. Schmidt P. & Hiscock, P. Early silcrete heat treatment in Central Australia: Puritjarra and Kulpi Mara. *Archaeological and Anthropological Sciences.* **12**, 1-7 (2020).
53. Moník, M. Nerudová, Z. & Schnabl, P. Investigation of Heat-Treated Artefacts from Pleistocene Sites. *Journal of Archaeological Science: Reports.* **37**, 102920 (2021).
54. Crabtree D. & Butler, B. Notes on experiments in flintknapping: 1. Heat treatment of silica materials. *Tebiwa.* **7**, 1-6 (1964).
55. Van Dyck, S. & Strahan, R. *The Mammals of Australia* *3^rd^ Edition*. (Reed New Holland, 2008).
56. Aplin, K., Manne, T. & Attenbrow, V. Using a 3-stage burning categorization to assess post-depositional degradation of archaeofaunal assemblages: Some observations based on multiple prehistoric sites in Australasia. *Journal of Archaeological Science: Reports*, **7**, 700-714 (2016).
57. Harris, M., Weisler, M. & Faulkner, P. A refined protocol for calculating MNI in archaeological molluscan shell assemblages: A Marshall Islands case study. *Journal of Archaeological Science.* **57**, 168-179 (2015).
58. Tibby, J. *et al.* Persistence of wetlands on North Stradbroke Island (south‐east Queensland, Australia) during the last glacial cycle: implications for Quaternary science and biogeography*. Journal of Quaternary Science.* **32**, 770-781 (2017).
59. Moss, P. T., Tibby, J., Petherick, L., McGowan, H. & Barr, C. Late Quaternary vegetation history of North Stradbroke Island, Queensland, eastern Australia. *Quaternary Science Reviews.* **74**, 257-272 (2013).
60. Petherick, L., McGowan, H. & Moss, P. Climate variability during the Last Glacial Maximum in eastern Australia: evidence of two stadials. *Journal of Quaternary Science.* **23**, 787-802 (2008).
61. Aspinall A., Gaffney C., & Schmidt A. *Magnetometry for Archaeologists.* (AltaMira Press, 2009).
62. Goodman, D. & Piro, S. *GPR* *Remote Sensing in Archaeology*. (Springer, 2013).
63. Lowe, K. M. *et al.* Using soil magnetic properties to determine the onset of Pleistocene human settlement at Gledswood Shelter 1, Northern Australia. *Geoarchaeology.* **31**, 211–228 (2016).
64. Dearing, J. A. *et al.* Magnetic susceptibility of soil: an evaluation of conflicting theories using a national data set. *Geophysics Journal International.* **127**, 728–734 (1996).
65. Singh, B., O’Connor, S., Veth, P. & Gilkes, R. Detection of amorphous alumino-silicate by X-ray diffraction and chemical analysis to detect firing in archaeological sediments. *Archaeology in Oceania*. **26**(1), 17–20 (1991).
